# Total Synthesis of Cardenolides Acospectoside A and Acovenoside B

**DOI:** 10.3390/molecules30112297

**Published:** 2025-05-23

**Authors:** Benzhang Liu, Peng Xu, Biao Yu

**Affiliations:** 1 School of Chemistry and Materials Science, Hangzhou Institute for Advanced Study, University of Chinese Academy of Sciences, Hangzhou 310024, China; liubenzhang@sioc.ac.cn; 2 State Key Laboratory of Chemical Biology, Shanghai Institute of Organic Chemistry, University of Chinese Academy of Sciences, Chinese Academy of Sciences, Shanghai 200032, China

**Keywords:** cardiac glycoside, total synthesis, cardenolides, glycosylation

## Abstract

Acospectoside A (**1**) and acovenoside B (**2**), two cytotoxic cardenolides extracted from the venomous South African bush *Acokanthera oppositifolia*, are distinguished by their unique structural motifs of the l-acovenose moiety at C-3 and a 1β-*O*-acetylated cardenolide aglycone. Here, we report the synthesis of these cardiac glycosides featuring delicate introductions of the 1-*O*-acetyl group under acid-catalyzed conditions, 14β-OH by Mukaiyama hydration, and a C17-butenolide moiety by Stille coupling.

## 1. Introduction

**Figure 1:**
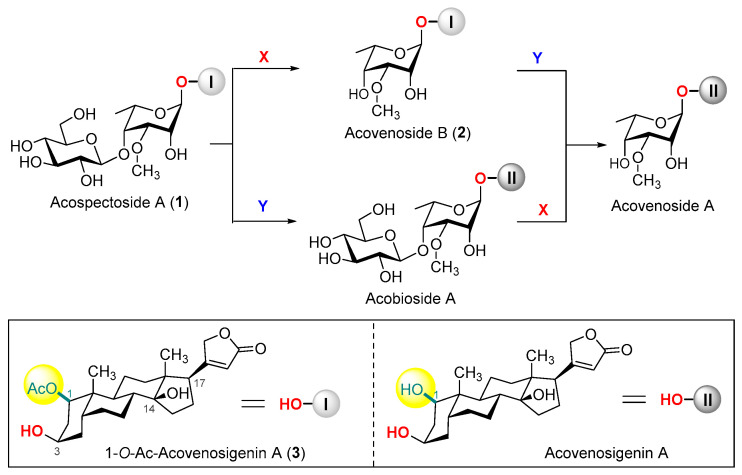
Chemical structures of acospectoside A (**1**), acovenoside B (**2**), acobioside A, acovenoside A, and their conversions. (X = removal of d-glucose with β-glucosidase; Y = deacetylation with esterase).

Cardiac glycosides, a structurally diverse group of natural products, are characterized by a steroid skeleton adorned with a sugar moiety at the C3 position and a lactone moiety at the C17 position [1,2]. For over two centuries, members of this family have been used in clinics for the treatment of heart failure and cardiac rhythm disorders [3,4,5]. Recent investigations have unveiled that diminished levels of specific endogenous cardiotonic steroids could promote tumorigenesis [6], thereby sparking significant interest in a novel hypothesis positing cardiac glycosides as potential anticancer agents [5]. In 1950, avenosides A and B, both sharing the distinctive l-acovenose moiety, were isolated from the South African poisonous bush *Acokanthera oppositifolia* [7]. Subsequent phytochemistry research paved the way for the isolation and characterization of acospectoside A and acobioside A (Figure 1) [8,9]. Notably, acospectoside A (**1**) could be metabolically converted to acovenoside B (**2**), acobioside A, and acovenoside A through enzymatic treatment with a snail (*Helix pomatia*) enzyme (Figure 1) [10,11]. These cardiac glycosides were further evaluated for their cardiotoxicity in cats and cytotoxicity against human carcinoma of the nasopharynx cells [10]. These findings indicated that the C-1 acetoxylated glycosides exhibited reduced activity compared to their hydroxylated counterparts, while monosides demonstrated greater cytotoxicity than the corresponding biosides. Amongst these cardiac glycosides, acovenoside A emerged as a potent inhibitor of the proliferation of human non-small-cell lung cancer (NSCLC) A-549 cells with an IC_50_ value of 68 nM, outperforming the anticancer drug doxorubicin (IC_50_ = 426 nM) [12]. However, the limited accessibility of these heterogeneous metabolites from natural sources has impeded in-depth investigations into their pharmaceutical potential and therapeutic applications. The chemical synthesis of these highly intricate cardiac glycosides presents a highly effective and transformative approach, offering a scalable and reproducible avenue for accessing these bioactive natural products and their structurally optimized derivatives [13,14,15,16]. Very recently, we developed a convenient approach to the first chemical synthesis of acovenoside A, oppofrioside, euonymuside A, and their congeners [17]. Here, we disclose the first total synthesis of acospectoside A (**1**) and acovensoide B (**2**), with 1-*O*-Ac-acovenosigenin A (**3**) as a common aglycone.

## 2. Results

**Scheme 1:**
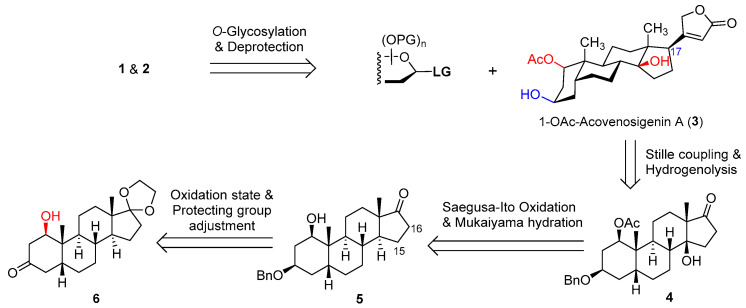
Retrosynthetic plan for acospectoside A (**1**) and acovenoside B (**2**).

Building upon our prior investigations into the synthesis of steroid glycosides [17,18,19], we envisioned the assembly of acovenosides (**1**, **2**) through late-stage glycosylation, employing 1-*O*-Ac-acovenosigenin A (**3**) and glycosyl imidate donors (Scheme 1). However, our initial endeavors to achieve the selective acetylation of acovenosigenin A were unsuccessful in yielding the desired aglycone **3**. Consequently, a retrosynthetic analysis was undertaken, revealing that aglycone **3** could be elaborated on from the known intermediate (**6**) [17] via the introduction of the 1-*O*-acetyl group, modulating oxidation states at C-14, and installing the butenolide ring at C-17. A reduction of the 3-ketone group, followed by selective benzylation, led to the formation of intermediate **5**. This intermediate was then transformed into 17-ketone **4** through a sequence involving Saegusa–Ito oxidation and Mukaiyama hydration. Finally, the introduction of the C-17 butenolide moiety and selective debenzylation afforded 1-OAc-acovenosigenin A (**3**). The timing of introducing the 1-*O*-acetyl group is of strategic importance, and a successful synthetic route could be determined by trial-and-error.

**Scheme 2:**
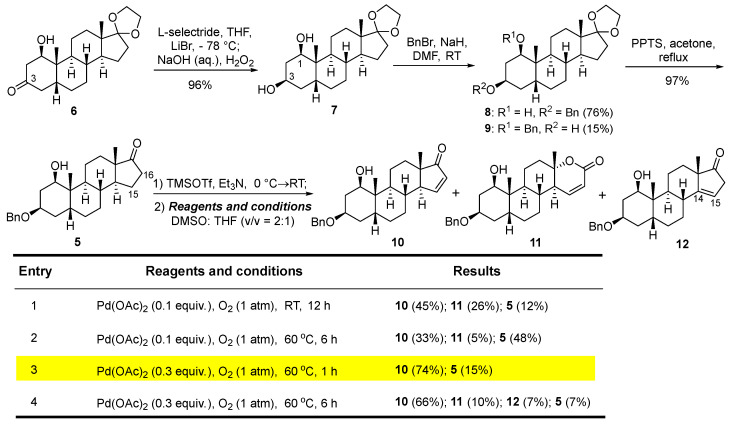
Synthesis of enone **10**.

The selective reduction of 3-ketone **6** with l-selectride [20], followed by oxidative cleavage of the resulting boronic ester with a sodium hydroxide solution and hydrogen peroxide afforded 1β,3β-diol **7** (96%) (Scheme 2). Selective 3-*O*-benzylation with benzyl bromide and sodium hydride delivered the desired 1-ol **8** (76%), alongside 3-ol isomer **9** (15%). Acidic hydrolysis of the C17-ethylene glycol ketal in **8** furnished 17-ketone **5** (82%). The treatment of ketone **5** with TMSOTf and triethylamine generated the corresponding trimethylsilylenol ether. Subsequent palladium-mediated Saegusa–Ito oxidation, utilizing O_2_ as a stoichiometric oxidant to form the 15,16-unsaturated enone **10**, required extensive optimization. The conventional Saegusa–Ito oxidation protocol typically necessitates a superstoichiometric quantity of palladium(II) acetate to achieve satisfactory yields [21]. In 1995, Larock and co-workers pioneered a catalytic variant of Saegusa–Ito oxidation using palladium(II) acetate under an oxygen atmosphere [22]. Applying Larock’s protocol to the cyclic silylenol ether, with catalytic palladium(II) acetate (0.1 equiv.) and O_2_ as the reoxidant in DMSO, afforded the desired 15,16-unsaturated enone **10** in a moderate 45% yield, alongside 12% recycled **5** (attributed to the hydrolysis of the trimethylsilyl enol ether). Concurrently, lactone **11** was isolated as a side product (26%), arising from a Baeyer–Villiger-type rearrangement of the 17-ketone followed by the unsaturation of the α,β-position of carbonyl via β-hydride elimination of palladium in one-pot [23]. Given that elevating the temperature could mitigate the formation of lactone byproducts, the Saegusa–Ito oxidation was conducted at 60 °C, yielding enone **10** (33%) and lactone **11** (5%), though nearly half of 17-ketone **5** was recycled. To enhance the conversion rate, the amount of palladium(II) acetate was further increased to 0.3 equivalents. Optimal results were achieved within 1 h, delivering enone **10** in a good 74% yield, with 15% of 17-ketone **5** recycled. Extending the reaction time to 6 h only marginally improved the conversion rate, with 7% of 17-ketone **5** recycled, while enone **10** (33%), lactone **11** (5%), and unconjugated Δ^14,15^ **12** (7%) were also obtained.

**Scheme 3:**
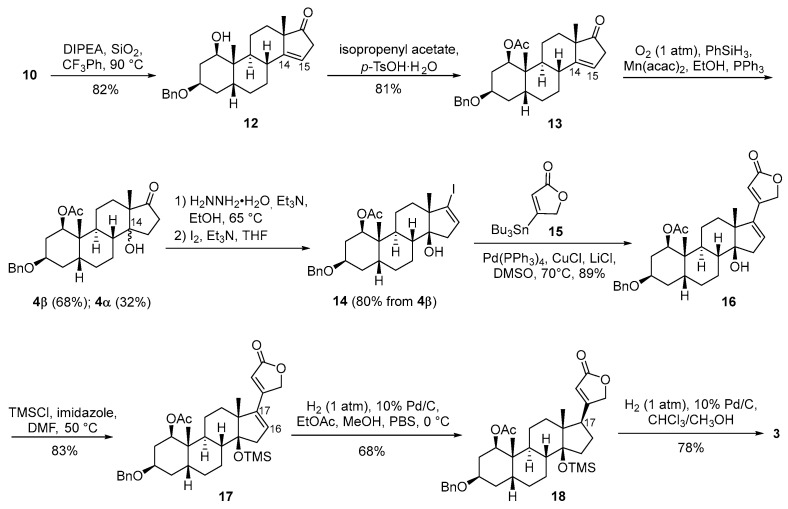
Synthesis of 1-*O*-Ac-acovenosigenin A (**3**).

The isomerization of **10** was carried out in the presence of *N*,*N*-diisopropylethylamine and silicon dioxide at 90 °C, yielding unconjugated Δ^14,15^ derivative **12** (82%) (Scheme 3). The strategic introduction of the 1-*O*-acetyl group was successfully achieved by employing isopropenyl acetate and *p*-toluenesulfonic acid monohydrate, resulting in the formation of the fully protected intermediate **13** (81%). Mukaiyama hydration of Δ^14,15^ **13**, utilizing Mn(acac)_2_ and PhSiH_3_ under aerobic conditions, furnished tertiary alcohols **4β** (68%) and **4α** (32%) on a gram scale, showcasing the efficiency and scalability of the reaction [24]. 17-Ketone **4β** was subsequently converted to vinyl iodide **14** (81%) with hydrazine hydrate and iodine. The Stille coupling reaction of vinyl iodide **14** with the known stannylated butanolide **15** catalyzed by Pd(PPh_3_)_4_ proceeded effectively, affording the desired cardiac steroid **16** in an excellent 89% yield [19]. The direct hydrogenation of the Δ^16,17^ double bond in **16** posed a risk of generating C-17 isomers. To circumvent this, the 14β-OH group in **16** was protected with TMSCl and imidazole, yielding intermediate **17** (83%). The subsequent hydrogenation of the Δ^16,17^ double bond in **17** proceeded smoothly on a gram scale, leading to the fully protected aglycone **18** (68%). Finally, the desired aglycone **3** was obtained through Pd/C-catalyzed debenzylation under atmospheric hydrogen in a mixed solvent of chloroform and methanol (78%). This sequence of reactions highlights the meticulous planning and optimization required to achieve the targeted cardiac steroid with high efficiency and selectivity.

**Scheme 4:**
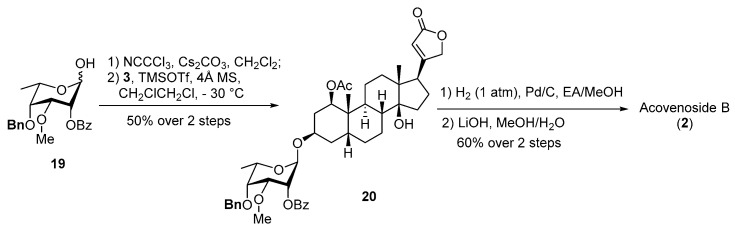
Synthesis of acovenoside B (**2**).

In 1969, Kapadia speculated that the observed difficulty of the saponification of 3-*O*-acetyl groups in acospectoside A (**1**) and acovenoside B (**2**) could likely be attributed to the steric hindrance imposed by an axially bound acetoxyl group [10]. The subsequent experimental transformations revealed that acetyl groups situated within the equatorial positions of the carbohydrate moieties could be selectively removed through mild saponification using potassium bicarbonate, without any discernible impact on the 3-*O*-acetyl groups in the aglycone moieties [10]. With the desired aglycone derivative **3** and monosaccharide building blocks in hand, we embarked on the assembly of the target cardiac glycosides (Scheme 4). The known lactol **19** was transformed into an imidate donor with trichloroacetonitrile and cesium carbonate. The resulting reactive intermediate was promptly subjected to glycosylation with aglycone **3**, promoted by TMSOTf, to afford glycoside **20** in a 50% yield over a two-step sequence. Finally, hydrogenative debenzylation and selective debenzoylation of the sugar residue were executed, culminating in the successful synthesis of acovenoside B (**2**) in a satisfactory 60% yield.

**Scheme 5:**
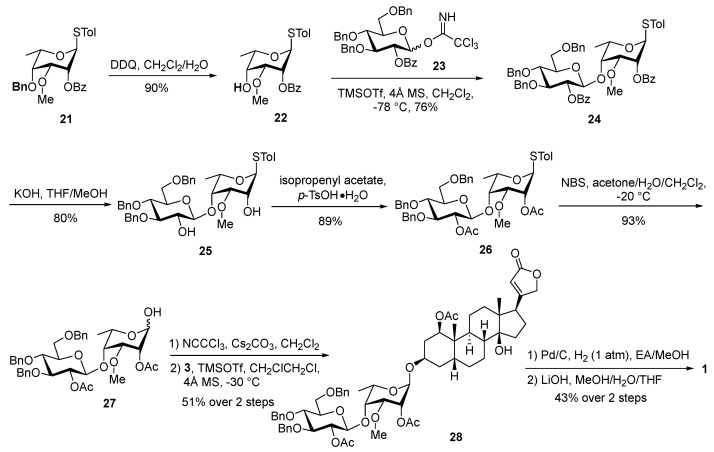
Synthesis of acospectoside A (**1**).

The known thioglycoside **21** underwent efficient debenzylation upon treatment with 2,3-dichloro-5,6-dicyano-*p*-benzoquinone (DDQ), yielding 4-ol **22** (90%). The condensation of **22** with imidate **23** was complete within 2 h catalyzed by TMSOTf at −78 °C, and the desired disaccharide **24** was obtained in a good 76% yield (Scheme 5). Recognizing that debenzoylation posed a greater synthetic challenge than deacetylation, we strategically converted the benzoyl protecting groups in **24** to acetyl groups through a meticulous two-step sequence involving saponification and subsequent acetylation with isopropenyl acetate, affording disaccharide **26**. The selective hydrolysis of the anomeric thio group was achieved using *N*-bromosuccinimide (NBS), furnishing lactol **27** in an excellent 93% yield. Lactol **27** was then condensed with trichloroacetonitrile in the presence of cesium carbonate to generate the disaccharide imidate donor, which was immediately coupled with aglycone **3** to produce bioside **28** (51% over two steps). Finally, hydrogenative debenzylation and selective deacetylation of the disaccharide residue furnished acospectoside A (**1**), with no influence on the 1-*O*-acetyl group. The NMR data of the synthetic acospectoside A (**1**) and acovenoside B (**2**) were identical to those reported for the natural products (see Tables S1 and S2 for details) [25].

## 3. Experimental Section

### 3.1. General Information

Commercial reagents were used without further purification and made in China unless specified. Crushed 4 Å molecular sieves (MSs) were activated through flame-drying under high vacuum conditions immediately prior to use. Dry CH_2_Cl_2_, DMF, and toluene were obtained by drying with activated MSs. Dry pyridine and NEt_3_ were obtained by drying with anhydrous KOH. Anhydrous THF was obtained by refluxing with Na under an argon atmosphere. Thin layer chromatography (TLC) was performed on TLC silica gel 60 F254 (Merck, Darmstadt, Germany). The TLC plates were visualized with UV light and/or by staining with EtOH/H_2_SO_4_ (10%, *v*/*v*). Flash column chromatography was performed on silica gel, SiliaFlash P60 (40–63 μm, Silicycle, Quebec, QC, Canada). The NMR spectra were measured on a Bruker AM 400 (Zurich, Switzerland), Agilent 500 MHz (Santa Clara, CA, USA) NMR spectrometer at 25 °C. ^1^H and ^13^C NMR signals were calibrated to the residual proton and carbon resonance of the solvent (CDCl_3_: *δ*_H_ = 7.26 ppm, *δ*_C_ = 77.16 ppm; CD_3_OD: *δ*_H_ = 3.31 ppm, *δ*_C_ = 49.00 ppm; C_5_D_5_N: *δ*_H_ =8.74 ppm, *δ*_C_ = 155.35 ppm). High-resolution mass spectra were recorded with Shimadzu Biotech Axima Performance FTMS (Kyoto, Japan), maXis 4G FTMS, Thermo Scientific Q Exactive HF Orbitrap-FTMS (Waltham, MA, USA), or Agilent-TOF/LC-MS 1260-6230 FTMS. Optical rotations were measured on an Anton Paar MCP5500 polarimeter (Graz, Austria).

### 3.2. Synthesis of Compounds ***1***–***28***

#### 3.2.1. Synthesis of Compound **7**

To a mixture of compound **6** (5.2 g, 14.9 mmol, 1.0 equiv.) and LiBr (7.8 g, 89.5 mmol, 6.0 equiv.), dry THF (150 mL) was added under an atmosphere of Ar. l-selectride (22.4 mL, 1 M, 1.5 equiv., 0.5 mL/min) was added dropwise at −78 °C to the resulting mixture under an Ar atmosphere and stirred for 2 h at the same temperature. TLC indicated the reaction was complete (petroleum ether/EtOAc = 2/1). The reaction mixture was quenched with a 2 N NaOH aqueous solution (75 mL) and 30% H_2_O_2_ (7.5 mL). The mixture was extracted with EtOAc (150 mL × 3), and the organic phase was washed with brine, and dried with Na_2_SO_4_. After filtration, the solution was concentrated under reduced pressure. The residue was purified by silica gel column chromatography (petroleum ether/EtOAc/CH_2_Cl_2_ = 2/1/0.6) to give compound **7** (5.0 g, 96%) as a white amorphous solid. [α${]}_{D}^{25}$ = −19.6 (*c* 1.0, CHCl_3_); ^1^H NMR (600 MHz, CDCl_3_) δ 4.13 (s, 1H), 3.95–3.79 (m, 5H), 3.42 (s, 2H), 2.09–1.89 (m, 4H), 1.86–1.69 (m, 3H), 1.68–1.60 (m, 1H), 1.53–1.31 (m, 7H), 1.28–1.18 (m, 4H), 1.15–1.10 (m, 1H), 1.09 (s, 3H), 0.82 (d, *J* = 1.7 Hz, 3H); ^13^C NMR (125 MHz, CDCl_3_) δ 119.6, 74.0, 68.5, 65.3, 64.6, 50.5, 45.9, 41.9, 39.8, 36.2, 34.2, 33.7, 32.3, 31.0, 30.7, 26.0, 25.3, 22.7, 20.5, 19.0, 14.6; HR-ESI-MS (*m*/*z*) calcd for C_21_H_34_O_4_Na [M+Na]^+^: 373.2349, found: 373.2347.

#### 3.2.2. Synthesis of Compound **8** and **9**

To a stirring solution of compound **7** (5.0 g, 14.4 mmol, 1.0 equiv.) and BnBr (1.7 mL, 14.4 mmol, 1.0 equiv.) in dry DMF (140 mL), 60% NaH (1.4 g, 36.0 mmol, 2.5 equiv.) was added in portions at room temperature. And the reaction mixture was stirred for 2 h at the same temperature. TLC indicated the reaction was complete. The reaction mixture was quenched with a saturated NH_4_Cl solution (20 mL). H_2_O (100 mL) was added to the mixture; the mixture extracted with EtOAc (300 mL), and the organic phase was washed with brine, and dried with Na_2_SO_4_. After filtration, the solution was concentrated under reduced pressure. The residue was purified by silica gel column chromatography (petroleum ether/EtOAc = 10/1 → 4/1) to give compound **8** (4.8 g, 76%) and to give compound **9** (923 mg, 15%) as white amorphous solids.

**8**: [α${]}_{D}^{25}$ = −19.0 (*c* 1.0, CHCl_3_); ^1^H NMR (500 MHz, CDCl_3_) δ 7.37–7.26 (m, 5H), 4.56–4.48 (m, 2H), 3.99 (d, *J* = 9.7 Hz, 1H), 3.95–3.82 (m, 6H), 3.71 (dt, *J* = 9.7, 3.0 Hz, 1H), 2.10 (dq, *J* = 14.8, 2.8 Hz, 1H), 2.02–1.92 (m, 2H), 1.91–1.86 (m, 1H), 1.86–1.74 (m, 2H), 1.70 (dt, *J* = 15.2, 3.1 Hz, 1H), 1.67–1.59 (m, 2H), 1.55–1.36 (m, 6H), 1.34–1.19 (m, 4H), 1.10 (s, 3H), 0.84 (s, 3H); ^13^C NMR (125 MHz, CDCl_3_) δ 138.1, 128.6, 127.8, 127.7, 119.6, 76.0, 73.1, 70.7, 65.3, 64.6, 50.6, 45.9, 42.1, 40.0, 36.2, 34.2, 31.1, 31.0, 30.1, 29.9, 26.2, 25.3, 22.8, 20.4, 18.9, 14.6; HR-ESI-MS (*m*/*z*) calcd for C_28_H_40_O_4_Na [M+Na]^+^: 463.2819, found: 463.2823.

**9**: [α${]}_{D}^{25}$ = −42.1 (*c* 1.0, CHCl_3_); ^1^H NMR (500 MHz, CDCl_3_) δ 7.36–7.31 (m, 4H), 7.31–7.27 (m, 1H), 4.69 (d, *J* = 11.0 Hz, 1H), 4.40 (d, *J* = 11.0 Hz, 1H), 4.33 (d, *J* = 10.4 Hz, 1H), 3.98 (dt, *J* = 10.6, 3.0 Hz, 1H), 3.94–3.81 (m, 4H), 3.60 (s, 1H), 2.16–2.04 (m, 2H), 2.02–1.91 (m, 2H), 1.86–1.73 (m, 2H), 1.70–1.59 (m, 2H), 1.55–1.35 (m, 6H), 1.32–1.20 (m, 5H), 1.11 (s, 3H), 0.84 (s, 3H); ^13^C NMR (125 MHz, CDCl_3_) δ 138.0, 128.6, 128.1, 127.9, 119.5, 82.8, 73.0, 68.0, 65.3, 64.6, 50.6, 45.9, 41.6, 40.2, 36.0, 34.2, 33.9, 31.3, 30.9, 28.6, 25.9, 25.2, 22.7, 20.7, 18.9, 14.6; HR-ESI-MS (*m*/*z*) calcd for C_28_H_40_O_4_Na [M+Na]^+^: 463.2819, found: 463.2822.

#### 3.2.3. Synthesis of Compound **5**

To a stirring solution of **8** (4.7 g, 10.7 mmol, 1.0 equiv.) in acetone (11 mL), pyridinium *p*-toluenesulfonate (4.0 g, 16.1 mmol, 1.5 equiv.) was added. The resulting mixture was heated to reflux for 3 h. TLC indicated the reaction was complete. The reaction mixture was concentrated under reduced pressure and added to a 1 N HCl aqueous solution (100 mL). The resulting mixture was extracted with CH_2_Cl_2_ (100 mL × 3); the combined organic phase was washed with brine, and dried with Na_2_SO_4_. After filtration, the solution was concentrated under reduced pressure. The residue was purified by silica gel column chromatography (petroleum ether/EtOAc = 10/1) to give **5** (4.1 g, 97%) as a white amorphous solid. [α${]}_{D}^{25}$ = +55.6 (*c* 1.0, CHCl_3_); ^1^H NMR (500 MHz, CDCl_3_) δ 7.36–7.26 (m, 5H), 4.58–4.49 (m, 2H), 4.03 (d, *J* = 9.7 Hz, 1H), 3.93–3.87 (m, 1H), 3.71 (dt, *J* = 9.8, 3.1 Hz, 1H), 2.43 (ddd, *J* = 19.1, 8.9, 1.1 Hz, 1H), 2.12 (dq, *J* = 14.9, 2.9 Hz, 1H), 2.07–2.00 (m, 2H), 1.96–1.85 (m, 3H), 1.81 (dt, *J* = 12.8, 3.0 Hz, 1H), 1.73–1.62 (m, 3H), 1.60–1.55 (m, 1H), 1.51 (ddd, *J* = 12.5, 9.1, 3.4 Hz, 1H), 1.48–1.42 (m, 1H), 1.40–1.29 (m, 3H), 1.28–1.15 (m, 3H), 1.13 (s, 3H), 0.86 (s, 3H); ^13^C NMR (125 MHz, CDCl_3_) δ 221.3, 138.0, 128.6, 128.6, 127.9, 127.7, 75.8, 72.9, 70.8, 51.5, 47.7, 42.4, 40.2, 35.9, 35.6, 31.9, 31.1, 30.0, 26.1, 25.0, 21.9, 20.2, 18.9, 14.0; HR-ESI-MS (*m*/*z*) calcd for C_26_H_36_O_3_Na [M+Na]^+^: 419.2557, found: 419.2553.

#### 3.2.4. Synthesis of Compounds **10** and **11**

To a stirring solution of **5** (115 mg, 0.290 mmol, 1.0 equiv.) in dry CH_2_Cl_2_ (6 mL), NEt_3_ (0.161 mL, 1.02 mmol, 4.0 equiv.), and TMSOTf (0.185 mL, 0.185 mmol, 3.5 equiv.) were added successively at 0 °C under an Ar atmosphere. The mixture was gradually warmed up to room temperature and stirred for 1 h. TLC indicated the reaction was complete. The reaction mixture was quenched with a saturated NaHCO_3_ solution (15 mL) at 0 °C and extracted with CH_2_Cl_2_ (15 mL × 3); the combined organic phase was washed with brine, and dried with Na_2_SO_4_. After filtration, the solution was concentrated under reduced pressure. The resulting residue was dissolved in dry DMSO (4 mL) and THF (2 mL). Pd(OAc)_2_ (6.5 mg, 0.029 mmol, 0.1 equiv.) was added to the solution. The mixture was heated to 60 °C for 12 h under an O_2_ atmosphere. TLC indicated the reaction was complete (petroleum ether/EtOAc = 2/1). The reaction mixture was added to a saturated NH_4_Cl solution (20 mL) and extracted with EtOAc (20 mL × 3); the combined organic phase was washed with an aqueous NH_4_Cl, brine, and dried with Na_2_SO_4_. After filtration, the solution was concentrated under reduced pressure. The residue was dissolved with acetonitrile (20 mL). To the solution, FeCl_3_ (49 mg, 0.304 mmol, 1.05 equiv.) was added at 0 °C and stirred for 10 min. The mixture was neutralized by K_2_CO_3_ (120 mg, 0.870 mmol, 3.0 equiv.) and filtered; the filtrate was concentrated under reduced pressure and purified by silica gel column chromatography (petroleum ether/EtOAc = 6/1 → 4/1) to give **10** (51 mg, 45%), **11** (31 mg, 26%), and **5** (14 mg, 12%) as white amorphous solids.

**10**: [α${]}_{D}^{25}$ = −55.2 (*c* 1.0, CHCl_3_); ^1^H NMR (400 MHz, CDCl_3_) δ 7.52 (dd, *J* = 6.0, 1.8 Hz, 1H), 7.38–7.27 (m, 5H), 6.02 (dd, *J* = 5.9, 3.1 Hz, 1H), 4.59–4.50 (m, 2H), 4.09 (d, *J* = 9.6 Hz, 1H), 3.91 (q, *J* = 3.0 Hz, 1H), 3.72 (dt, *J* = 9.7, 3.0 Hz, 1H), 2.32 (ddd, *J* = 11.4, 3.1, 1.9 Hz, 1H), 2.15 (dq, *J* = 14.9, 2.9 Hz, 1H), 2.11–2.03 (m, 1H), 1.99–1.85 (m, 4H), 1.81–1.73 (m, 1H), 1.71–1.64 (m, 2H), 1.57–1.45 (m, 3H), 1.42–1.24 (m, 3H), 1.18 (s, 3H), 1.07 (s, 3H); ^13^C NMR (100 MHz, CDCl_3_) δ 213.3, 158.6, 137.9, 131.8, 128.6, 127.9, 127.7, 75.7, 72.7, 70.8, 57.0, 51.0, 43.7, 40.4, 33.0, 31.0, 29.9, 29.8, 29.4, 26.0, 25.1, 20.8, 19.9, 18.8; HR-ESI-MS (*m*/*z*) calcd for C_26_H_34_O_3_Na [M+Na]^+^: 417.2400, found: 417.2387.

**11:** [α${]}_{D}^{25}$ = −24.2 (*c* 1.0, CHCl_3_); ^1^H NMR (500 MHz, CDCl_3_) δ 7.38–7.27 (m, 5H), 6.81 (dd, *J* = 9.9, 2.2 Hz, 1H), 6.02 (dd, *J* = 9.8, 3.0 Hz, 1H), 4.59–4.49 (m, 2H), 3.95–3.90 (m, 1H), 3.72 (d, *J* = 3.1 Hz, 1H), 2.33 (dt, *J* = 11.4, 2.5 Hz, 1H), 2.18–2.12 (m, 1H), 2.09–2.02 (m, 1H), 1.99 (dt, *J* = 12.6, 3.5 Hz, 1H), 1.93–1.82 (m, 3H), 1.76 (dd, *J* = 13.2, 4.4 Hz, 1H), 1.72 (s, 2H), 1.64–1.58 (m, 1H), 1.59–1.48 (m, 1H), 1.49–1.40 (m, 1H), 1.41–1.35 (m, 1H), 1.32 (s, 3H), 1.28–1.21 (m, 1H), 1.18 (dd, *J* = 12.2, 4.0 Hz, 1H), 1.09 (s, 3H); ^13^C NMR (125 MHz, CDCl_3_) δ 164.0, 145.4, 137.9, 128.6, 127.9, 127.7, 121.8, 83.3, 75.6, 72.5, 70.9, 48.6, 41.9, 40.2, 38.4, 35.8, 30.6, 30.0, 29.8, 25.9, 24.6, 21.7, 18.7, 18.6; HR-ESI-MS (*m*/*z*) calcd for C_26_H_35_O_4_ [M+H]^+^: 411.2530, found: 411.2523.

To a stirring solution of **5** (15.0 g, 37.8 mmol, 1.0 equiv.) in dry CH_2_Cl_2_ (120 mL), NEt_3_ (21.0 mL, 151 mmol, 4.0 equiv.), and TMSOTf (23.9 mL, 132 mmol, 3.5 equiv.) were added successively at 0 °C under an Ar atmosphere. The mixture was gradually warmed up to room temperature and stirred for 1 h. TLC indicated the reaction was complete. The reaction mixture was quenched with a saturated NaHCO_3_ solution (100 mL) at 0 °C and the aqueous phase was extracted with CH_2_Cl_2_ (100 mL × 2). The combined organic phase was washed with brine, and dried with Na_2_SO_4_. After filtration, the solution was concentrated under reduced pressure. The resulting residue was dissolved in dry DMSO (280 mL) and THF (140 mL). Pd(OAc)_2_ (2.5 g, 11.3 mmol, 0.3 equiv.) was added to the solution. The mixture was heated to 60 °C for 12 h under an O_2_ atmosphere. TLC indicated the reaction was complete (petroleum ether/EtOAc = 2/1). FeCl_3_ (6.4 g, 39.7 mmol, 1.05 equiv.) was added to the reaction mixture at 0 °C and stirred for 10 min. The mixture was neutralized by K_2_CO_3_ (16.4 g, 119.0 mmol) and filtered; the filtrate was concentrated under reduced pressure and purified by silica gel column chromatography (petroleum ether/EtOAc/CH_2_Cl_2_ = 8/1/0.9 → 4/1/0.9) to give **10** (11.0 g, 74%) and **5** (2.3 g, 15%) as white amorphous solids.

#### 3.2.5. Synthesis of Compound **12**

To a mixture of **10** (2.9 g, 7.40 mmol, 1.0 equiv.) and SiO_2_ (8.0 g), benzotrifluoride (80 mL) and DIPEA (67.3 mL, 407 mmol, 55.0 equiv.) were added successively under an atmosphere of Ar. The resulting mixture was stirred for 2 h at 90 °C under an Ar atmosphere. TLC indicated the reaction was complete (petroleum ether/EtOA = 2/1). The reaction mixture was filtered, and the filter cake was washed with EtOA (150 mL). The filtrate was concentrated under reduced pressure. The residue was dissolved with CH_2_Cl_2_ (100 mL), and the organic phase was washed with an aqueous 5 wt% HCl solution, brine, and dried with Na_2_SO_4_. After filtration, the solution was concentrated under reduced pressure. The residue was purified by silica gel column chromatography (petroleum ether/EtOAc = 10:1) to give **12** (2.4 g, 82%) as a white amorphous solid. [α${]}_{D}^{25}$ = +84.3 (*c* 1.0, CHCl_3_); ^1^H NMR (400 MHz, CDCl_3_) δ 7.39–7.27 (m, 5H), 5.50 (q, *J* = 2.1 Hz, 1H), 4.59–4.48 (m, 2H), 4.03 (d, *J* = 9.7 Hz, 1H), 3.89 (t, *J* = 2.9 Hz, 1H), 3.73 (d, *J* = 9.3 Hz, 1H), 3.00 (ddd, *J* = 23.1, 3.9, 1.9 Hz, 1H), 2.82 (dt, *J* = 23.1, 2.3 Hz, 1H), 2.26 (ddd, *J* = 13.6, 9.0, 4.9 Hz, 1H), 2.16–2.09 (m, 1H), 2.07–2.01 (m, 1H), 1.99–1.83 (m, 2H), 1.78 (dt, *J* = 12.9, 3.2 Hz, 1H), 1.70–1.58 (m, 3H), 1.55–1.48 (m, 1H), 1.48–1.42 (m, 1H), 1.41–1.34 (m, 1H), 1.29–1.19 (m, 2H), 1.15 (s, 3H), 1.11 (s, 3H); ^13^C NMR (100 MHz, CDCl_3_) δ 153.6, 138.0, 128.6, 127.9, 127.7, 113.1, 75.7, 72.7, 70.8, 50.9, 43.0, 41.5, 40.4, 35.8, 33.5, 31.1, 30.3, 29.8, 25.8, 22.7, 20.8, 20.1, 18.8; HR-ESI-MS (*m*/*z*) calcd for C_26_H_34_O_3_Na [M+Na]^+^: 417.2400, found: 417.2411.

#### 3.2.6. Synthesis of Compound **13**

To a stirring solution of compound **12** (2.4 g, 6.03 mmol, 1.0 equiv.) in isopropenyl acetate (12 mL), TsOH·H_2_O (4.6 g, 24.1 mmol, 4.0 equiv.) was added at room temperature. After, the reaction mixture was stirred for 10 min at the same temperature. TLC indicated the reaction was complete. CH_2_Cl_2_ (100 mL) was added, and the reaction mixture was quenched with a saturated NaHCO_3_ solution (100 mL) at 0 °C. The organic phase was washed with brine, and dried with Na_2_SO_4_. After filtration, the solution was concentrated under reduced pressure. The residue was purified by silica gel column chromatography (petroleum ether/EtOAc = 10/1) to give compound **13** (2.14 g, 81%) as a white amorphous solid. [α${]}_{D}^{25}$ = +51.9 (*c* 1.0, CHCl_3_); ^1^H NMR (500 MHz, CDCl_3_) δ 7.34–7.29 (m, 4H), 7.26–7.22 (m, 1H), 5.52 (q, *J* = 2.1 Hz, 1H), 4.97 (t, *J* = 3.0 Hz, 1H), 4.47–4.39 (m, 2H), 3.73 (q, *J* = 3.0 Hz, 1H), 3.00 (ddd, *J* = 23.0, 3.8, 1.8 Hz, 1H), 2.82 (dt, *J* = 23.1, 2.2 Hz, 1H), 2.32–2.19 (m, 3H), 1.99–1.95 (m, 1H), 1.94 (s, 3H), 1.93–1.87 (m, 2H), 1.80 (dt, *J* = 13.1, 3.1 Hz, 1H), 1.67–1.61 (m, 3H), 1.55 (dt, *J* = 15.7, 3.3 Hz, 1H), 1.50–1.31 (m, 3H), 1.23 (td, *J* = 13.1, 3.9 Hz, 1H), 1.11 (s, 3H), 1.05 (s, 3H); ^13^C NMR (125 MHz, CDCl_3_) δ 222.3, 171.5, 153.4, 139.2, 128.4, 127.4, 127.3, 113.4, 74.2, 72.6, 69.9, 50.9, 42.3, 41.5, 39.0, 35.7, 33.4, 32.1, 30.5, 27.4, 25.6, 22.3, 21.4, 21.3, 20.2, 18.4; HR-ESI-MS (*m*/*z*) calcd for C_28_H_36_O_4_Na [M+Na]^+^: 459.2506, found: 459.2512.

#### 3.2.7. Synthesis of Compound **4α** and **4β**

To the solution of **13** (5.0 g, 11.5 mmol, 1.0 equiv.) in EtOH (230 mL), PPh_3_ (6.03 g, 23.0 mmol, 2.0 equiv.), and Mn(acac)_2_ (1.46 g, 5.75 mmol, 0.5 equiv.) were added. The reaction mixture was bubbled with O_2_ for 40 min. Next, to the solution, PhSiH_3_ (4.25 mL, 34.5 mmol, 3.0 equiv.) was added. The resulting mixture was stirred for 3 h at room temperature under an O_2_ atmosphere. TLC indicated the reaction was complete (petroleum ether/acetone = 4/1). The reaction mixture was quenched with a saturated Na_2_S_2_O_3_ solution (25 mL) at 0 °C. H_2_O (400 mL) was carefully added to the reaction mixture at room temperature. The resulting mixture was extracted with EtOAc (400 mL × 2); the combined organic phase was washed with brine, and dried with Na_2_SO_4_. After filtration, the solution was concentrated under reduced pressure. The residue was purified by silica gel column chromatography (petroleum ether/acetone = 8/1) to give **4β** (3.6 g, 68%) and **4α** (1.7 g, 32%) as white amorphous solids.

**4β**: [α${]}_{D}^{25}$ = −4.7 (*c* 1.0, CHCl_3_); ^1^H NMR (500 MHz, CDCl_3_) δ 7.35–7.30 (m, 4H), 7.26–7.23 (m, 1H), 4.95 (t, *J* = 3.1 Hz, 1H), 4.48–4.41 (m, 2H), 3.79–3.74 (m, 1H), 2.44–2.39 (m, 2H), 2.30–2.24 (m, 1H), 2.23–2.10 (m, 3H), 1.94 (s, 2H), 1.93–1.80 (m, 3H), 1.78–1.66 (m, 3H), 1.64–1.50 (m, 4H), 1.44–1.34 (m, 3H), 1.30–1.21 (m, 2H), 1.05 (s, 2H), 1.00 (s, 3H); ^13^C NMR (125 MHz, CDCl_3_) δ 221.0, 171.5, 139.1, 128.4, 127.4, 127.3, 82.5, 74.2, 72.4, 70.0, 41.6, 41.5, 38.8, 37.4, 33.1, 32.0, 31.7, 30.5, 27.5, 27.5, 25.8, 21.4, 20.4, 18.9, 18.5, 13.0; HR-ESI-MS (*m*/*z*) calcd for C_28_H_42_O_5_N [M+NH_4_]^+^: 472.3057, found: 472.3050.

**4α**: [α${]}_{D}^{25}$ = +11.2 (*c* 1.0, CHCl_3_); ^1^H NMR (600 MHz, CDCl_3_) δ 7.32–7.30 (m, 3H), 7.27–7.22 (m, 2H), 4.98–4.94 (m, 1H), 4.51–4.34 (m, 2H), 3.77–3.73 (m, 1H), 2.41 (ddd, *J* = 18.7, 9.5, 2.2 Hz, 1H), 2.33 (dt, *J* = 18.6, 8.6 Hz, 1H), 2.29–2.22 (m, 1H), 2.22–2.17 (m, 1H), 2.02–1.94 (m, 2H), 1.94 (s, 3H), 1.92–1.82 (m, 4H), 1.76 (td, *J* = 13.4, 4.5 Hz, 1H), 1.68–1.62 (m, 2H), 1.60–1.49 (m, 2H), 1.48–1.26 (m, 4H), 1.03 (s, 3H), 0.99 (s, 3H); ^13^C NMR (150 MHz, CDCl_3_) δ 218.7, 171.5, 139.2, 128.4, 127.4, 127.3, 81.4, 74.6, 72.6, 69.9, 52.6, 38.8, 38.2, 34.6, 33.1, 32.0, 30.5, 30.5, 27.2, 25.6, 25.1, 21.4, 19.5, 19.3, 18.5, 18.2; HR-ESI-MS (*m*/*z*) calcd for C_28_H_38_O_5_Na [M+Na]^+^: 477.2611, found: 477.2612.

#### 3.2.8. Synthesis of Compound **14**

To a stirring solution of **4β** (4.9 g, 10.7 mmol, 1.0 equiv.) in EtOH (110 mL), Et_3_N (4.5 mL, 32.1 mmol, 3.0 equiv.) and H_2_NNH_2_·H_2_O (1.6 mL, 32.1 mmol, 3.0 equiv.) were added. The mixture was stirred at 65 °C for 5 h. TLC indicated the reaction was complete (CH_2_Cl_2_/MeOH = 20/1). The reaction mixture was concentrated under reduced pressure. The residue was purified by silica gel column chromatography (CH_2_Cl_2_/MeOH = 60/1 → 20/1) to give intermediate hydrazone (4.4 g, 88%) as a white amorphous solid.

To the intermediate hydrazone (4.4 g, 9.5 mmol, 1.0 equiv.) in dry THF (100 mL), Et_3_N (13.1 mL, 94.5 mmol, 10.0 equiv.) and a solution of I_2_ (6.0 g, 23.6 mmol, 2.5 equiv.) in THF (100 mL) were added successively. The resulting mixture was stirred at 25 °C for 30 min under an Ar atmosphere. TLC indicated the reaction was complete (CH_2_Cl_2_/MeOH = 20/1). The reaction mixture was quenched with a saturated NaHCO_3_ solution (100 mL) and a Na_2_SO_3_ solution (100 mL) at 0 °C, and EtOAc (200 mL) was added. The mixture was extracted with EtOAc (100 mL × 2); the combined organic phase was washed with brine, and dried with Na_2_SO_4_. After filtration, the solution was concentrated under reduced pressure. The residue was purified by silica gel column chromatography (petroleum ether/EtOAc = 8/1) to give **14** (4.9 g, 91%) as a white foamy solid. [α${]}_{D}^{25}$ = −9.54 (*c* 1.0, CHCl_3_); ^1^H NMR (500 MHz, CDCl_3_) ^1^H NMR (500 MHz, CDCl_3_) δ 7.34–7.29 (m, 4H), 7.26–7.22 (m, 1H), 6.11 (dd, *J* = 3.2, 1.8 Hz, 1H), 5.00–4.96 (m, 1H), 4.49–4.39 (m, 2H), 3.78–3.72 (m, 1H), 2.54 (dd, *J* = 16.5, 1.9 Hz, 1H), 2.25 (ddd, *J* = 16.5, 7.1, 3.0 Hz, 2H), 2.18 (dd, *J* = 13.4, 3.6 Hz, 1H), 1.95 (s, 3H), 1.91 (dd, *J* = 13.8, 3.6 Hz, 1H), 1.88–1.70 (m, 4H), 1.67–1.59 (m, 2H), 1.51–1.39 (m, 2H), 1.38–1.24 (m, 2H), 1.14–1.07 (m, 1H)), 1.05 (s, 3H), 0.99 (s, 3H), 0.97–0.90 (m, 1H); ^13^C NMR (125 MHz, CDCl_3_) δ 171.5, 139.2, 133.7, 128.4, 127.4, 127.3, 111.4, 82.4, 74.3, 72.5, 69.9, 54.8, 42.8, 41.3, 38.8, 37.9, 37.5, 31.7, 30.5, 27.6, 25.8, 21.5, 20.4, 20.2, 18.7, 18.1; HR-ESI-MS (*m*/*z*) calcd for C_28_H_37_O_4_INa [M+Na]^+^: 587.1629, found: 587.1633.

#### 3.2.9. Synthesis of Compound **16**

A mixture of **14** (4.0 g, 7.1 mmol, 1.0 equiv.) and **16** (8.0 g, 21.3 mmol, 3.0 equiv.) was co-evaporated with toluene twice and recharged with Ar. CuCl (10.5 g, 106.0 mmol, 15.0 equiv.), LiCl (6.02 g, 142 mmol, 20.0 equiv.), and Pd(Ph_3_P)_4_ (819 mg, 0.709 mmol, 0.1 equiv.) were added. The resulting mixture was recharged with Ar and dry DMSO (150 mL) was added. The mixture was stirred for 2 h at 70 °C under an Ar atmosphere after Freeze–Pump–Thaw Degassing was performed twice. TLC indicated the reaction was complete (PE/EA = 2/1). The reaction mixture was quenched with a PBS (350 mL) at 0 °C. The mixture was filtered. The filter cake was washed with EtOAc (200 mL × 5). The aqueous phase was extracted with EtOAc (300 mL × 2); the combined organic phase was washed with a saturated NH_4_Cl solution (800 mL × 2) and brine, and dried with Na_2_SO_4_. After filtration, the solution was concentrated under reduced pressure. The residue was purified by silica gel column chromatography (containing 10% K_2_CO_3_, and petroleum ether/EtOAc/CH_2_Cl_2_ = 2/1/0.2) to give compound **16** (3.3 g, 89%) as a pale yellow amorphous solid. [α${]}_{D}^{25}$ = +37.5 (*c* 1.0, CHCl_3_); ^1^H NMR (400 MHz, CDCl_3_) δ 7.36–7.29 (m, 4H), 7.24 (d, *J* = 6.1 Hz, 1H), 6.10 (d, *J* = 2.8 Hz, 1H), 5.96 (s, 1H), 5.02–4.89 (m, 3H), 4.49–4.39 (m, 2H), 3.75 (s, 1H), 2.73–2.64 (m, 1H), 2.39 (dd, *J* = 18.4, 3.4 Hz, 1H), 2.26 (dd, *J* = 15.9, 2.8 Hz, 1H), 2.20 (d, *J* = 13.3 Hz, 1H), 2.06–1.98 (m, 1H), 1.95 (s, 3H), 1.91–1.72 (m, 3H), 1.69–1.59 (m, 3H), 1.51–1.33 (m, 4H), 1.28 (s, 3H), 1.18–1.05 (m, 1H), 1.01 (s, 3H), 0.95–0.88 (m, 1H); ^13^C NMR (100 MHz, CDCl_3_) δ 174.5, 171.5, 158.3, 144.0, 139.1, 132.2, 128.4, 127.4, 127.2, 112.6, 85.5, 74.2, 72.4, 71.8, 69.9, 52.2, 41.0, 40.6, 38.7, 38.4, 37.8, 31.6, 30.5, 27.6, 25.8, 21.4, 20.4, 20.3, 18.7, 16.7; HR-ESI-MS (*m*/*z*) calcd for C_32_H_40_O_6_Na [M+Na]^+^: 543.2717, found: 543.2715.

#### 3.2.10. Synthesis of Compound **17**

To a mixture of **16** (2.0 g, 3.8 mmol, 1.0 equiv.) and imidazole (1.6 g, 23.0 mmol, 6.0 equiv.), dry DMF (40 mL) was added under an atmosphere of Ar. TMSCl (1.5 mL, 11.5 mmol, 3.0 equiv.) was added to the resulting mixture under an Ar atmosphere and stirred for 12 h at 50 °C. TLC indicated the reaction was complete (petroleum ether/EtOAc = 1/1.5). The reaction mixture was quenched with MeOH (5 mL) at 0 °C. The mixture was extracted with CH_2_Cl_2_ (200 mL), washed with brine (200 mL × 3), and dried with Na_2_SO_4_. After filtration, the solution was concentrated under reduced pressure. The residue was purified by silica gel column chromatography (petroleum ether/EtOAc = 3/1) to give **17** (1.9 g, 83%) as a white foamy solid. [α${]}_{D}^{25}$ = +39.6 (*c* 1.0, CHCl_3_); ^1^H NMR (400 MHz, CDCl_3_) δ 7.34–7.26 (m, 5H), 6.03 (d, *J* = 2.8 Hz, 1H), 5.96 (s, 1H), 5.03–4.86 (m, 3H), 4.49–4.39 (m, 2H), 3.74 (d, *J* = 3.9 Hz, 1H), 2.59 (d, *J* = 18.5 Hz, 1H), 2.44 (dd, *J* = 18.6, 3.4 Hz, 1H), 2.25 (dd, *J* = 15.8, 2.8 Hz, 1H), 2.19 (d, *J* = 14.2 Hz, 1H), 1.98 (d, *J* = 3.6 Hz, 1H), 1.94 (s, 3H), 1.92–1.79 (m, 2H), 1.78–1.68 (m, 2H), 1.67–1.55 (m, 3H), 1.42–1.34 (m, 2H), 1.28–1.23 (m, 1H), 1.20 (s, 3H), 1.17–1.11 (m, 1H), 1.05 (d, *J* = 13.7 Hz, 1H), 1.00 (s, 3H), 0.00 (s, 9H); ^13^C NMR (100 MHz, CDCl_3_) δ 174.5, 171.5, 158.4, 145.0, 139.2, 132.0, 128.4, 127.4, 127.3, 112.6, 89.8, 74.3, 72.5, 71.7, 69.9, 52.9, 42.0, 39.4, 38.7, 38.7, 37.8, 31.7, 30.5, 27.6, 26.0, 21.5, 20.7, 20.3, 18.8, 17.4, 2.8; HR-ESI-MS (*m*/*z*) calcd for C_35_H_48_O_6_SiNa [M+Na]^+^: 615.3112, found: 615.3120.

#### 3.2.11. Synthesis of Compound **18**

To a solution of **17** (1.9 g, 3.2 mmol, 1.0 equiv.) in a mixture of EtOAc/MeOH/PBS (60 mL/30 mL/60 mL), Pd/C (1.0 g) was added. The reaction mixture was recharged with H_2_ thrice, and then stirred for 3 h at 0 °C under an atmosphere of H_2_. TLC indicated the reaction was complete (petroleum ether/EtOAc = 2/1). The reaction mixture was filtered, and the filter cake was washed with EtOAc (100 mL × 3). The filtrate was concentrated under reduced pressure. The resulting residue was purified by silica gel column chromatography (petroleum ether/EtOAc = 2/1 → 1/1.5) to give **18** (1.3 g, 68%) as a colorless amorphous solid. [α${]}_{D}^{25}$ = −4.35 (*c* 1.0, CHCl_3_); ^1^H NMR (500 MHz, CDCl_3_) δ 7.34–7.29 (m, 4H), 7.26–7.24 (m, 1H), 5.84 (t, *J* = 1.5 Hz, 1H), 4.95–4.90 (m, 1H), 4.79–4.67 (m, 2H), 4.49–4.39 (m, 2H), 3.76 (t, *J* = 3.1 Hz, 1H), 2.56 (t, *J* = 7.6 Hz, 1H), 2.30–2.22 (m, 1H), 2.17 (d, *J* = 13.4 Hz, 1H), 2.10–2.02 (m, 1H), 1.98–1.95 (m, 1H), 1.94 (s, 3H), 1.93–1.85 (m, 2H), 1.84–1.69 (m, 4H), 1.67–1.55 (m, 3H), 1.50 (td, *J* = 11.8, 3.5 Hz, 1H), 1.43–1.33 (m, 3H), 1.32–1.18 (m, 2H), 0.97 (s, 3H), 0.88 (s, 3H), 0.13 (s, 9H); ^13^C NMR (125 MHz, CDCl_3_) δ 174.4, 173.9, 171.5, 139.1, 128.4, 127.4, 127.3, 117.2, 91.3, 74.3, 74.0, 72.5, 70.0, 50.9, 50.6, 41.5, 40.9, 39.2, 38.2, 34.1, 31.7, 30.7, 27.4(2C), 26.1, 22.5, 21.5, 21.2, 18.4, 18.3, 3.1; HR-ESI-MS (*m*/*z*) calcd for C_35_H_50_O_6_SiNa [M+Na]^+^: 617.3269, found: 617.3275.

#### 3.2.12. Synthesis of Compound **3**

To a solution of **18** (1.5 g, 2.6 mmol, 1.0 equiv.) in a mixture of CHCl_3_/MeOH (60 mL/60 mL, Pd/C (0.8 g) was added. The reaction mixture was recharged with H_2_ thrice, and then stirred for 2 h at room temperature under an atmosphere of H_2_. TLC indicated the reaction was complete (petroleum ether/EtOAc = 1/1.5). The reaction mixture was filtered. The filtrate was concentrated under reduced pressure. The resulting residue was purified by silica gel column chromatography (CH_2_Cl_2_/MeOH = 30/1) to give **3** (869 mg, 78%) as a colorless amorphous solid. [α${]}_{D}^{25}$ = +9.4 (*c* 1.0, CHCl_3_); ^1^H NMR (500 MHz, CDCl_3_) δ 5.88 (s, 1H), 5.26 (d, *J* = 2.9 Hz, 1H), 4.97 (d, *J* = 18.0 Hz, 1H), 4.80 (d, *J* = 17.6 Hz, 1H), 4.04 (s, 1H), 2.80–2.75 (m, 1H), 2.23–2.11 (m, 1H), 2.09 (s, 3H), 2.06–1.79 (m, 6H), 1.77–1.68 (m, 2H), 1.66–1.59 (m, 2H), 1.58–1.49 (m, 2H), 1.47–1.26 (m, 6H), 0.96 (s, 3H), 0.87 (s, 3H); ^13^C NMR (125 MHz, CDCl_3_) δ 174.6, 174.4, 169.9, 118.0, 85.4, 75.8, 73.6, 66.9, 50.8, 49.5, 41.8, 39.8, 39.1, 37.1, 33.3(2 C), 31.5, 31.1, 27.0, 25.8, 21.6, 21.4, 20.8, 18.5, 15.9; HR-ESI-MS (*m*/*z*) calcd for C_25_H_36_O_6_Na [M+Na]^+^: 455.2404, found: 455.2411.

#### 3.2.13. Synthesis of Compound **20**

To a stirring solution of **19** (10.0 mg, 0.027 mmol, 1.0 equiv.) in dry CH_2_Cl_2_ (7 mL), NCCCl_3_ (11 μL, 0.108 mmol, 4.0 equiv.) and Cs_2_CO_3_ (0.87 mg, 2.69 μmol, 0.1 equiv.) were added. The resulting mixture was stirred for 3 h at room temperature. TLC indicated the reaction was complete. The reaction mixture was filtered. The filtrate was concentrated in vacuo. The residue and aglycone **3** (9.7 mg, 0.022 mmol, 1.0 equiv.) were co-evaporated with toluene three times and dissolved in dry CH_2_Cl_2_ (1.5 mL); a 4Å molecular sieve (150 mg) was added. The mixture was stirred for 15 min at 25 °C under an Ar atmosphere. Next, TMSOTf (0.81 μL, 4.48 μmol, 0.2 equiv.) was added at −78 °C. The resulting mixture was stirred for 10 min at −78 °C. TLC indicated the reaction was complete (petroleum ether/EtOAc = 1/2). The reaction mixture was quenched with a saturated NaHCO_3_ aqueous solution (2 mL) and filtered. The filtrate was added to the saturated NaHCO_3_ solution (20 mL). The mixture was extracted with CH_2_Cl_2_ (20 mL × 3), washed with brine, and dried with Na_2_SO_4_. After filtration, the solution was concentrated under reduced pressure. The residue was purified by silica gel column chromatography (petroleum ether/EtOAc = 4/1) to give compound **20** (8.9 mg, 50%) as a white amorphous solid. [α${]}_{D}^{25}$ = −34.5 (*c* 1.0, CHCl_3_); ^1^H NMR (600 MHz, CDCl_3_) δ 8.01–7.98 (m, 2H), 7.46–7.38 (m, 3H), 7.34–7.27 (m, 3H), 7.17–7.11 (m, 2H), 5.87 (t, *J* = 1.9 Hz, 1H), 5.43–5.34 (m, 1H), 5.00–4.94 (m, 3H), 4.91 (t, *J* = 3.0 Hz, 1H), 4.80 (dd, *J* = 18.1, 1.8 Hz, 1H), 4.62 (d, *J* = 11.6 Hz, 1H), 4.04 (s, 1H), 3.95–3.90 (m, 1H), 3.68–3.63 (m, 2H), 3.43 (s, 3H), 2.77 (dd, *J* = 9.5, 5.5 Hz, 1H), 2.19–2.10 (m, 2H), 2.08 (s, 3H), 2.07–1.99 (m, 2H), 1.91–1.76 (m, 4H), 1.74–1.65 (m, 3H), 1.64–1.57 (m, 3H), 1.57–1.50 (m, 2H), 1.48–1.42 (m, 1H), 1.39 (d, *J* = 15.3 Hz, 1H), 1.35 (d, *J* = 6.5 Hz, 3H), 1.30 (dt, *J* = 13.1, 3.4 Hz, 1H), 0.99 (s, 3H), 0.88 (s, 3H); δ ^13^C NMR (150 MHz, CDCl_3_) δ 174.5, 174.3, 171.0, 166.7, 139.3, 133.0, 130.4, 129.9, 128.3, 128.3, 128.0, 127.4, 118.0, 95.7, 85.5, 77.9, 76.4, 74.7, 74.3, 73.5, 69.3, 67.9, 67.3, 57.1, 50.9, 49.6, 41.7, 39.9, 38.8, 37.0, 33.3, 31.2, 30.0, 29.8, 28.0, 27.0, 25.8, 21.7, 20.7, 18.6, 17.1, 15.9; HR-ESI-MS (*m*/*z*) calcd for C_46_H_58_O_11_Na [M+Na]^+^: 809.3871, found: 809.3875.

#### 3.2.14. Synthesis of Compound **2**

To a solution of **20** (8.9 mg, 11.3 μmol, 1.0 equiv.) in EtOAc/MeOH (1.0 mL/1.0 mL), 10% Pd/C (8.9 mg) was added. The reaction mixture was recharged with H_2_ 3 times, and then stirred for 18 h at 25 °C under an atmosphere of H_2_. TLC indicated the reaction was complete (petroleum ether/EtOAc = 1/2). The reaction mixture was filtered. The filtrate was concentrated under reduced pressure. The residue was dissolved in MeOH/H_2_O/THF (1.5 mL/0.5 mL/0.5 mL); LiOH (2.0 mg, 83.5 μmol, 7.4 equiv.) was added and then the mixture was stirred for 0.5 h at 25 °C. TLC indicated the reaction was complete (petroleum ether/EtOAc = 1/2). The reaction mixture was quenched with HOAc (4.8 μL, 83.5 μmol, 7.4 equiv.) and concentrated in vacuo. The residue was purified by reverse-phase columns (H_2_O/MeOH = 1/2) to afford acovensoide B (**2**) (4.0 mg, 60% over 2 steps) as a white amorphous solid. [α${]}_{D}^{25}$ = −53.0 (*c* 0.4, CHCl_3_); ^1^H NMR (400 MHz, CDCl_3_) δ 5.88 (s, 1H), 5.01–4.93 (m, 2H), 4.88 (t, *J* = 3.2 Hz, 1H), 4.80 (d, *J* = 18.1 Hz, 1H), 4.04 (s, 1H), 3.86 (s, 1H), 3.84–3.76 (m, 2H), 3.47 (s, 3H), 3.34 (t, *J* = 3.2 Hz, 1H), 2.78 (dd, *J* = 9.3, 5.6 Hz, 1H), 2.22–2.03 (m, 3H), 2.01 (s, 3H), 1.97 (s, 1H), 1.88–1.68 (m, 7H), 1.58–1.49 (m, 4H), 1.46–1.39 (m, 1H), 1.38–1.32 (m, 2H), 1.29 (d, *J* = 6.5 Hz, 3H), 1.25–1.22 (m, 1H), 0.97 (s, 3H), 0.87 (s, 3H); ^13^C NMR (150 MHz, CDCl_3_) δ 174.5, 174.3, 170.8, 117.9, 97.4, 85.4, 75.4, 74.2, 73.5, 70.1, 68.7, 68.6, 66.2, 55.6, 50.8, 49.5, 41.7, 39.8, 38.8, 36.9, 33.2, 31.1, 29.9, 27.8, 26.9, 25.8, 21.6, 21.5, 20.6, 18.5, 16.6, 15.8; HR-ESI-MS (*m*/*z*) calcd for C_32_H_48_O_10_Na [M+Na]^+^: 615.3140, found: 615.3142.

#### 3.2.15. Synthesis of Compound **22**

To a stirring solution of **21** (200 mg, 0.418 mmol, 1.0 equiv.) in a mixture of CH_2_Cl_2_/H_2_O (4 mL/0.4 mL), DDQ (285 mg, 1.3 mmol, 3.0 equiv.) was added. The resulting mixture was stirred for 12 h at room temperature. TLC indicated the reaction was complete (petroleum ether/EtOAc = 4/1). The reaction mixture was quenched with a saturated NaHCO_3_ solution (20 mL). The mixture was extracted with CH_2_Cl_2_ (20 mL × 3), washed with brine, and dried with Na_2_SO_4_. After filtration, the solution was concentrated under reduced pressure. The residue was purified by silica gel column chromatography (petroleum ether/EtOAc = 4/1) to give compound **22** (146 mg, 90%) as a pale yellow amorphous solid. [α${]}_{D}^{25}$ = −106.8 (*c* 1.0, CHCl_3_); ^1^H NMR (500 MHz, CDCl_3_) δ 8.02–7.99 (m, 2H), 7.59–7.54 (m, 1H), 7.44 (t, *J* = 7.8 Hz, 2H), 7.40–7.36 (m, 2H), 7.16–7.10 (m, 2H), 5.70 (dt, *J* = 3.5, 1.3 Hz, 1H), 5.56 (d, *J* = 1.4 Hz, 1H), 4.50–4.45 (m, 1H), 3.92 (ddt, *J* = 7.9, 3.1, 1.3 Hz, 1H), 3.66 (t, *J* = 3.5 Hz, 1H), 3.48 (s, 3H), 2.55 (d, *J* = 7.9 Hz, 1H), 2.33 (s, 3H), 1.40 (d, *J* = 6.5 Hz, 3H); ^13^C NMR (125 MHz, CDCl_3_) δ 165.6, 138.2, 133.6, 132.5, 130.1, 129.9, 129.8, 129.5, 128.7, 87.0, 75.2, 70.2, 69.6, 68.2, 56.5, 21.3, 16.5; HR-ESI-MS (*m*/*z*) calcd for C_21_H_24_O_5_SNa [M+Na]^+^: 411.1237, found: 411.1242.

#### 3.2.16. Synthesis of Compound **24**

To a stirring solution of 2-*O*-benzoyl-3,4,6-tri-*O*-benzyl-d-glucopyranose (223 mg, 0.402 mmol, 1.0 equiv.) in dry CH_2_Cl_2_ (4 mL), NCCCl_3_ (0.164 mL, 1.6 mmol, 4.0 equiv.) and Cs_2_CO_3_ (13.1 mg, 40.2 μmol, 0.1 equiv.) were added. The resulting mixture was stirred for 4 h at room temperature. TLC indicated the reaction was complete (petroleum ether/EtOAc = 4/1). The reaction mixture was filtered. The filtrate was concentrated in vacuo.

The residue and acceptor **22** (130 mg, 0.335 mmol, 1.0 equiv.) were co-evaporated with toluene thrice and dissolved in dry CH_2_Cl_2_ (3.5 mL); a 4 Å molecular sieve (350 mg) was added. The mixture was stirred for 15 min at 25 °C under an Ar atmosphere. Next, TMSOTf (0.81 μL, 4.48 μmol, 0.2 equiv.) was added at −78 °C. The resulting mixture was stirred for 2 h at −78 °C. TLC indicated the reaction was complete (petroleum ether/EtOAc = 3/1). The reaction mixture was quenched with a saturated NaHCO_3_ solution (2 mL) and filtered. The filtrate was added to the saturated NaHCO_3_ solution (20 mL). The mixture was extracted with CH_2_Cl_2_ (20 mL × 3), washed with brine, and dried with Na_2_SO_4_. After filtration, the solution was concentrated under reduced pressure. The residue was purified by silica gel column chromatography (petroleum ether/EtOAc = 6/1) to give compound **24** (234 mg, 76%) as a pale yellow foamy solid. [α${]}_{D}^{25}$ = −57.0 (*c* 1.0, CHCl_3_); ^1^H NMR (500 MHz, CDCl_3_) δ 8.31–8.25 (m, 2H), 8.17–8.11 (m, 2H), 7.75–7.65 (m, 4H), 7.55 (t, *J* = 7.8 Hz, 2H), 7.49–7.37 (m, 11H), 7.34 (dd, *J* = 7.3, 2.1 Hz, 2H), 7.26 (d, *J* = 3.0 Hz, 4H), 7.18 (d, *J* = 7.9 Hz, 2H), 5.58 (dd, *J* = 9.4, 8.0 Hz, 1H), 5.49–5.42 (m, 2H), 4.95 (d, *J* = 10.9 Hz, 1H), 4.88 (d, *J* = 11.1 Hz, 1H), 4.78 (d, *J* = 9.2 Hz, 1H), 4.76 (d, *J* = 6.0 Hz, 1H), 4.71 (d, *J* = 11.0 Hz, 1H), 4.62 (s, 2H), 4.42–4.36 (m, 1H), 4.09 (t, *J* = 3.4 Hz, 1H), 3.95 (t, *J* = 9.1 Hz, 1H), 3.91 (s, 1H), 3.86–3.76 (m, 3H), 3.74–3.69 (m, 1H), 3.58 (s, 3H), 2.42 (s, 3H), 1.38 (d, *J* = 6.7 Hz, 3H); ^13^C NMR (125 MHz, CDCl_3_) δ 166.2, 165.0, 138.1, 137.9(2), 137.9(0), 137.8, 133.2, 133.1, 132.7, 130.5, 130.1, 129.9(2), 129.8(6), 129.8, 129.5, 128.6(4), 128.5(9), 128.5(1), 128.4(8), 128.4, 128.3, 128.2, 128.0, 127.9, 127.8(0), 127.7(7), 102.2, 83.2, 78.2, 76.0, 75.6, 75.2, 75.1, 74.0, 73.7, 69.9, 69.2, 60.5, 58.8, 21.2, 15.4; HR-ESI-MS (*m*/*z*) calcd for C_55_H_56_O_11_SNa [M+Na]^+^: 947.3436, found: 947.3429.

#### 3.2.17. Synthesis of Compound **25**

To a stirring solution of **24** (60.0 mg, 64.9 μmol, 1.0 equiv.) in THF/MeOH (1 mL/1 mL), KOH (10.0 mg, 0.178 mmol, 2.7 equiv.) was added at room temperature. After, the reaction mixture was stirred for 12 h at the same temperature. TLC indicated the reaction was complete (petroleum ether/EtOAc = 2/1). The reaction mixture was quenched with HOAc (10.5 μL, 183 μmol, 2.7 equiv.). H_2_O (30 mL) was added to the mixture; the mixture was extracted with CH_2_Cl_2_ (30 mL × 3), washed with brine, and dried with Na_2_SO_4_. After filtration, the solution was concentrated under reduced pressure. The residue was purified by silica gel column chromatography (petroleum ether/EtOAc = 2/1) to give compound **25** (37.4 mg, 80%) as a white amorphous solid. [α${]}_{D}^{25}$ = −128.8 (*c* 1.0, CHCl_3_); ^1^H NMR (500 MHz, CDCl_3_) δ 7.41–7.27 (m, 15H), 7.22–7.19 (m, 2H), 7.13 (dt, *J* = 8.4, 1.3 Hz, 2H), 5.55 (d, *J* = 1.4 Hz, 1H), 5.03 (d, *J* = 11.2 Hz, 1H), 4.86 (d, *J* = 10.9 Hz, 1H), 4.83 (d, *J* = 11.2 Hz, 1H), 4.57 (d, *J* = 10.8 Hz, 1H), 4.53 (d, *J* = 12.0 Hz, 1H), 4.51–4.47 (m, 2H), 4.36 (q, *J* = 6.5 Hz, 1H), 4.17 (d, *J* = 6.4 Hz, 1H), 4.12 (dt, *J* = 2.7, 1.2 Hz, 1H), 4.03 (s, 1H), 3.71–3.66 (m, 2H), 3.65–3.55 (m, 3H), 3.52–3.49 (m, 1H), 3.52–3.49 (m, 1H), 3.51 (s, 3H), 2.34 (s, 3H), 1.31 (d, *J* = 6.5 Hz, 3H); ^13^C NMR (125 MHz, CDCl_3_) δ 138.9, 138.2, 138.2, 137.8, 132.1, 130.3, 130.0, 128.5, 128.5, 128.5, 128.1, 128.1, 127.9, 127.8, 127.8, 127.7, 102.0, 90.0, 84.4, 77.3, 76.0, 75.5, 75.4, 75.1, 73.8, 73.6, 73.5, 69.3, 69.2, 67.9, 56.3, 21.2, 17.3; HR-ESI-MS (*m*/*z*) calcd for C_41_H_48_O_9_SNa [M+Na]^+^: 739.2911, found: 739.2918.

#### 3.2.18. Synthesis of Compound **26**

To a stirring solution of compound **25** (37.4 mg, 52.2 μmol, 1.0 equiv.) in isopropenyl acetate (1 mL), TsOH·H_2_O (30 mg, 158 μmol, 3.0 equiv.) was added at room temperature. After, the reaction mixture was stirred for 1 h at the same temperature. TLC indicated the reaction was complete (petroleum ether/EtOAc = 2/1). CH_2_Cl_2_ (30 mL) was added, and the reaction mixture was quenched with a saturated NaHCO_3_ solution (30 mL) and extracted with CH_2_Cl_2_ (30 mL × 2). The organic phase was washed with brine, and dried with Na_2_SO_4_. After filtration, the solution was concentrated under reduced pressure. The residue was purified by silica gel column chromatography (petroleum ether/EtOAc = 4/1) to give compound **26** (37.3 mg, 89%) as a white amorphous solid. [α${]}_{D}^{25}$ = −49.0 (*c* 1.0, CHCl_3_); ^1^H NMR (500 MHz, CDCl_3_) δ 7.37–7.26 (m, 15H), 7.19 (dd, *J* = 7.3, 2.2 Hz, 2H), 7.11 (d, *J* = 7.9 Hz, 2H), 5.31 (d, *J* = 3.8 Hz, 1H), 5.15–5.10 (m, 1H), 5.10–5.06 (m, 1H), 4.79 (dd, *J* = 11.2, 2.4 Hz, 2H), 4.67 (d, *J* = 11.4 Hz, 1H), 4.57–4.48 (m, 3H), 4.42 (d, *J* = 7.9 Hz, 1H), 4.28 (qd, *J* = 6.7, 2.6 Hz, 1H), 3.91 (t, *J* = 3.0 Hz, 1H), 3.72 (dd, *J* = 10.6, 2.0 Hz, 1H), 3.68–3.62 (m, 3H), 3.61–3.57 (m, 1H), 3.53–3.47 (m, 1H), 3.44 (s, 3H), 2.32 (s, 3H), 2.14 (s, 3H), 1.95 (s, 3H), 1.31 (d, *J* = 6.7 Hz, 3H); ^13^C NMR (125 MHz, CDCl_3_) δ 171.2, 169.3, 138.3, 138.0, 138.0, 137.9, 132.7, 129.9, 129.7, 128.6, 128.5, 128.2, 128.0, 128.0, 127.9, 127.9, 127.8, 101.2, 83.3, 78.1, 76.6, 75.2, 75.1, 75.1, 74.4, 73.7, 73.5, 68.8, 58.1, 21.2(2C), 21.1, 15.9; HR-ESI-MS (*m*/*z*) calcd for C_45_H_52_O_11_SNa [M+Na]^+^: 823.3125, found: 823.3123.

#### 3.2.19. Synthesis of Compound **27**

To a stirring solution of **26** (37.3 mg, 0.047 mmol, 1.0 equiv.) in a mixture of acetone/H_2_O/CH_2_Cl_2_ (1 mL/0.1 mL/1 mL), NBS (24.9 mg, 0.140 mmol, 3.0 equiv.) was added at −20 °C. The resulting mixture was stirred for 0.5 h at the same temperature. TLC indicated the reaction was complete (petroleum ether/EtOAc = 3/1). The reaction mixture was quenched with NEt_3_ (0.5 mL). A saturated NaHCO_3_ solution (30 mL) was added. The mixture was extracted with CH_2_Cl_2_ (30 mL × 3), washed with brine, and dried with Na_2_SO_4_. After filtration, the solution was concentrated under reduced pressure. The residue was purified by silica gel column chromatography (petroleum ether/EtOAc = 2/1) to give compound **27** (30.0 mg, 93%) as a white foamy solid. The solid was used in the next step without further characterization.

#### 3.2.20. Synthesis of Compound **28**

To a stirring solution of **27** (30.0 mg, 0.043 mmol, 1.0 equiv.) in dry CH_2_Cl_2_ (2 mL), NCCCl_3_ (17.7 μL, 0.173 mmol, 4.0 equiv.) and Cs_2_CO_3_ (1.4 mg, 2.69 μmol, 0.1 equiv.) were added. The resulting mixture was stirred for 2 h at room temperature. TLC indicated the reaction was complete (petroleum ether/EtOAc = 1/2). The reaction mixture was filtered. The filtrate was concentrated in vacuo.

The residue and aglycone **3** (12.5 mg, 0.029 mmol, 1.5 equiv.) were co-evaporated with toluene three times and dissolved in dry CH_2_Cl_2_ (2.5 mL); a 4 Å molecular sieve (250 mg) was added. The mixture was stirred for 10 min at 25 °C under an Ar atmosphere. Next, TMSOTf (1.1 μL, 5.76 μmol, 0.2 equiv.) was added at −78 °C. The resulting mixture was stirred for 1 h at −78 °C. TLC indicated the reaction was complete (petroleum ether/EtOAc = 1/2). The reaction mixture was quenched with a saturated NaHCO_3_ solution (2 mL) and filtered. The filtrate was added to the saturated NaHCO_3_ solution (20 mL). The mixture was extracted with CH_2_Cl_2_ (20 mL × 3), and the organic phase was washed with brine, and dried with Na_2_SO_4_. After filtration, the solution was concentrated under reduced pressure. The residue was purified by silica gel column chromatography (petroleum ether/EtOAc = 1/1) to give compound **28** (16.4 mg, 51% over 2 steps) as a pale yellow foamy solid. [α${]}_{D}^{25}$ = −34.5 (*c* 1.0, CHCl_3_); ^1^H NMR (400 MHz, CDCl_3_) δ 7.34–7.27 (m, 13H), 7.20–7.15 (m, 2H), 5.87 (t, *J* = 1.8 Hz, 1H), 5.14–5.07 (m, 1H), 4.97 (dd, *J* = 18.3, 1.8 Hz, 1H), 4.91 (t, *J* = 3.0 Hz, 1H), 4.87 (t, *J* = 2.9 Hz, 1H), 4.83–4.74 (m, 4H), 4.66 (d, *J* = 11.4 Hz, 1H), 4.56–4.48 (m, 3H), 4.39 (d, *J* = 7.9 Hz, 1H), 3.95 (s, 1H), 3.86–3.83 (m, 1H), 3.81 (d, *J* = 6.7 Hz, 1H), 3.72–3.58 (m, 5H), 3.52–3.46 (m, 2H), 3.38 (s, 3H), 2.77 (dd, *J* = 9.5, 5.5 Hz, 1H), 2.17 (s, 1H), 2.13 (s, 3H), 2.11–2.06 (m, 2H), 2.01 (s, 3H), 1.98–1.96 (m, 3H), 1.94 (d, *J* = 1.4 Hz, 1H), 1.87–1.66 (m, 10H), 1.60–1.50 (m, 2H), 1.43–1.34 (m, 3H), 1.25 (d, *J* = 6.7 Hz, 3H), 0.96 (s, 3H), 0.87 (s, 3H); ^13^C NMR (150 MHz, CDCl_3_) δ 174.5, 174.3, 171.7, 171.1, 169.3, 138.3, 138.0, 137.9, 128.6, 128.6, 128.5, 128.3, 128.1, 128.0, 127.9, 127.9, 118.0, 101.4, 95.0, 85.5, 83.4, 78.2, 75.2, 75.1, 75.1, 74.3, 74.1, 73.6, 73.6, 73.5, 70.0, 69.4, 68.9, 67.1, 60.5, 57.5, 50.9, 49.6, 41.7, 39.9, 38.8, 37.0, 33.3, 31.1, 29.9, 28.5, 28.4, 27.0, 25.8, 21.7, 21.6, 21.3, 21.1, 20.6, 18.5, 16.5, 15.9; HR-ESI-MS (*m*/*z*) calcd for C_63_H_80_O_17_Na [M+Na]^+^: 1131.5288, found: 1131.5281.

#### 3.2.21. Synthesis of Compound **1**

To a solution of **28** (8.6 mg, 7.8 μmol, 1.0 equiv.) in EtOAc/MeOH (1.0 mL/1.0 mL), 10% Pd/C (8.6 mg) was added. The reaction mixture was recharged with H_2_ thrice, and then stirred for 5 h at 25 °C under an atmosphere of H_2_. TLC indicated the reaction was complete (CH_2_Cl_2_/MeOH = 20/1). The reaction mixture was filtered. The filtrate was concentrated under reduced pressure.

The residue was dissolved in MeOH/H_2_O/THF (1.5 mL/0.5 mL/0.5 mL); LiOH (2.0 mg, 83.5 μmol, 10.8 equiv.) was added and then the mixture was stirred for 2 h at 25 °C. TLC indicated the reaction was complete (CH_2_Cl_2_ / MeOH = 10/1). The reaction mixture was quenched with HOAc (4.8 μL, 83.5 μmol, 10.8 equiv.) and concentrated in vacuo. The residue was purified by reverse-phase columns (H_2_O/MeOH = 2/1 → 1/2) to afford acospectoside A (**1**) (2.5 mg, 43% over 2 steps) as a white amorphous solid. [α${]}_{D}^{25}$ = −27.0 (*c* 0.3, CH_3_OH); ^1^H NMR (400 MHz, C_5_D_5_N) δ 6.16 (d, *J* = 1.9 Hz, 1H), 5.50 (s, 1H), 5.35 (d, *J* = 18.4 Hz, 1H), 5.31 (d, *J* = 1.7 Hz, 1H), 5.18 (s, 1H), 5.09 (s, 1H), 4.57 (dd, *J* = 11.5, 2.6 Hz, 1H), 4.49 (d, *J* = 2.6 Hz, 1H), 4.44–4.37 (m, 1H), 4.29–4.18 (m, 3H), 4.14 (s, 1H), 4.10 (d, *J* = 6.6 Hz, 1H), 3.99 (q, *J* = 8.5, 7.2 Hz, 2H), 3.78 (t, *J* = 3.1 Hz, 1H), 3.70 (s, 3H), 2.86–2.76 (m, 1H), 2.29–2.22 (m, 1H), 2.21 (s, 3H), 2.17–2.06 (m, 4H), 2.02–1.89 (m, 3H), 1.87–1.79 (m, 2H), 1.74 (d, *J* = 6.6 Hz, 3H), 1.68 (d, *J* = 12.5 Hz, 1H), 1.64–1.54 (m, 1H), 1.47–1.19 (m, 8H), 1.04 (s, 3H), 1.00 (s, 3H); ^13^C NMR (150 MHz, C_5_D_5_N) δ 175.7, 174.4, 170.6, 117.6, 105.1, 99.0, 84.3, 78.4, 78.3, 76.5, 76.4, 75.3, 74.3, 73.5, 71.3, 69.8, 69.4, 67.3, 62.6, 55.9, 51.1, 49.8, 41.5, 39.4, 38.9, 36.7, 32.9, 31.4, 29.9, 28.1, 27.1, 26.1, 21.7, 21.3, 21.0, 18.3, 17.2, 16.0; HR-ESI-MS (*m*/*z*) calcd for C_38_H_58_O_15_Na [M+Na]^+^: 777.3668, found: 777.3676.

## 4. Conclusions

Here, we report the total synthesis of cytotoxic cardenolides acospectoside A (**1**) and acovensoide B (**2**), which were isolated from the South African poisonous bush *Acokanthera oppositifolia* and relevant plants. The synthesis features the selective introductions of a 15,16-unsaturated enone via the Larock protocol of Saegusa–Ito oxidation, 14β-OH by Mukaiyama hydration, and a C17-butenolide moiety by Stille coupling. Leveraging the strategic 1-*O*-acetylation of the aglycone moiety, glycosylation with imidate donors proceeds with remarkable selectivity, yielding glycosylated products in satisfactory amounts. Given the structural conservation inherent in the acovenoside family, the present synthetic approach should offer a promising gateway to accessing a broader array of the acovenoside congeners. This advancement stands to facilitate comprehensive investigations into the biological and pharmacological activities of these components, which have long been esteemed in traditional medicinal practices.

## Data Availability

The data presented in this study are available in the article and Supplementary Material.

## Supplementary Materials

The following supporting information can be downloaded at https://www.mdpi.com/article/10.3390/molecules30112297/s1: Copies of the NMR spectra of compounds **1**–**28**; Figure S1: ^1^H NMR spectrum of compound **7** (CDCl_3_, 500 MHz); and Table S1: Comparison of the Spectroscopic Data of Natural and Synthetic Acovenoside B (**2**).
