# Supplementary material for: Total Synthesis of Cardenolides Acospectoside A and Acovenoside B
Source: Molecules. 2025 May 23;30(11):2297. doi: 10.3390/molecules30112297 (PMC12156471; doi:10.3390/molecules30112297)
Supplement: Supplementary file 1 [file molecules-30-02297-s001.zip › molecules-3659279-supplementary-update I.pdf]

Total Synthesis of Cardenolides Acospectoside A and Acovenoside B

Benzhang Liu [1,2], Peng Xu [1,2,*] and Biao Yu [1,2,*]

1 School of Chemistry and Materials Science, Hangzhou Institute for Advanced Study, University of Chinese Academy of Sciences, 1 Sub-lane Xiangshan, Hangzhou 310024, China

2 State Key Laboratory of Chemical Biology, Shanghai Institute of Organic Chemistry, University of Chi-nese Academy of Sciences, Chinese Academy of Sciences, 345 Lingling Road, Shanghai 200032, China

* Correspondence: peterxu@sioc.ac.cn (P.X.); byu@sioc.ac.cn (B.Y.)

Table of Contents

Copies of ^{1}H NMR and ^{13}C NMR spectra of compounds **1-28**………S2

Comparison of ^{13}C NMR data of the synthetic **1-2** with those reported for the natural products…. …………………………………………………. ……………………………………S60

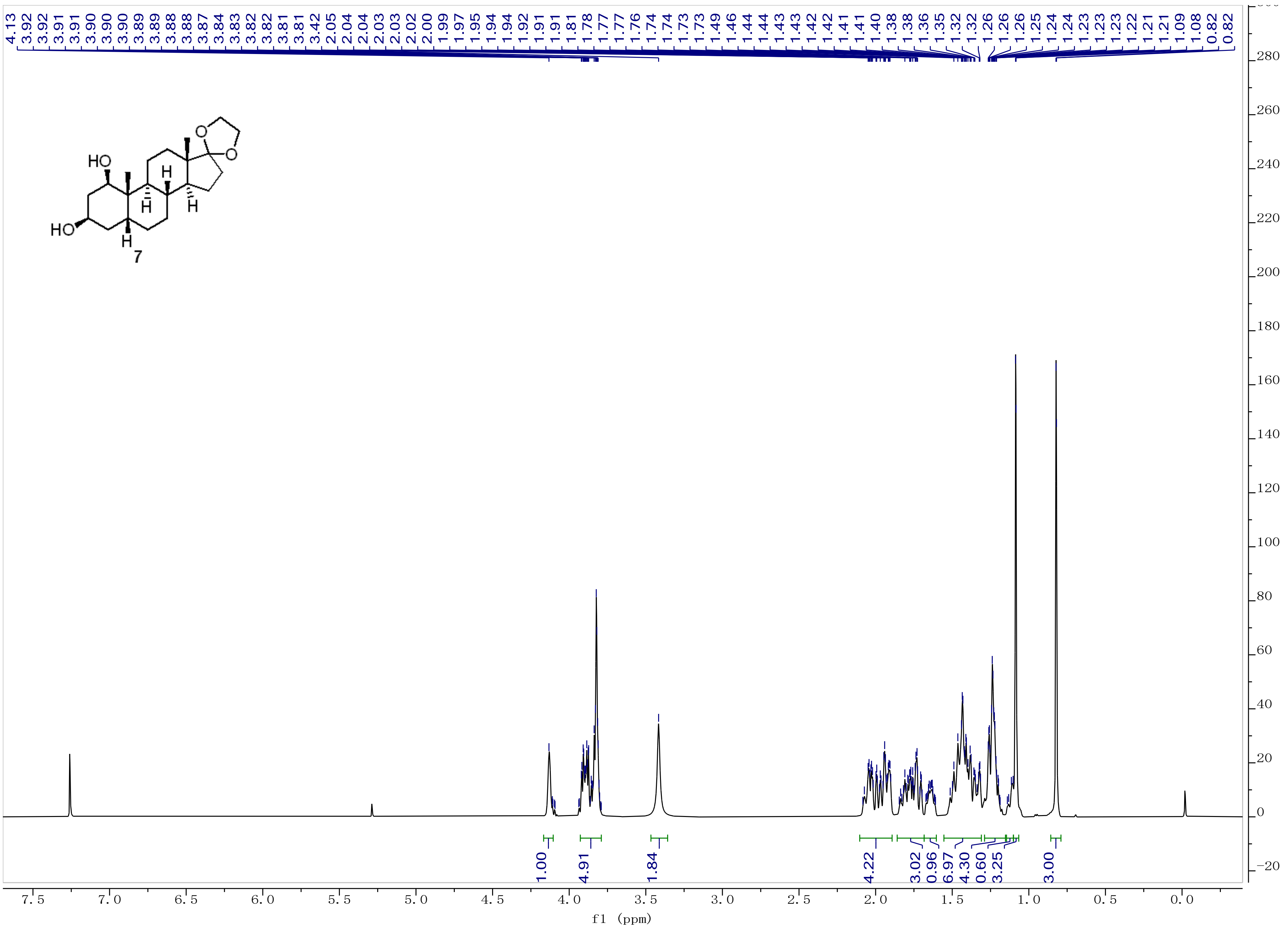

Figure S1 ^{1}H NMR spectrum of compound **7** ($CDCl_3$, 500 MHz)

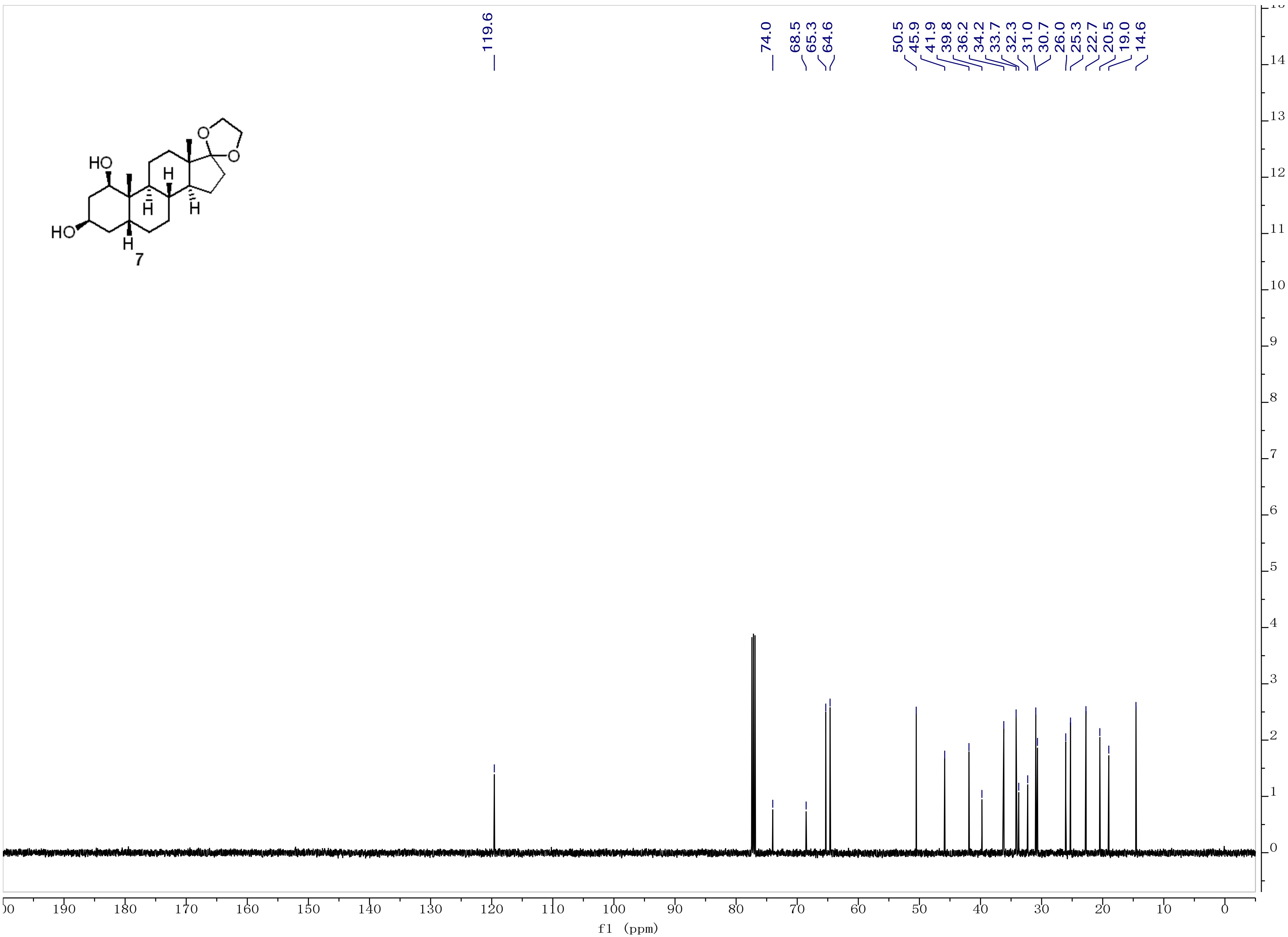

Figure S2 ^{13}C NMR spectrum of compound **7** ($CDCl_3$, 125 MHz)

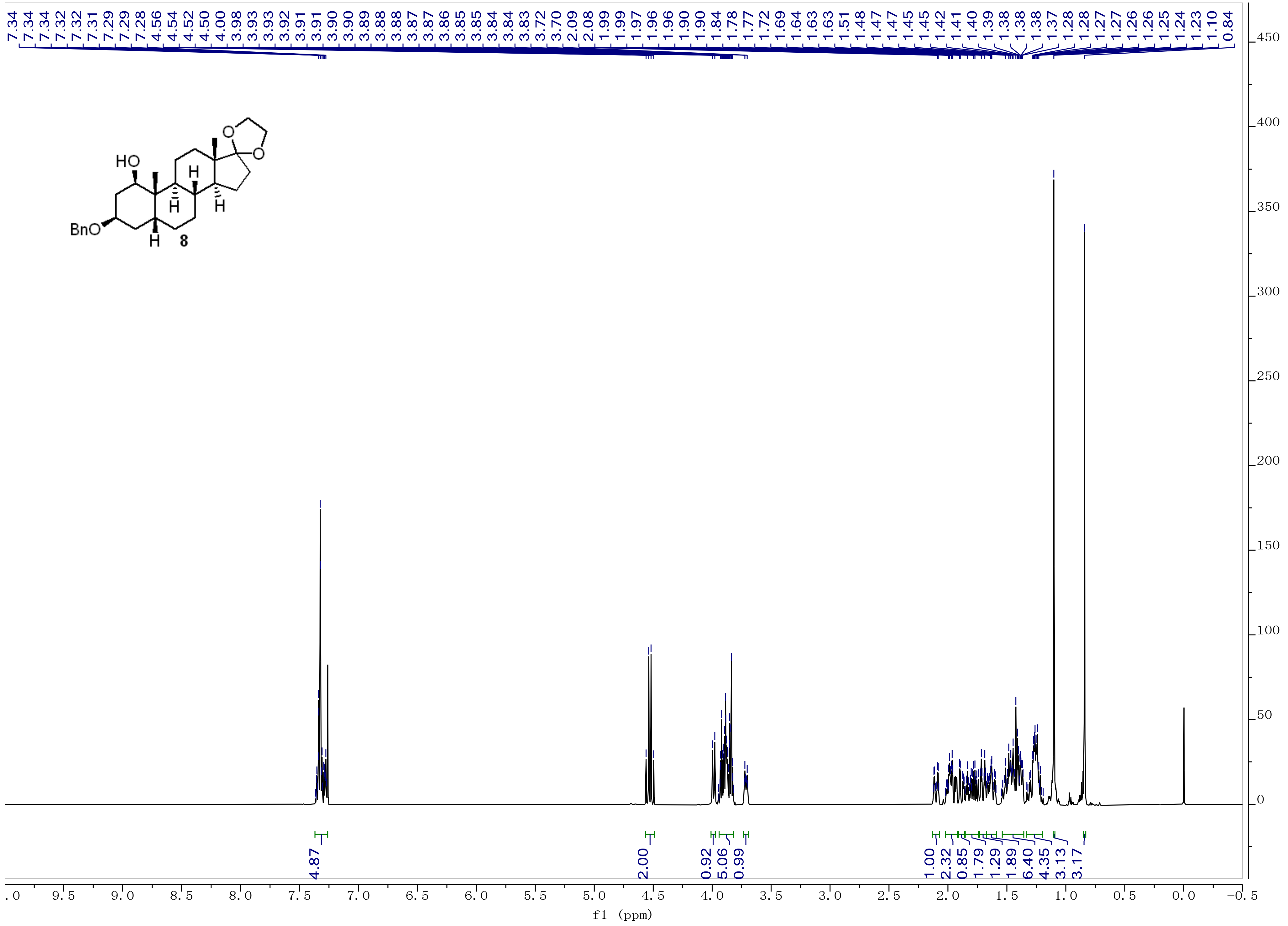

Figure S3 ^{1}H NMR spectrum of compound **8** ($CDCl_3$, 500 MHz)

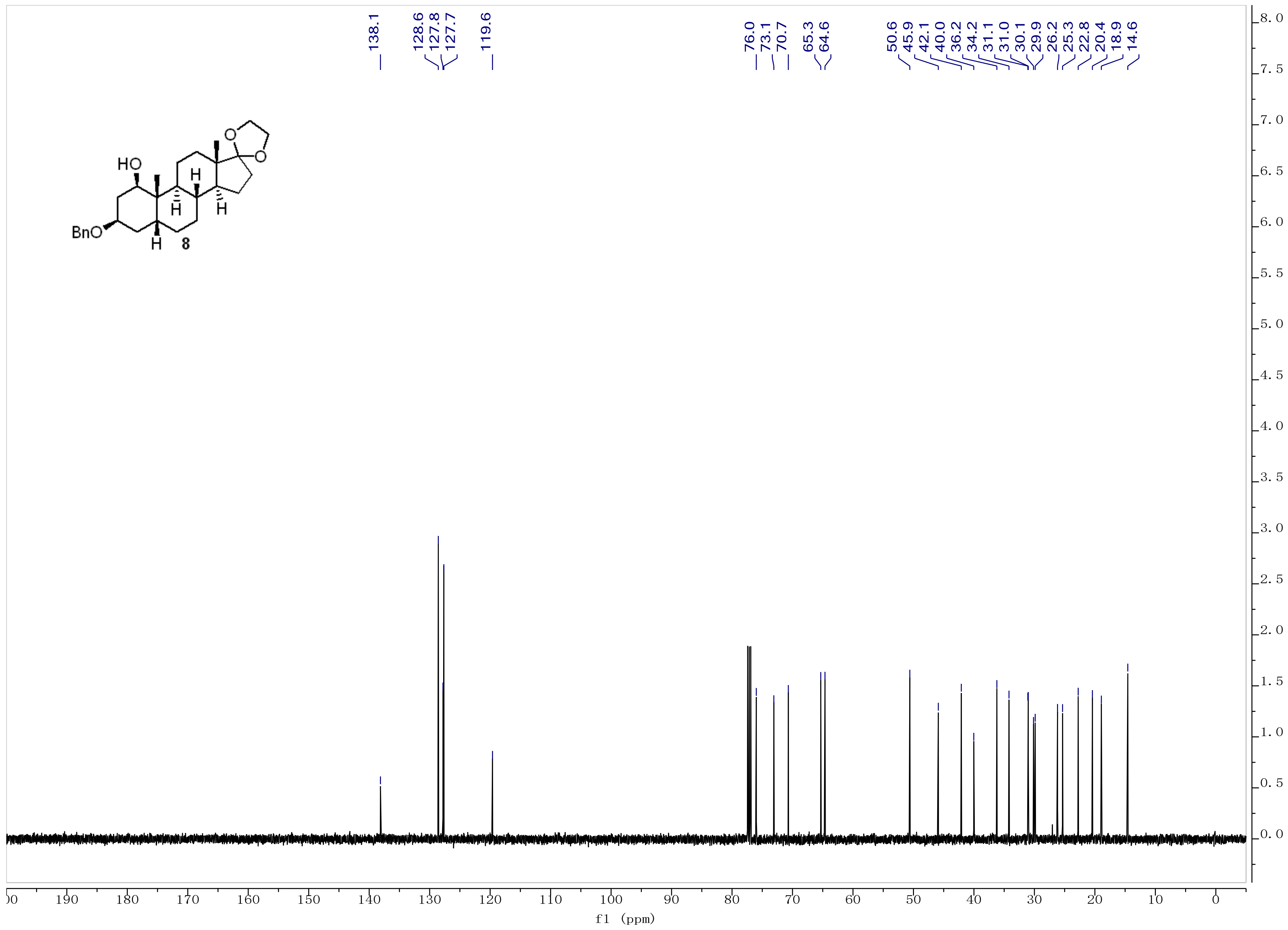

Figure S4 ^{13}C NMR spectrum of compound **8** ($CDCl_3$, 125 MHz)

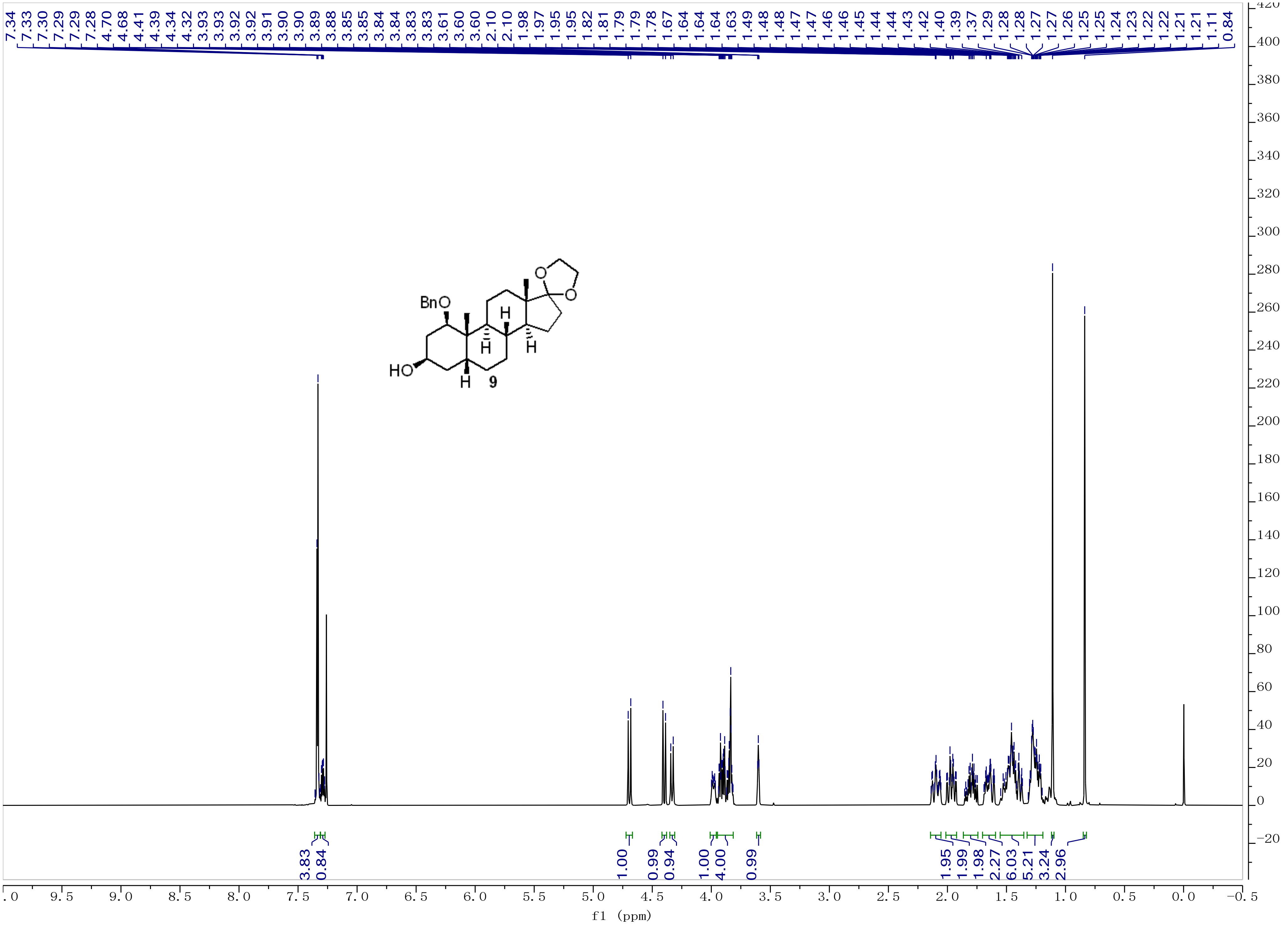

Figure S5 ^{1}H NMR spectrum of compound **9** ($CDCl_3$, 500 MHz)

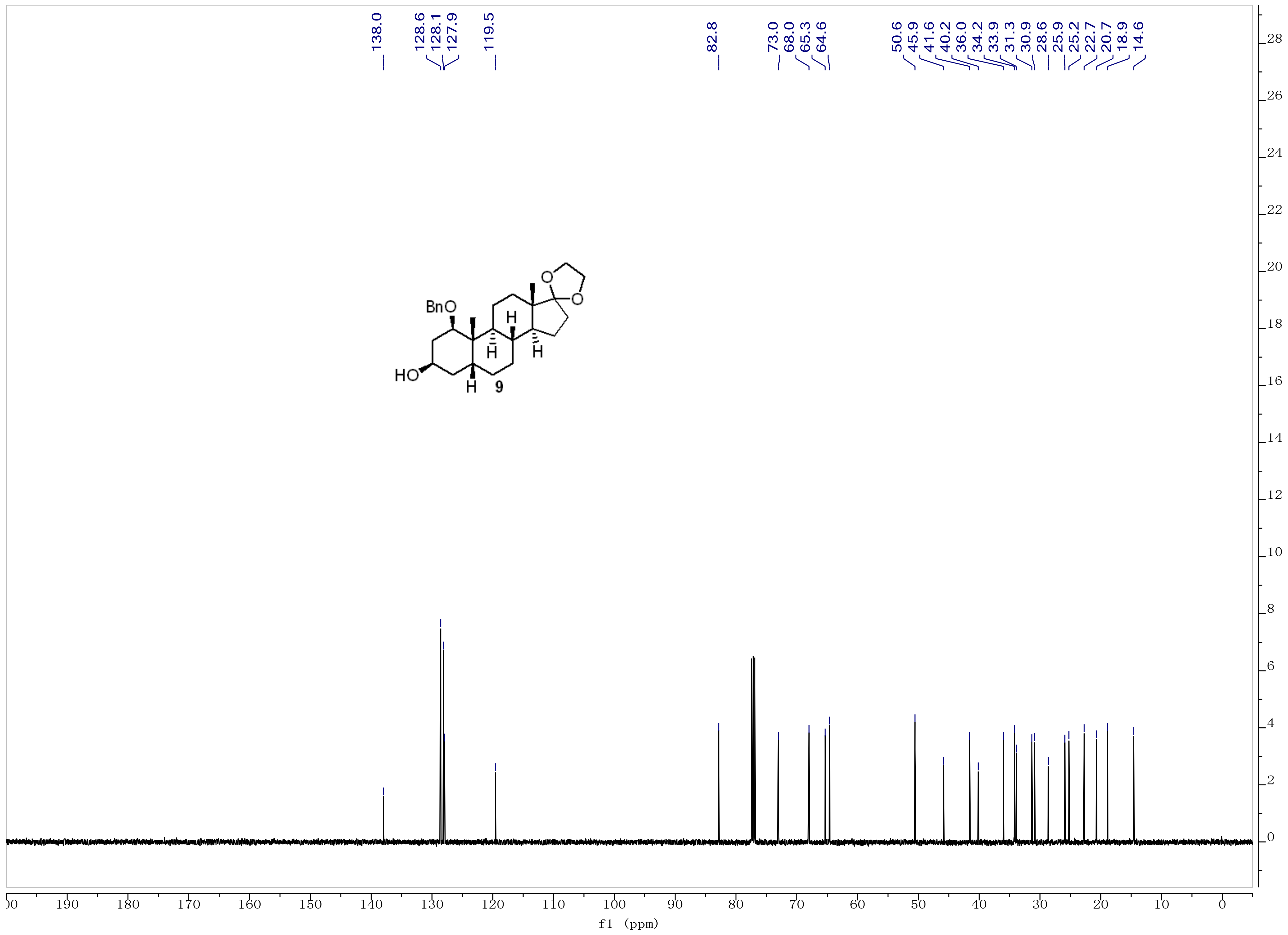

Figure S6 ^{13}C NMR spectrum of compound **9** ($CDCl_3$, 125 MHz)

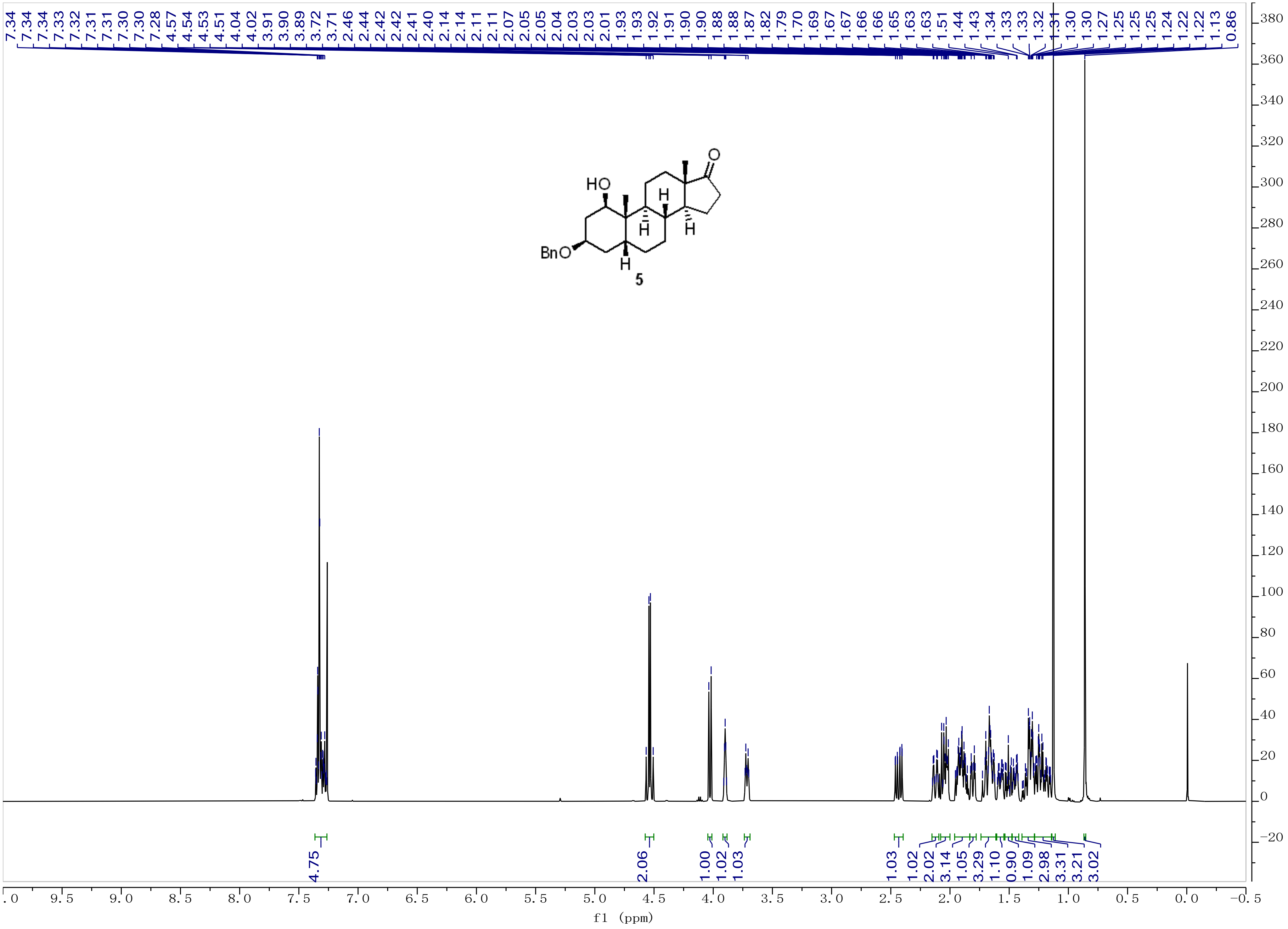

Figure S7 ^{1}H NMR spectrum of compound **5** ($CDCl_3$, 500 MHz)

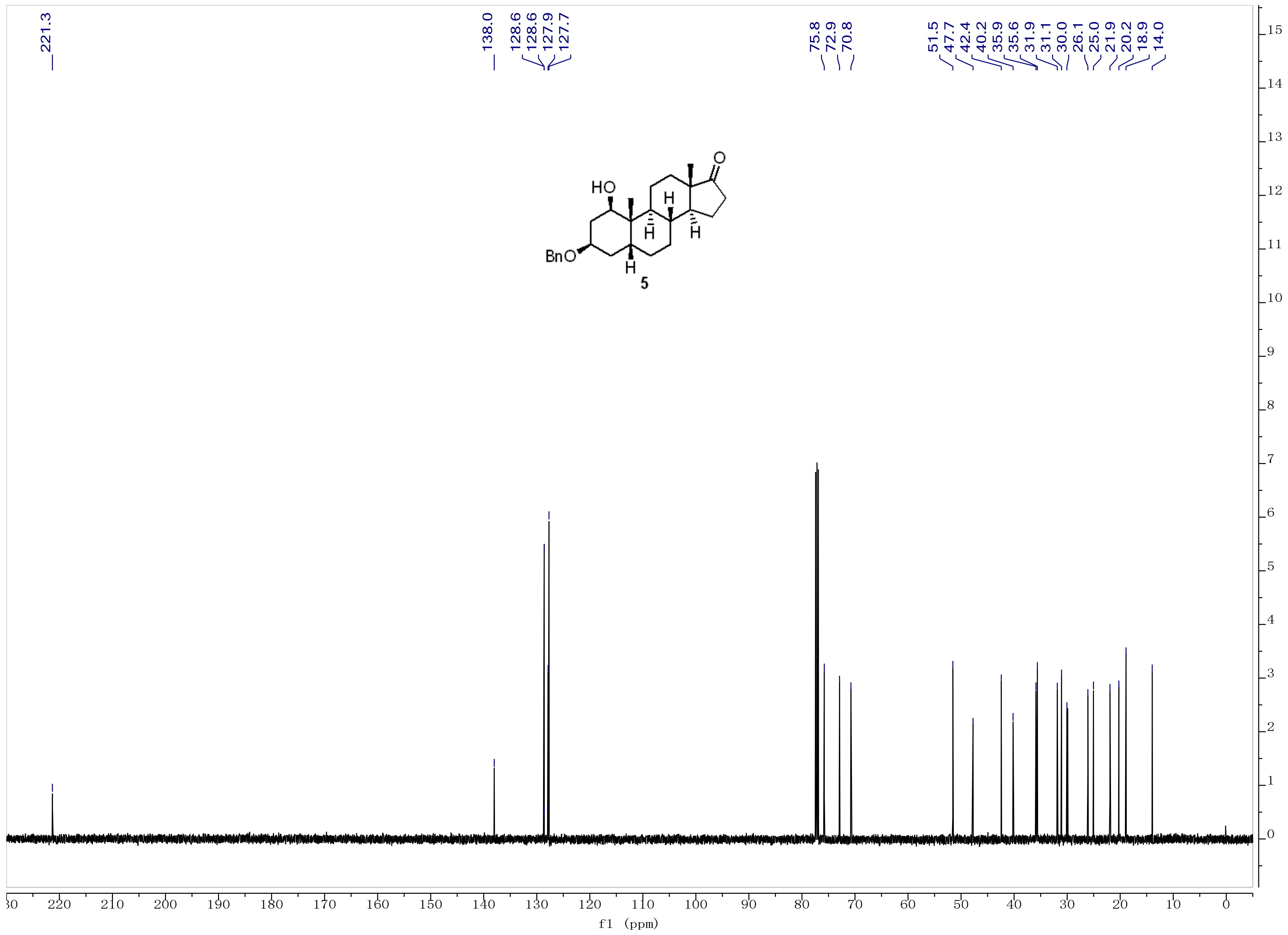

Figure S8 ^{13}C NMR spectrum of compound **5** ($CDCl_3$, 125 MHz)

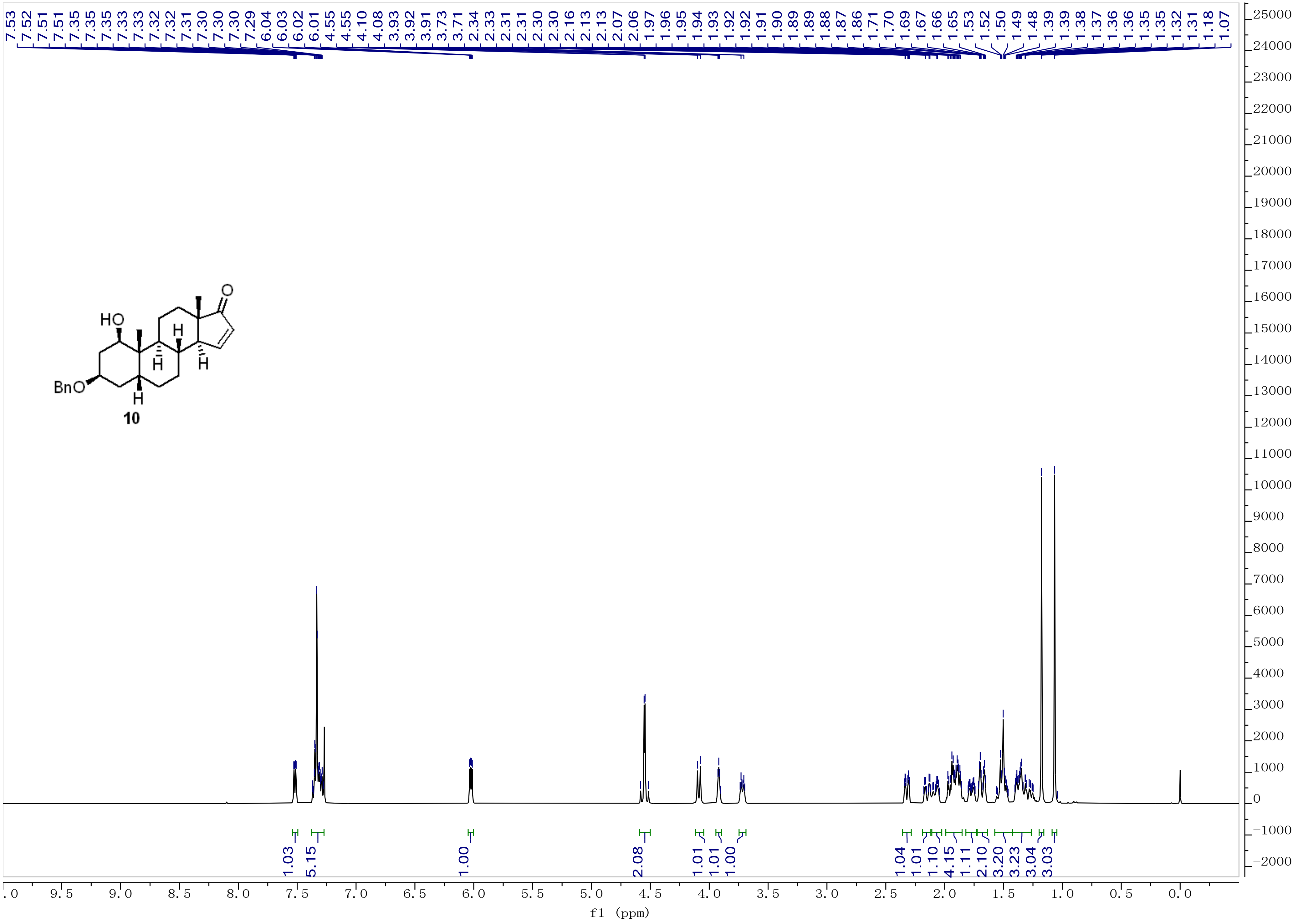

Figure S9 ^{1}H NMR spectrum of compound **10** ($CDCl_3$, 400 MHz)

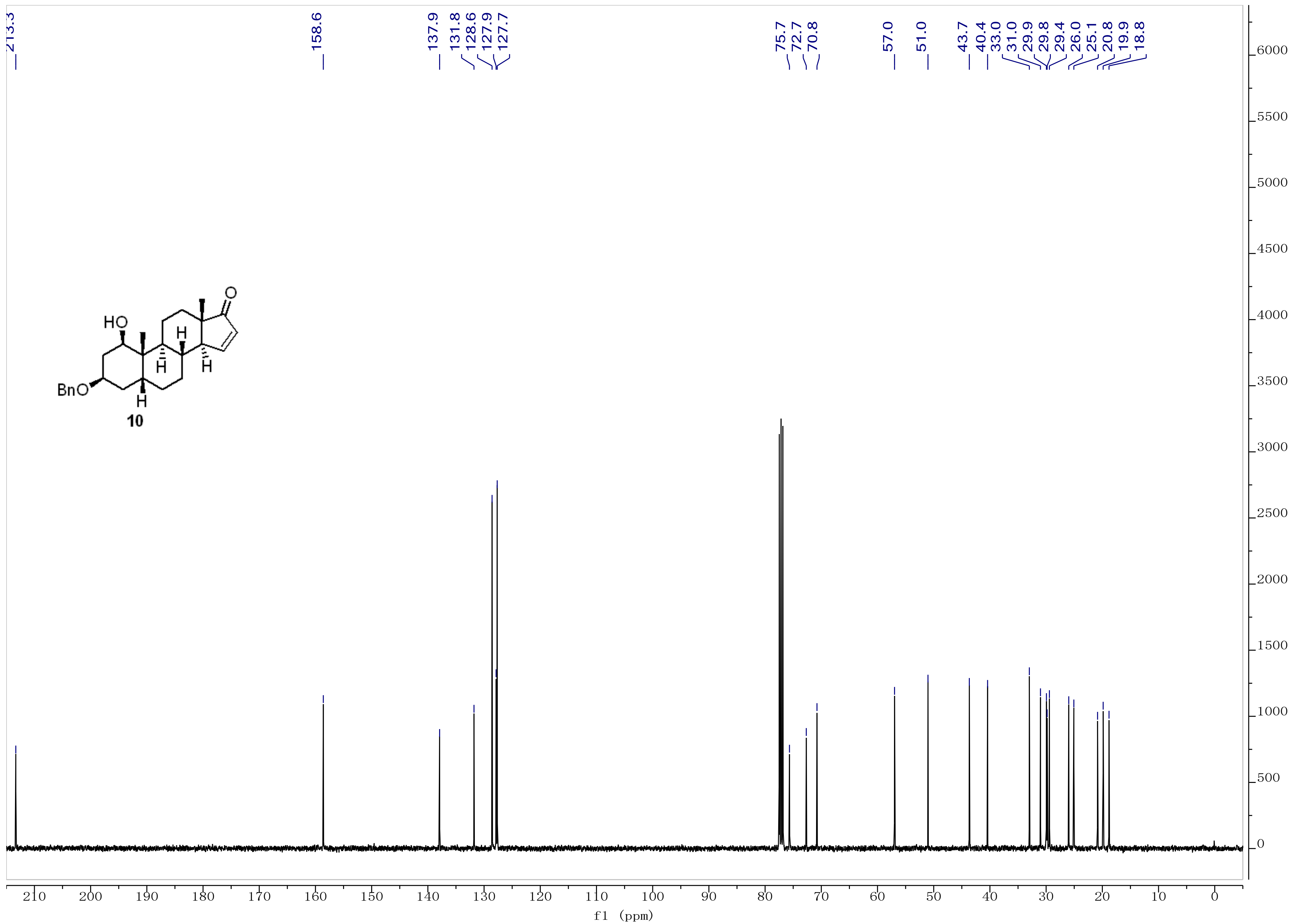

10

Figure S10 ^{13}C NMR spectrum of compound **10** ($CDCl_3$, 100 MHz)

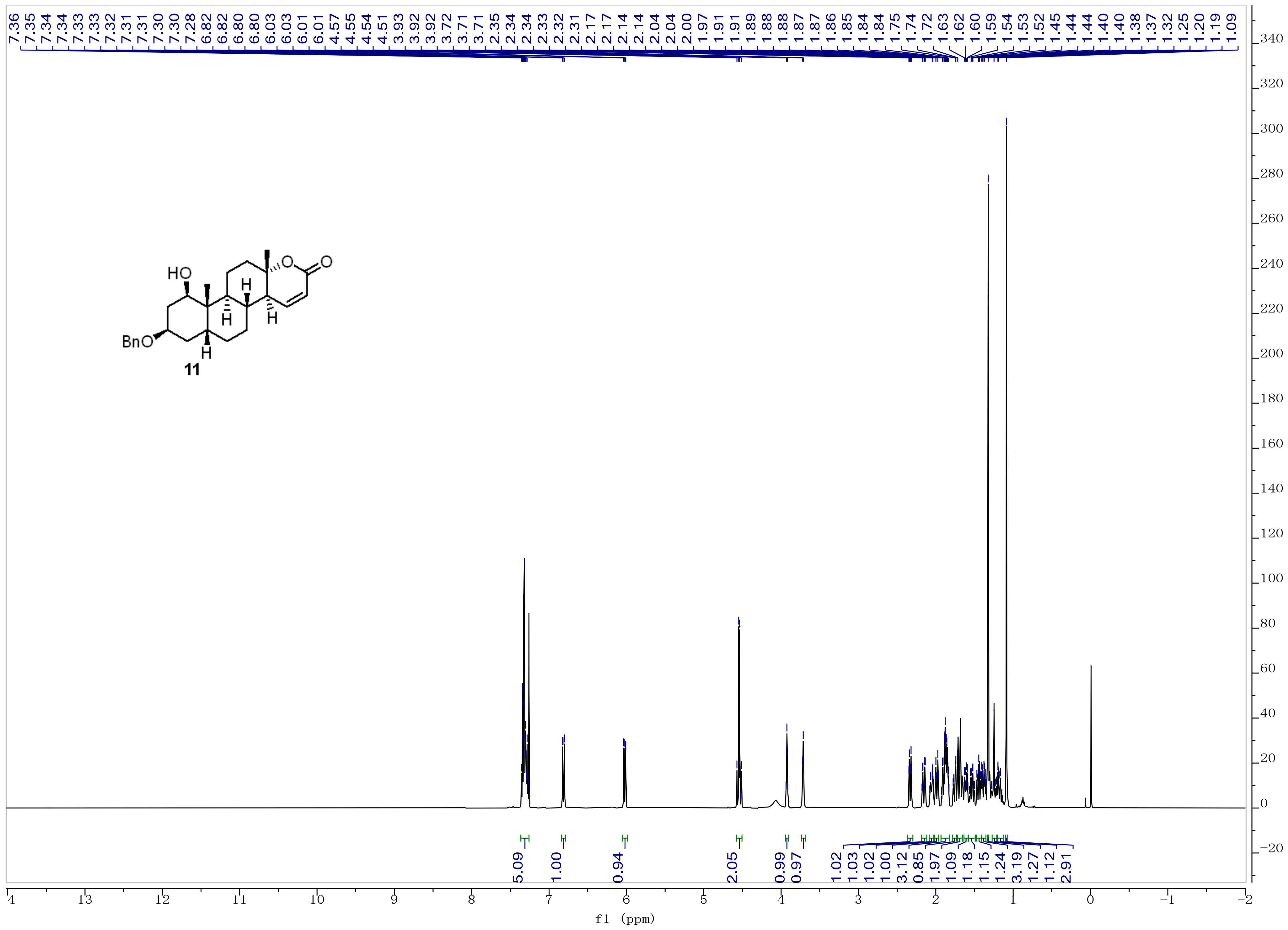

Figure S11 ^{1}H NMR spectrum of compound **11** ($CDCl_3$, 500 MHz)

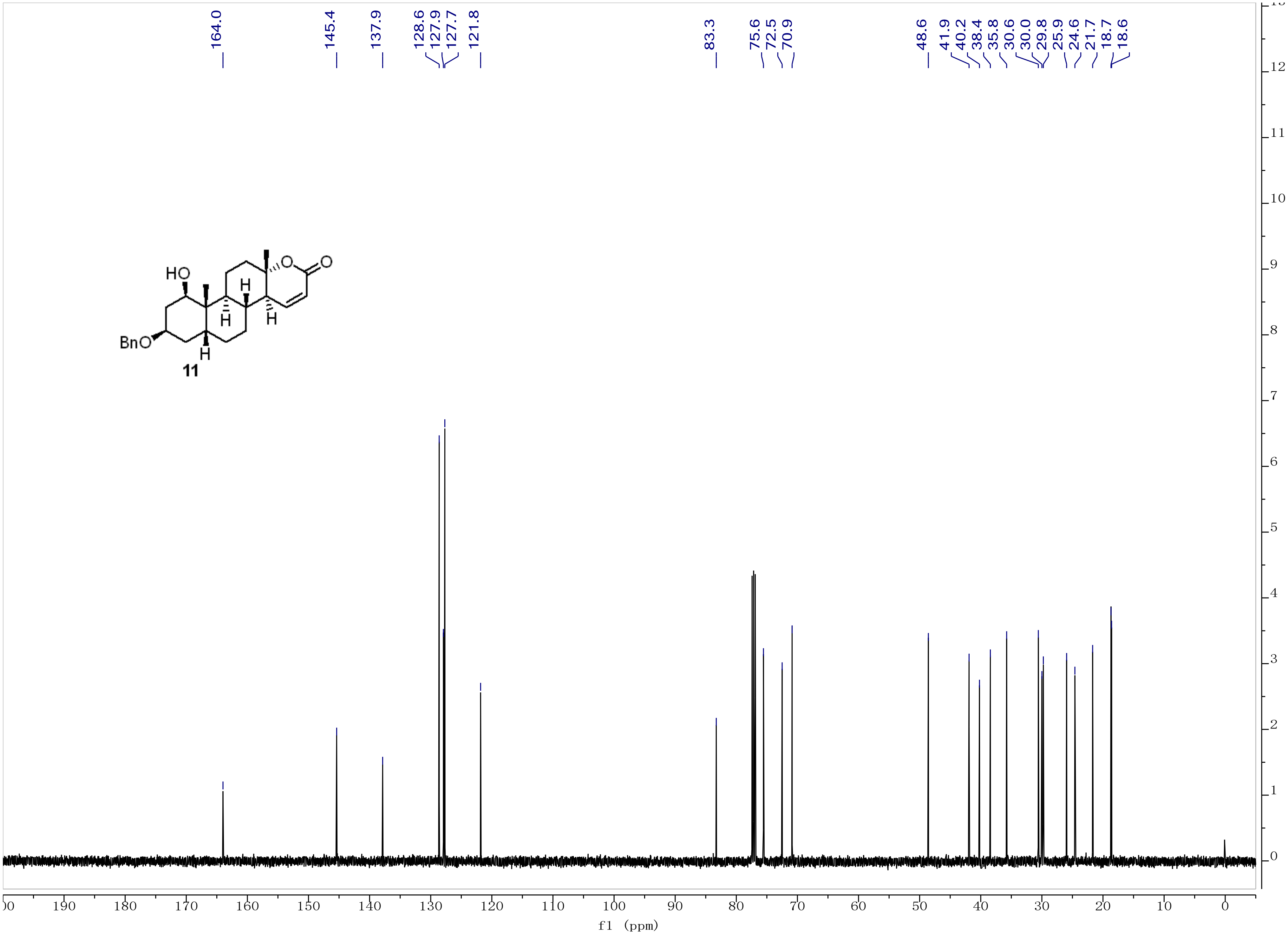

Figure S12 ^{13}C NMR spectrum of compound **11** ($CDCl_3$, 125 MHz)

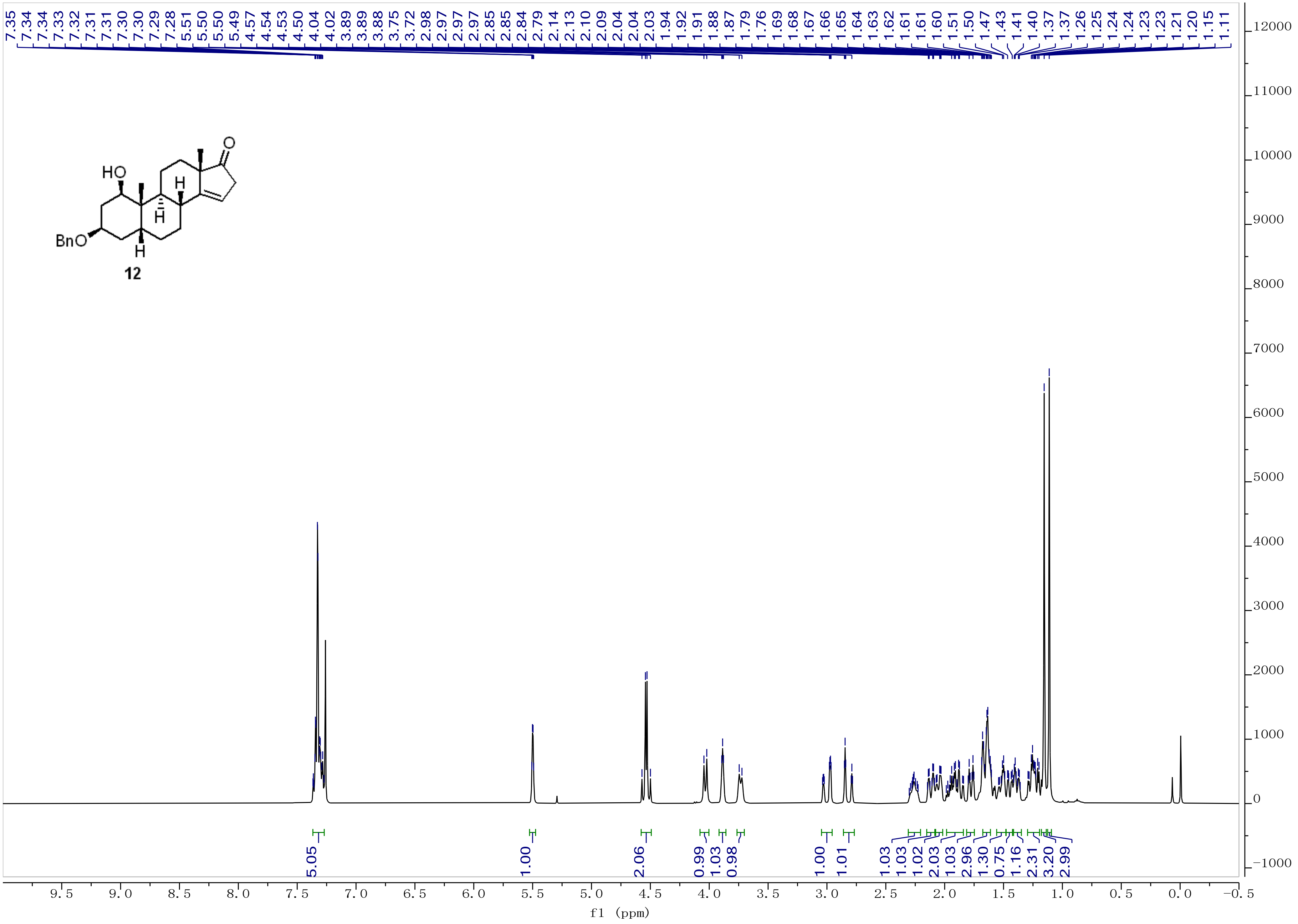

Figure S13 ^{1}H NMR spectrum of compound **12** ($CDCl_3$, 400 MHz)

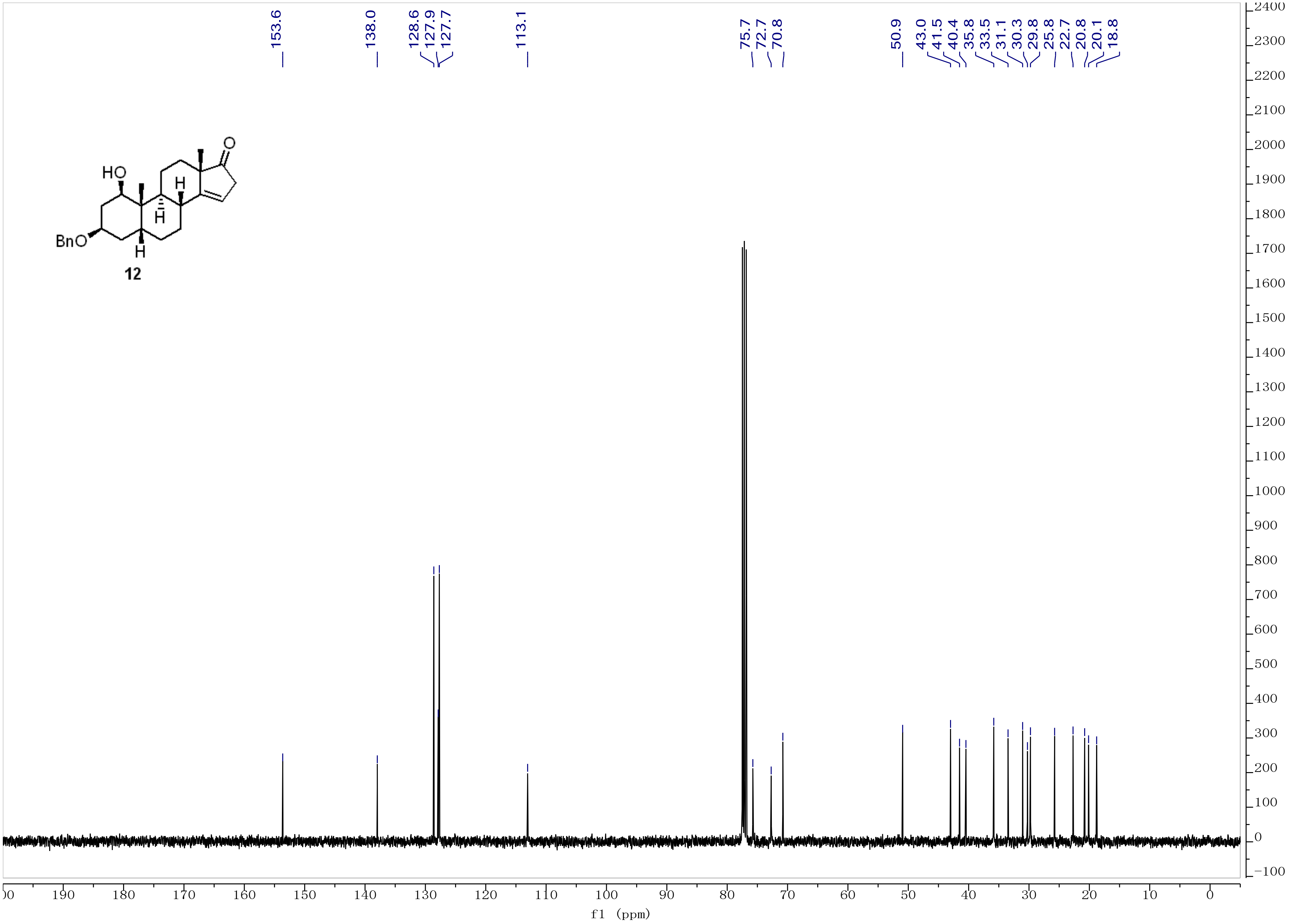

Figure S14 ^{13}C NMR spectrum of compound **12** ($CDCl_3$, 100 MHz)

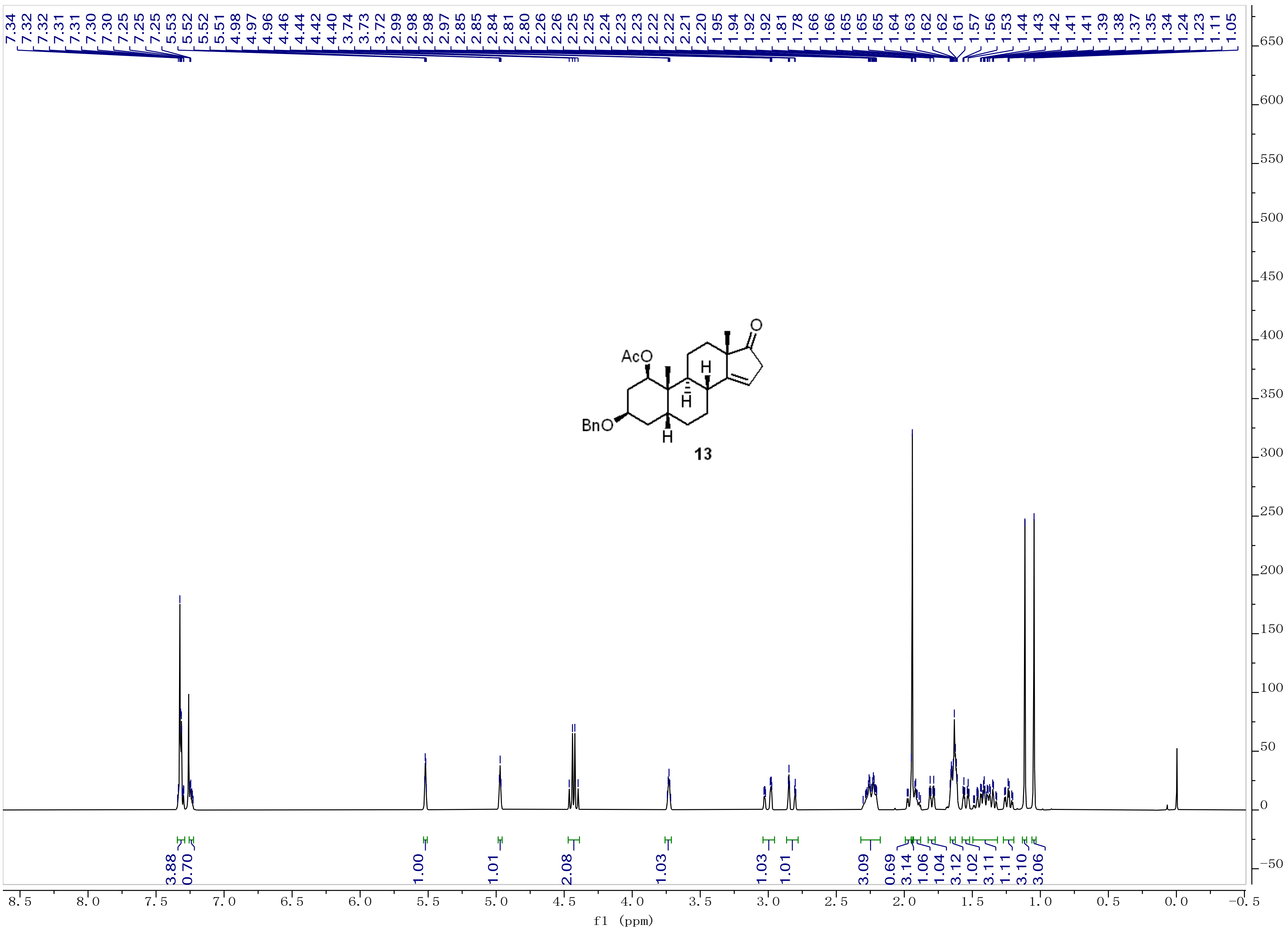

Figure S15 ^{1}H NMR spectrum of compound **13** ($CDCl_3$, 500 MHz)

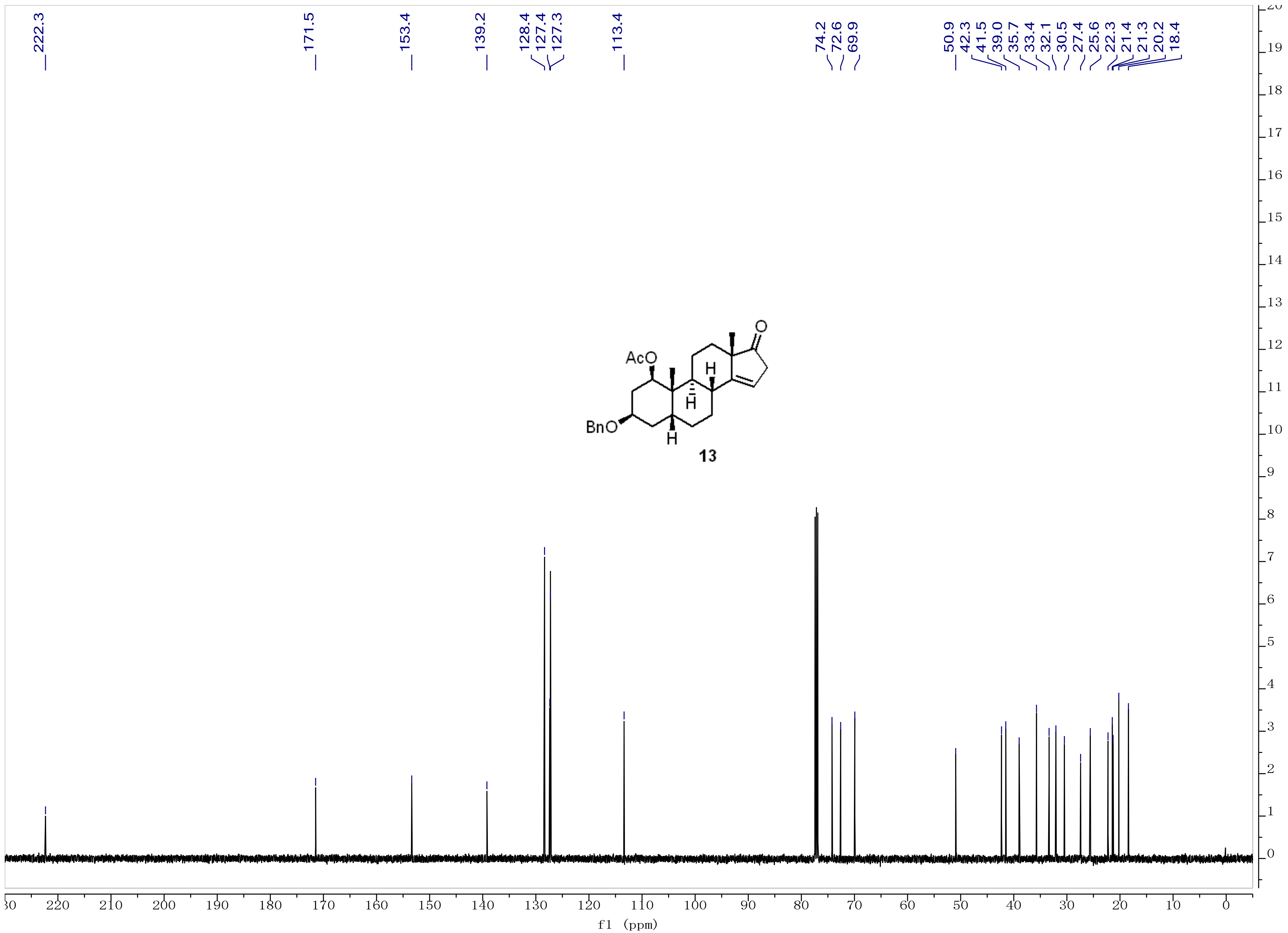

Figure S16 ^{13}C NMR spectrum of compound **13** ($CDCl_3$, 125 MHz)

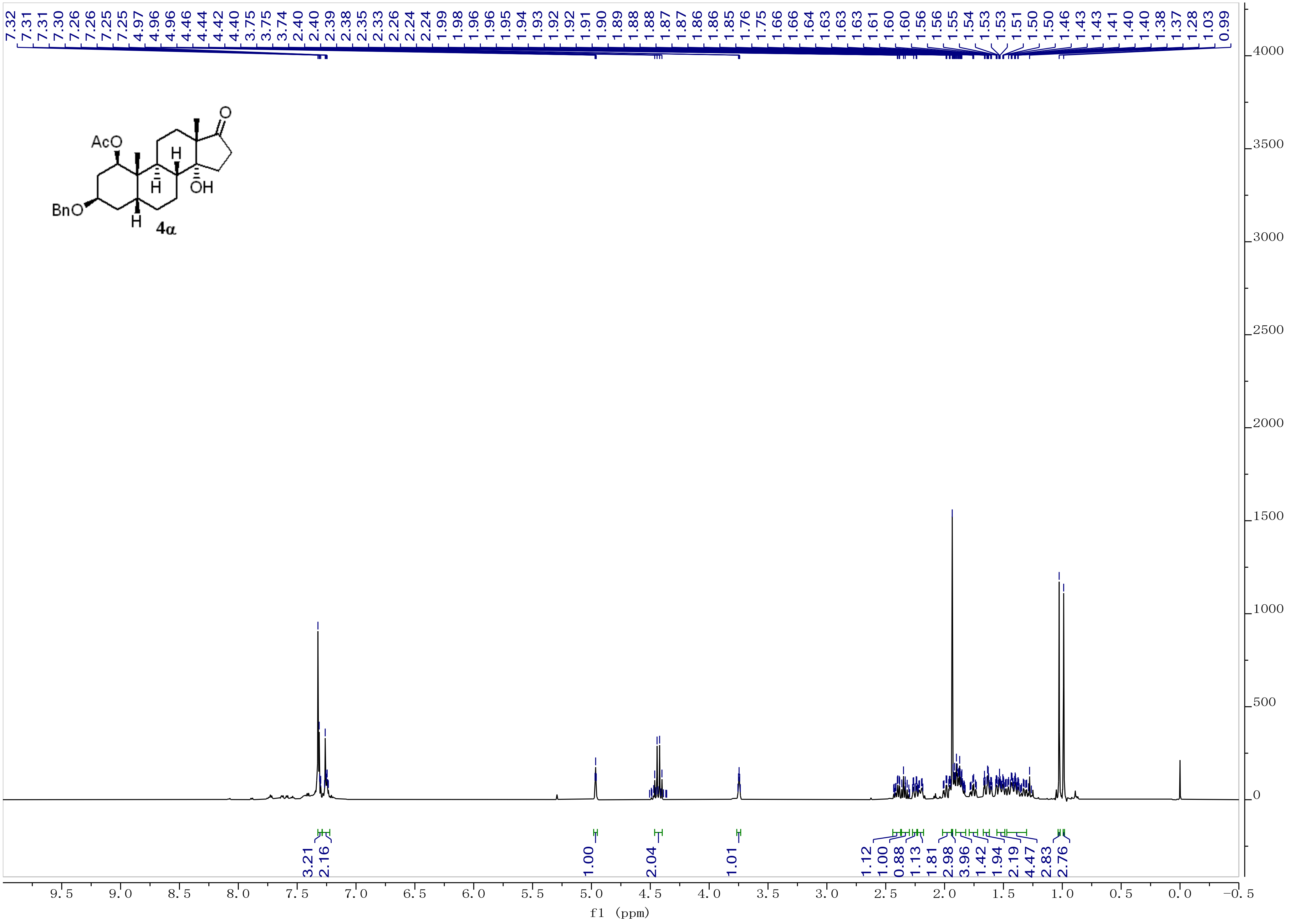

Figure S17 ^{1}H NMR spectrum of compound **4α** ($CDCl_3$, 600 MHz)

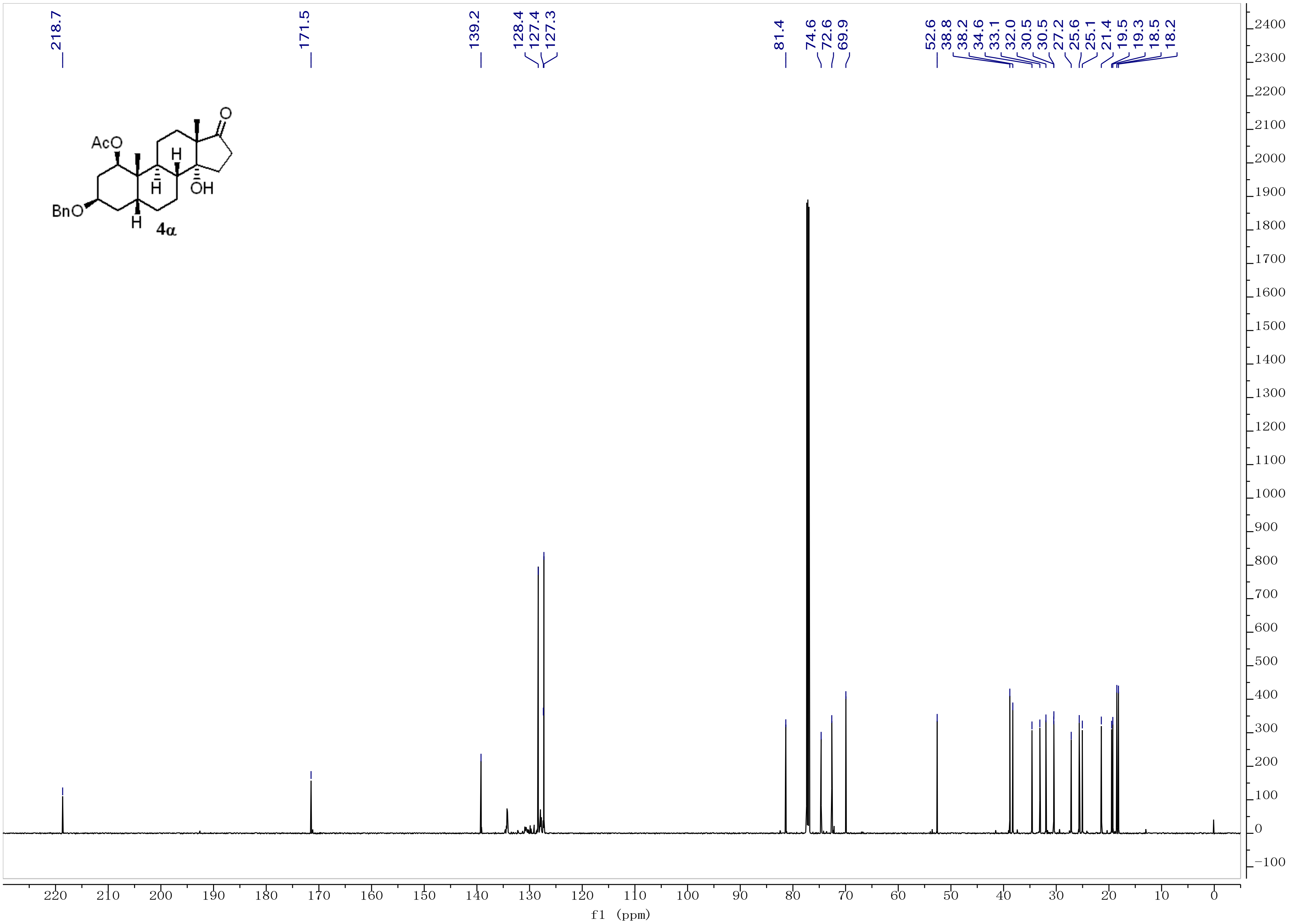

Figure S18 ^{13}C NMR spectrum of compound **4α** ($CDCl_3$, 150 MHz)

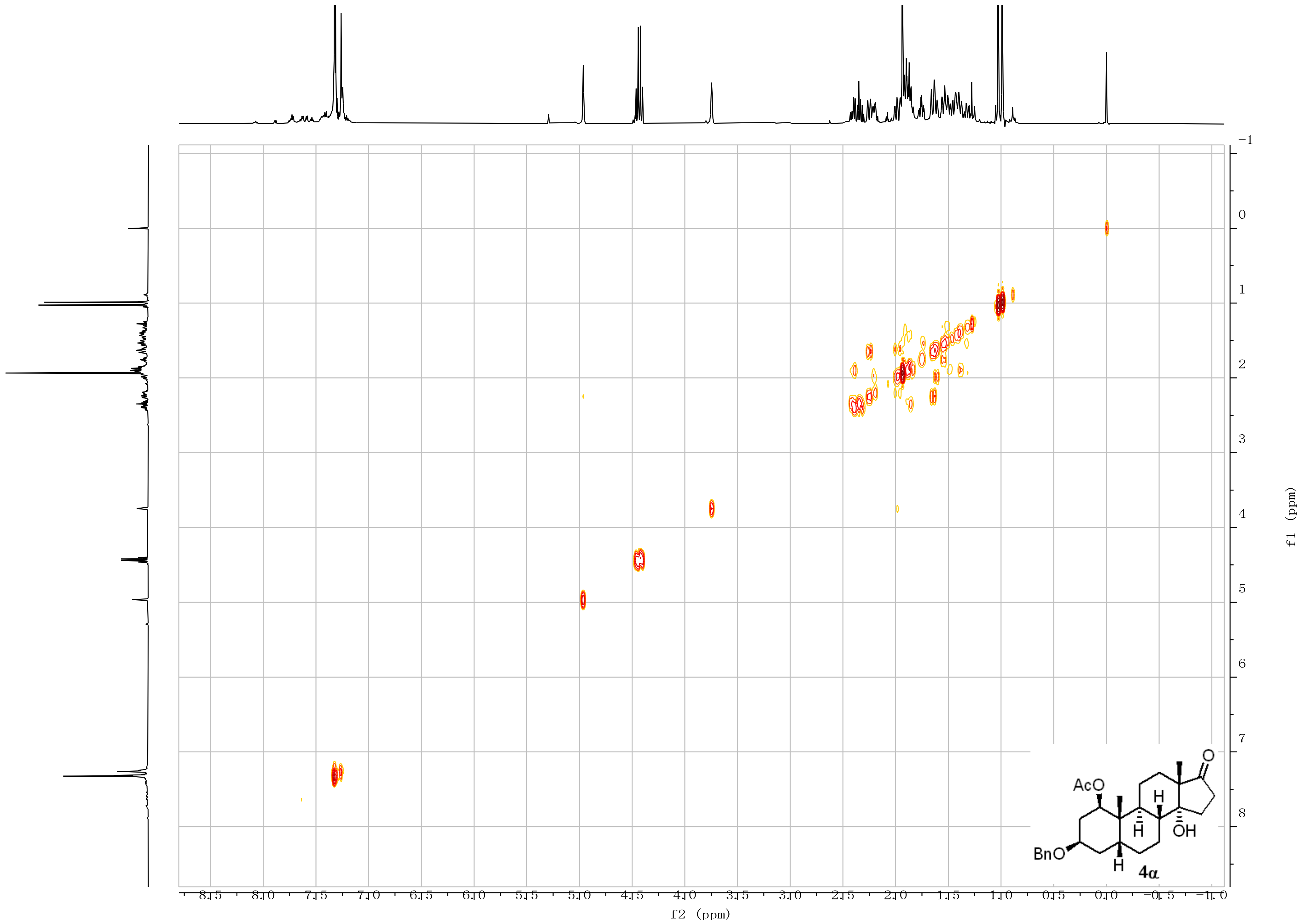

Figure S19 COSY spectrum of compound **4α** ($CDCl_3$, 600 MHz)

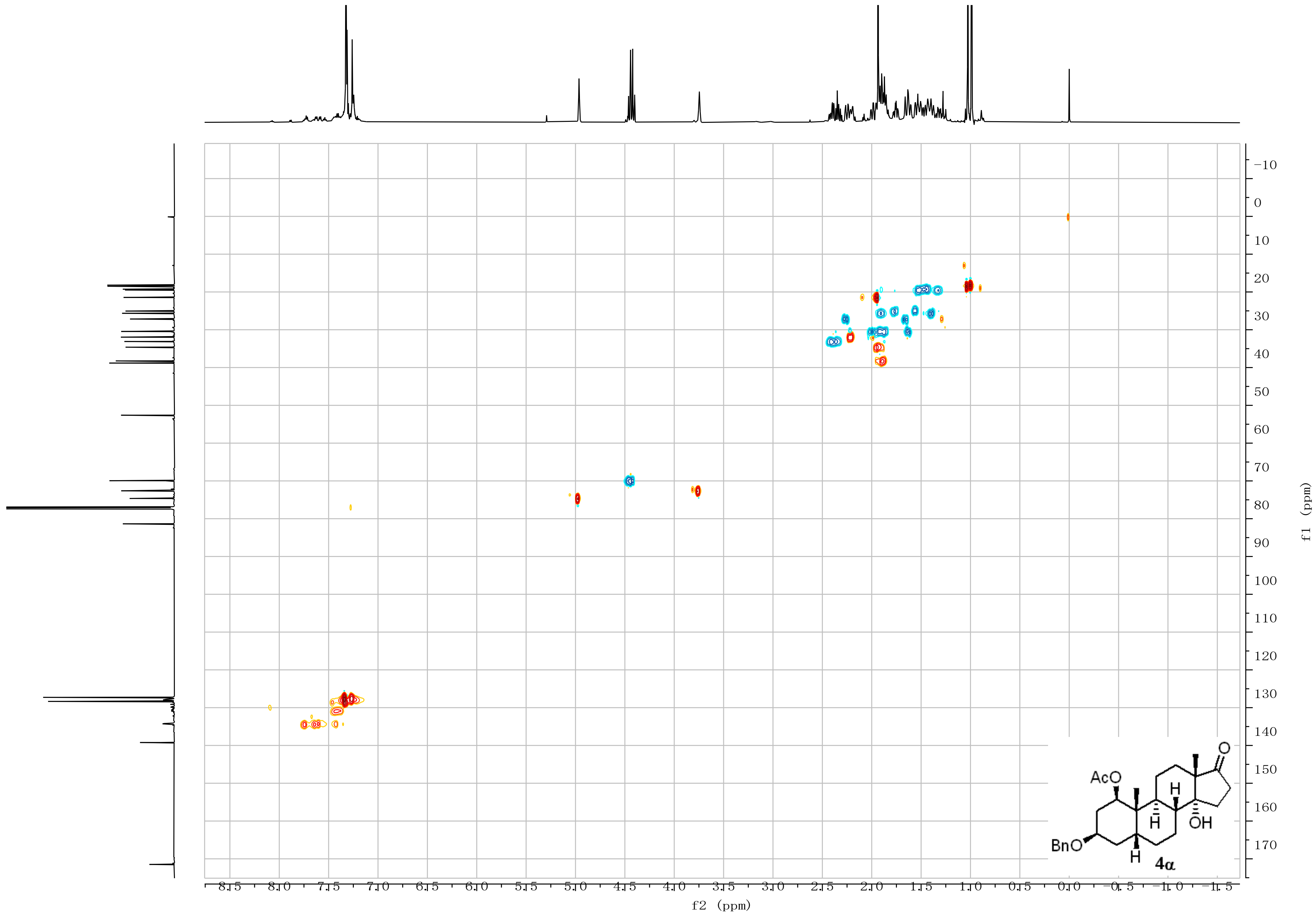

Figure S20 HSQC spectrum of compound **4α** ($CDCl_3$, 600 MHz)

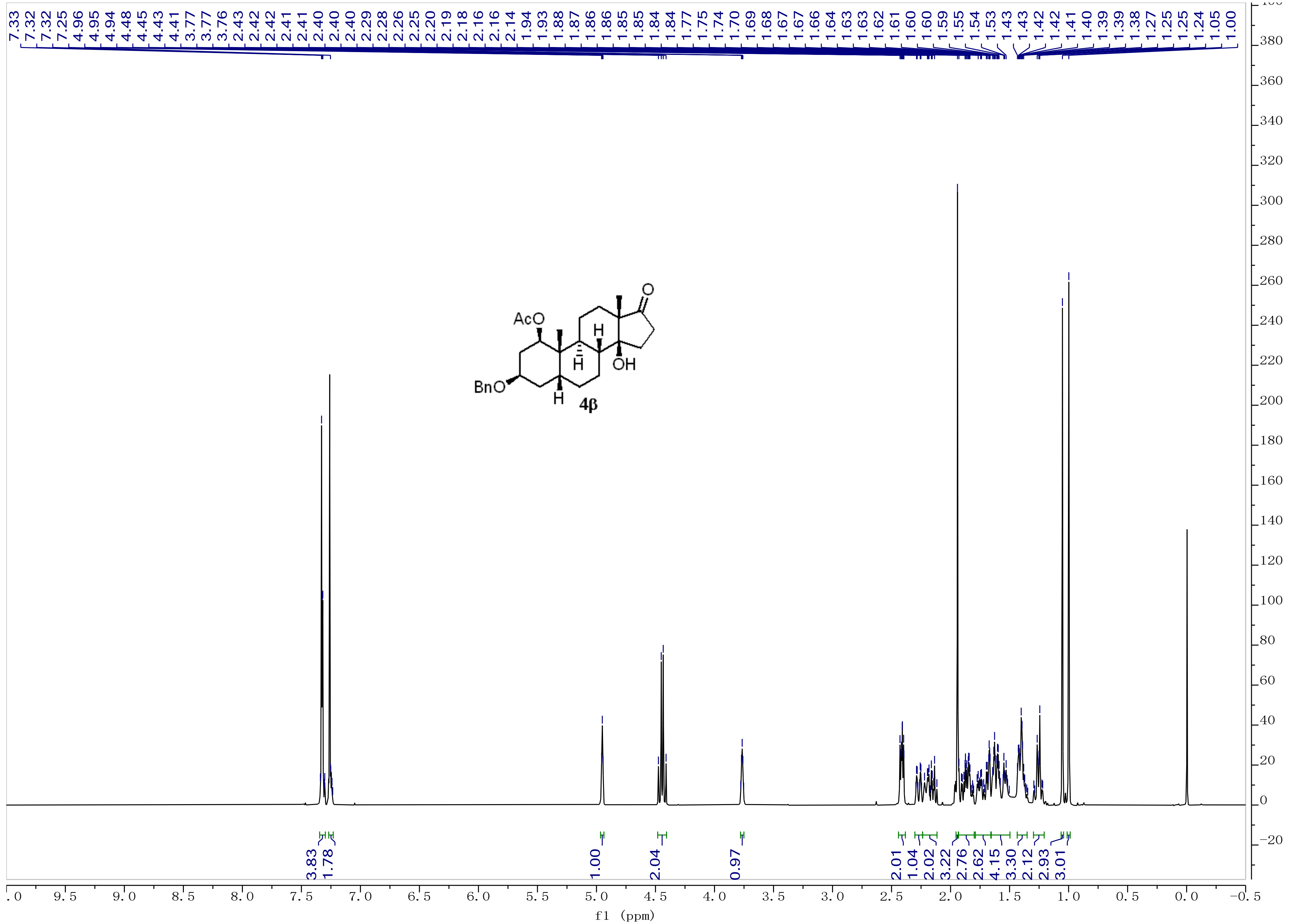

Figure S21 ^{1}H NMR spectrum of compound **4β** ($CDCl_3$, 500 MHz)

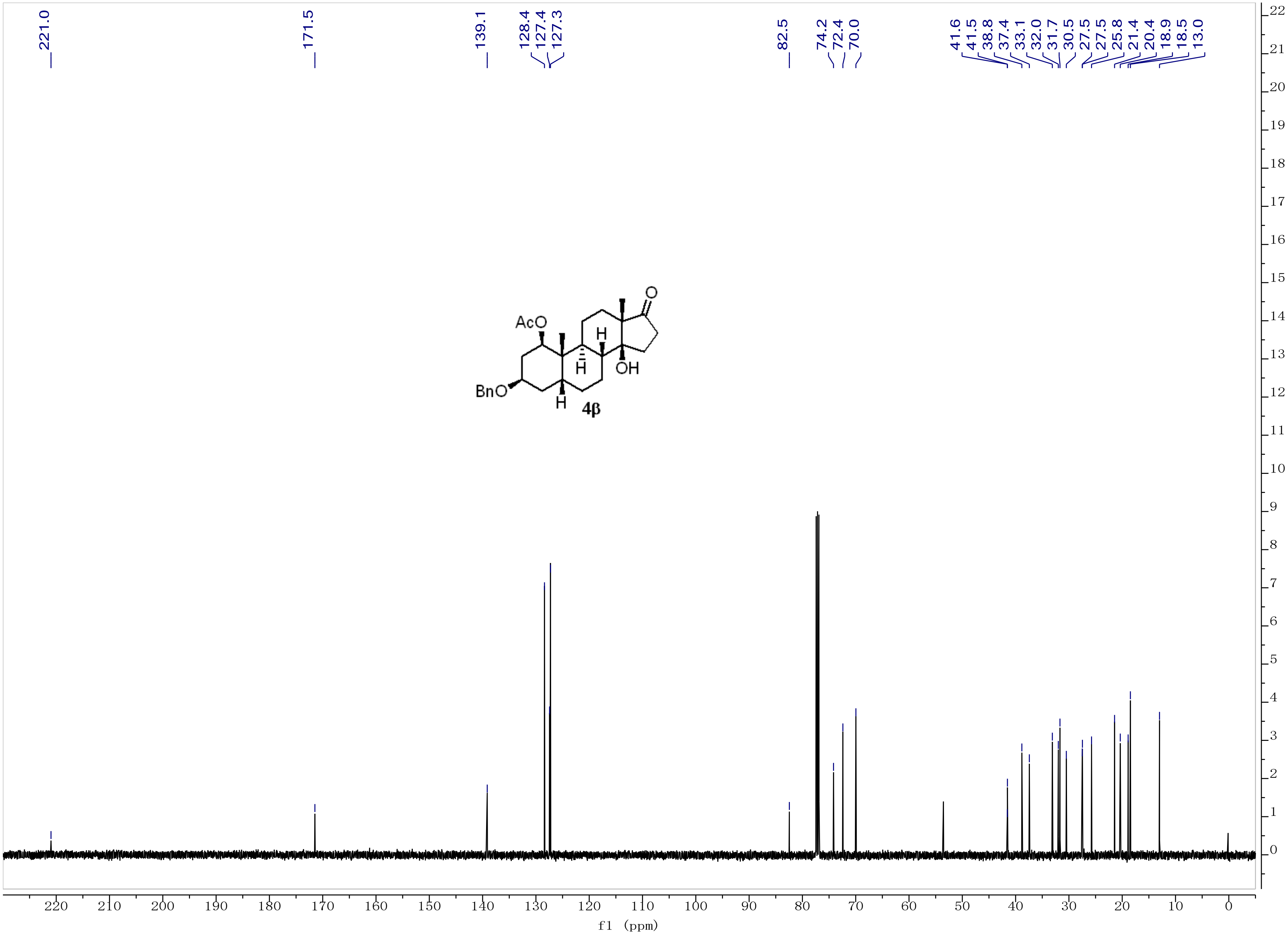

Figure S22 ^{13}C NMR spectrum of compound **4β** ($CDCl_3$, 125 MHz)

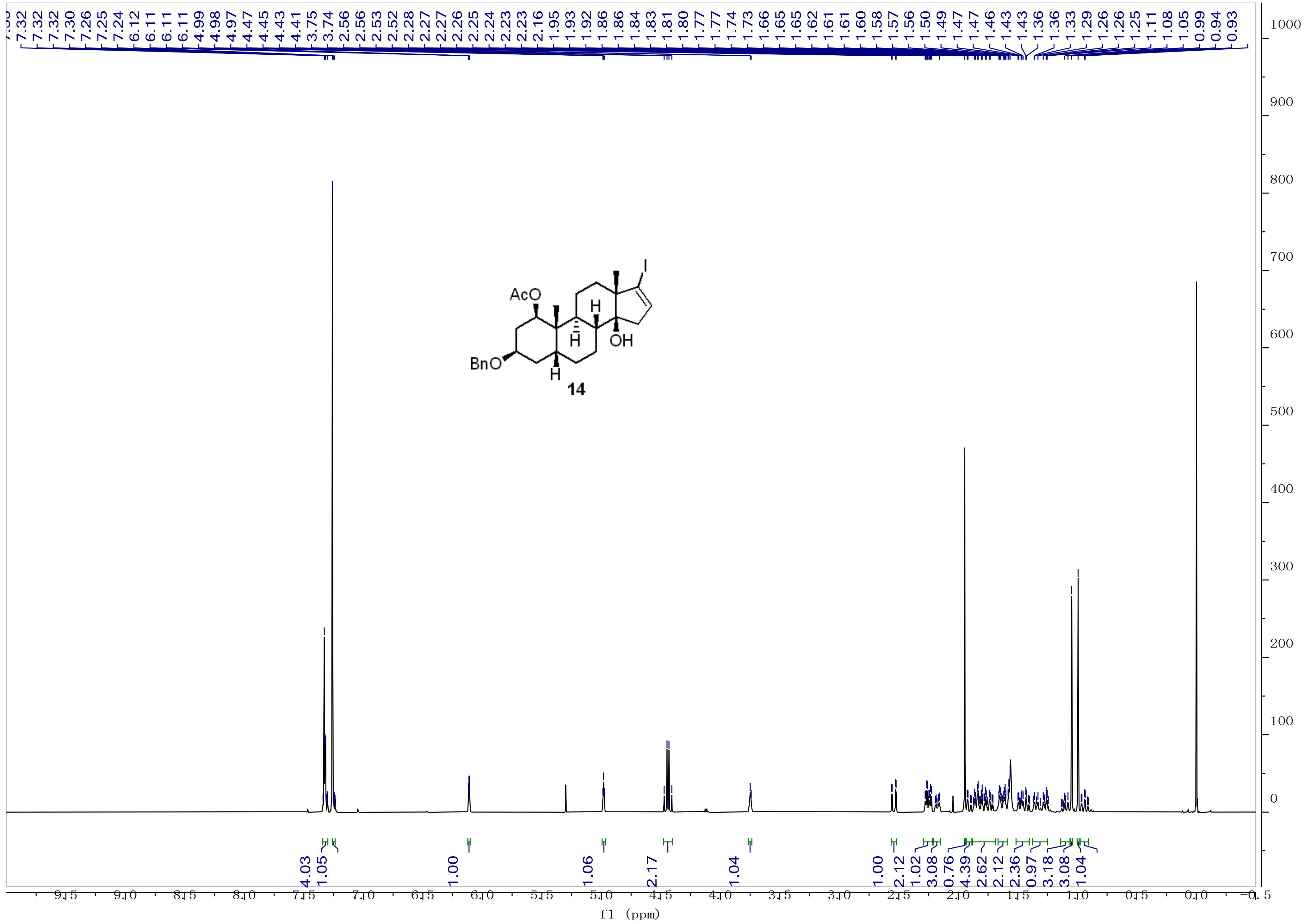

Figure S23 ^{1}H NMR spectrum of compound **14** ($CDCl_3$, 500 MHz)

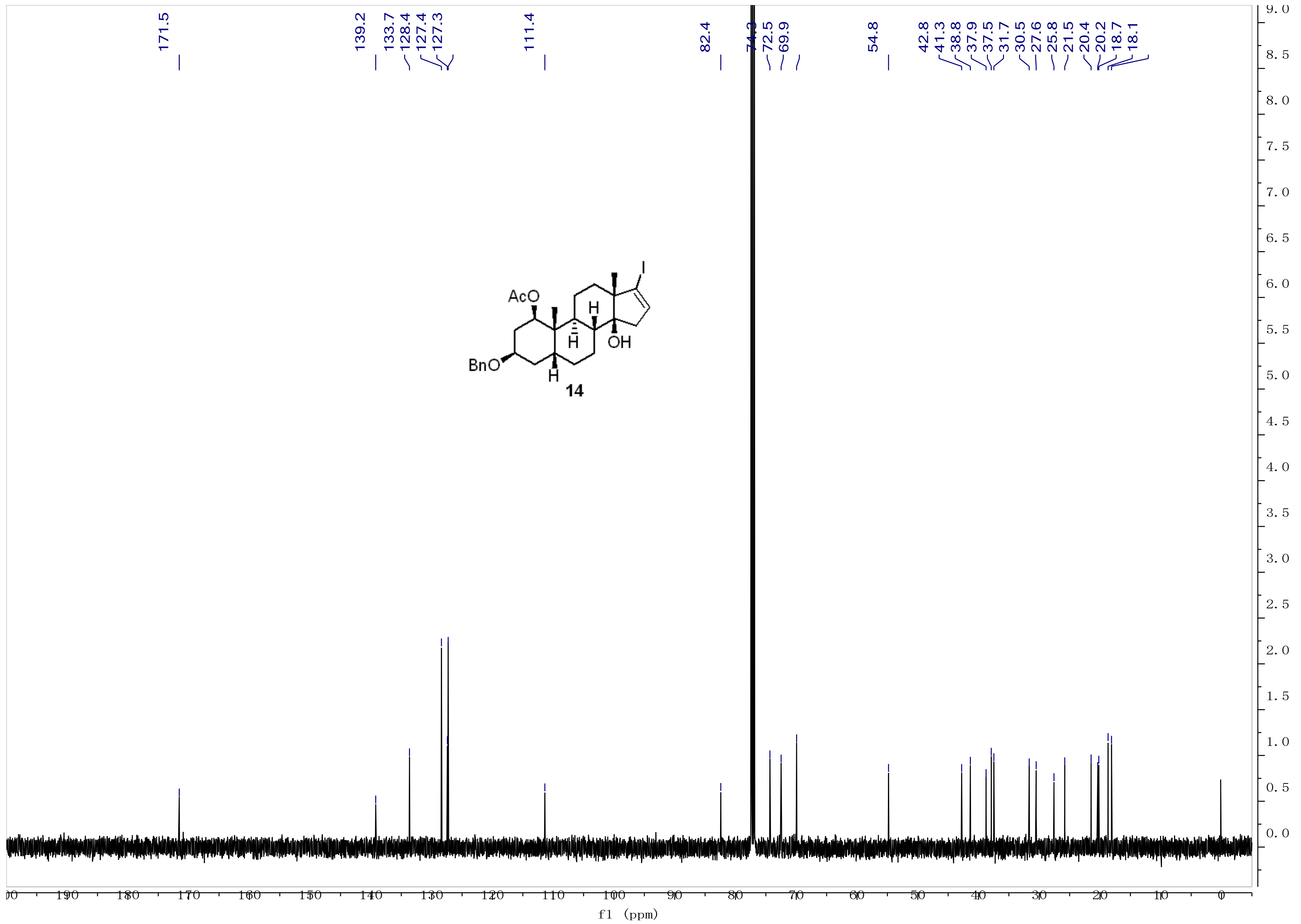

Figure S24 ^{13}C NMR spectrum of compound **14** ($CDCl_3$, 125 MHz)

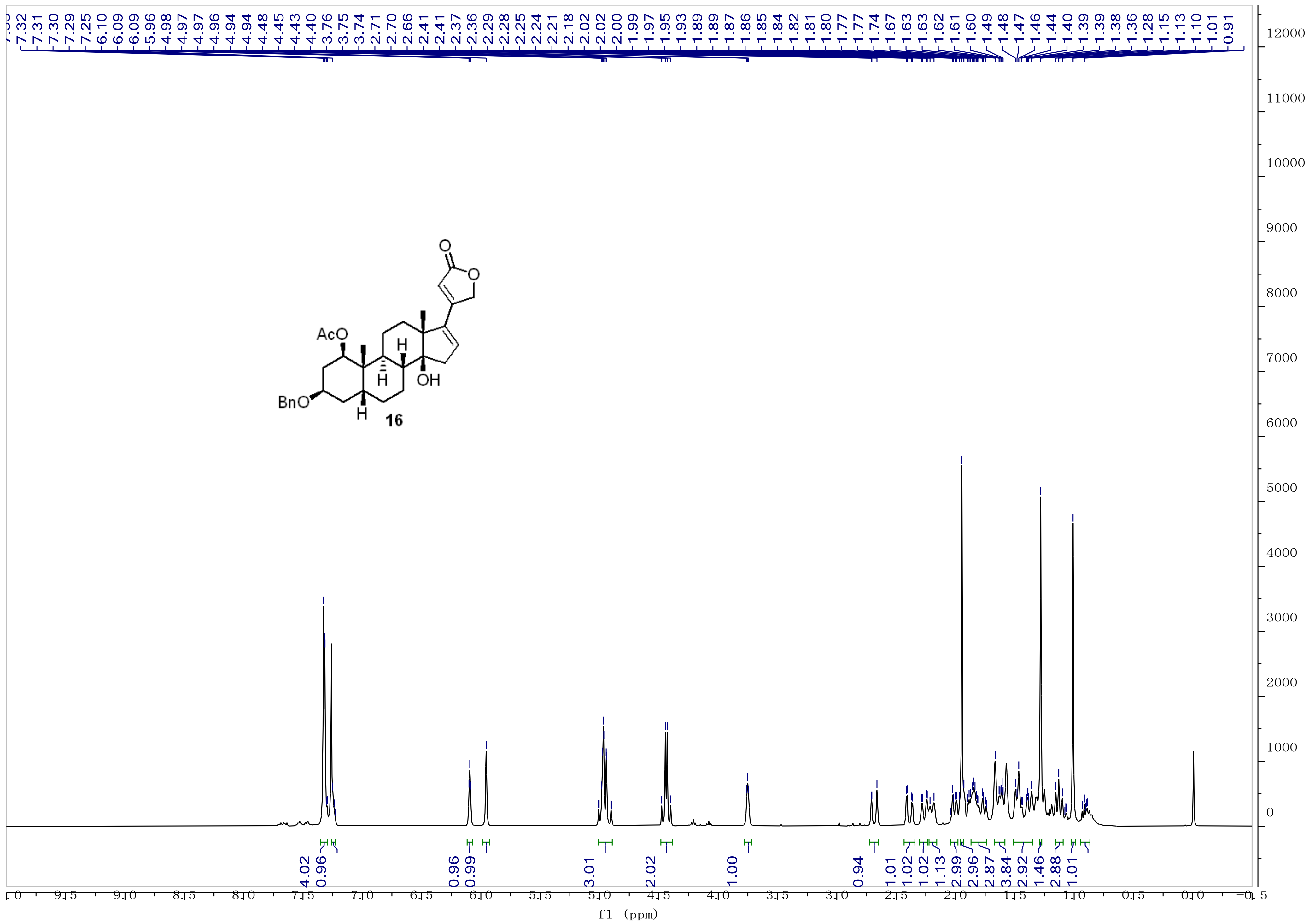

Figure S25 ^{1}H NMR spectrum of compound **16** ($CDCl_3$, 400 MHz)

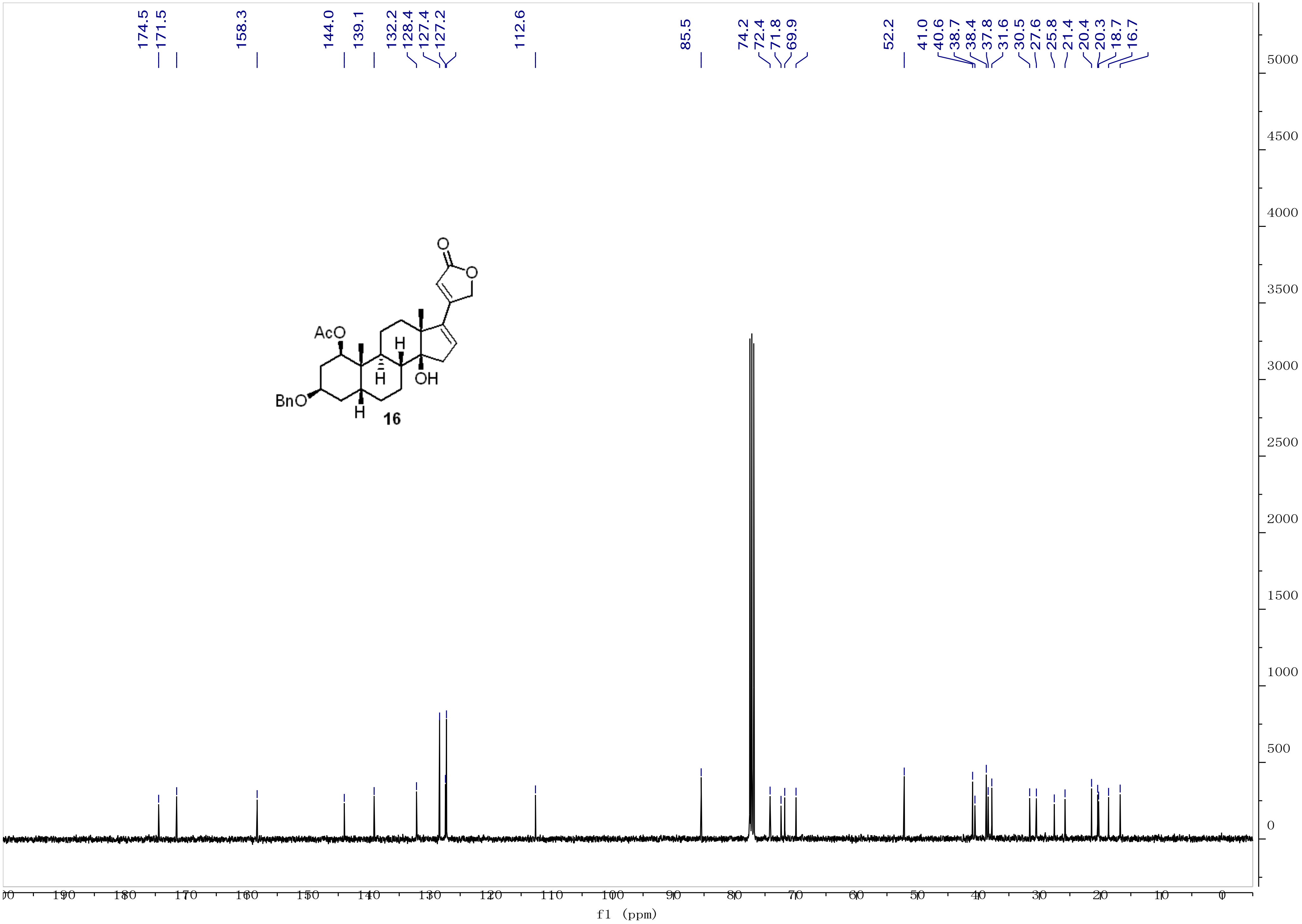

Figure S26 ^{13}C NMR spectrum of compound **16** ($CDCl_3$, 100 MHz)

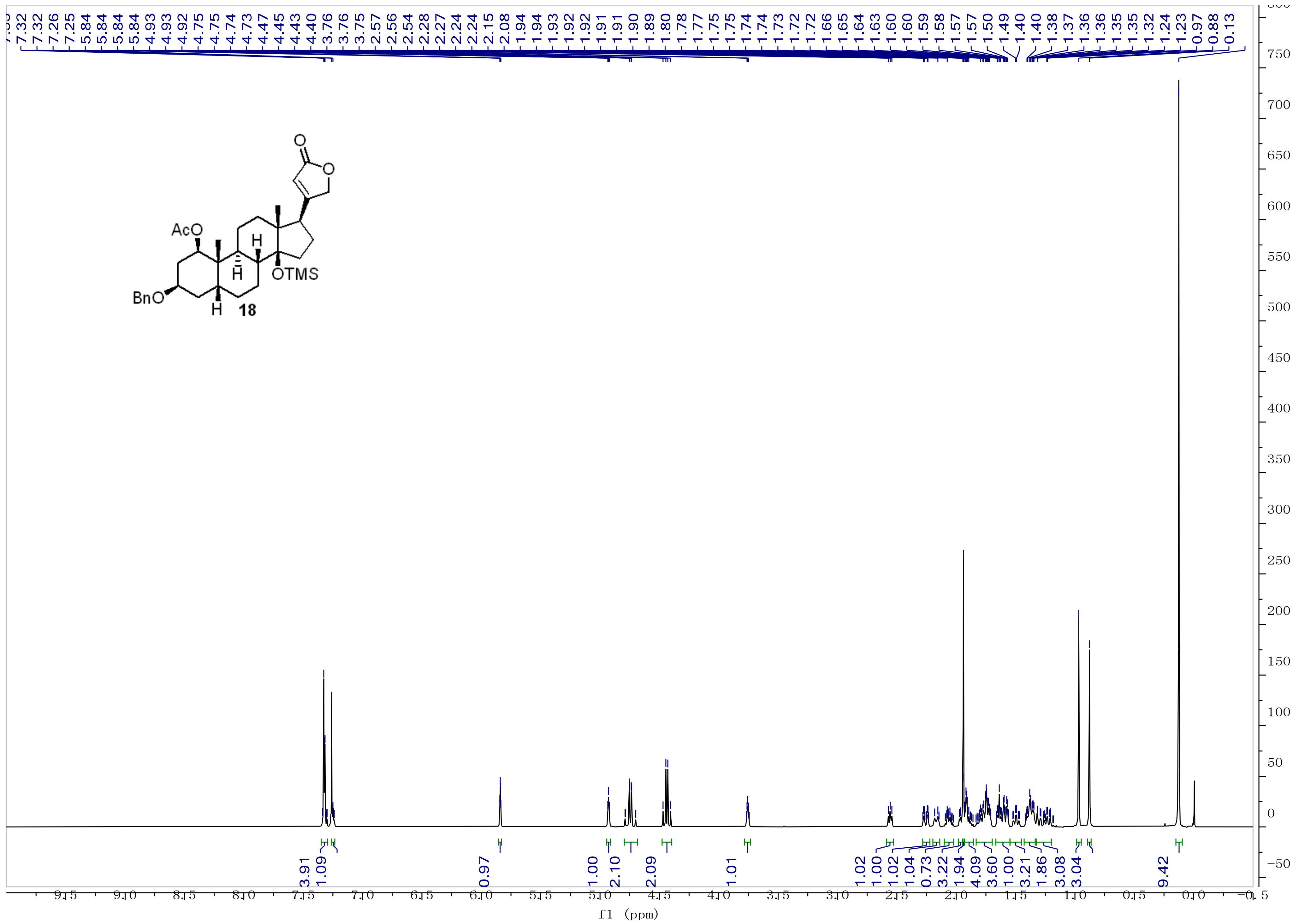

Figure S27 ^{1}H NMR spectrum of compound **18** ($CDCl_3$, 500 MHz)

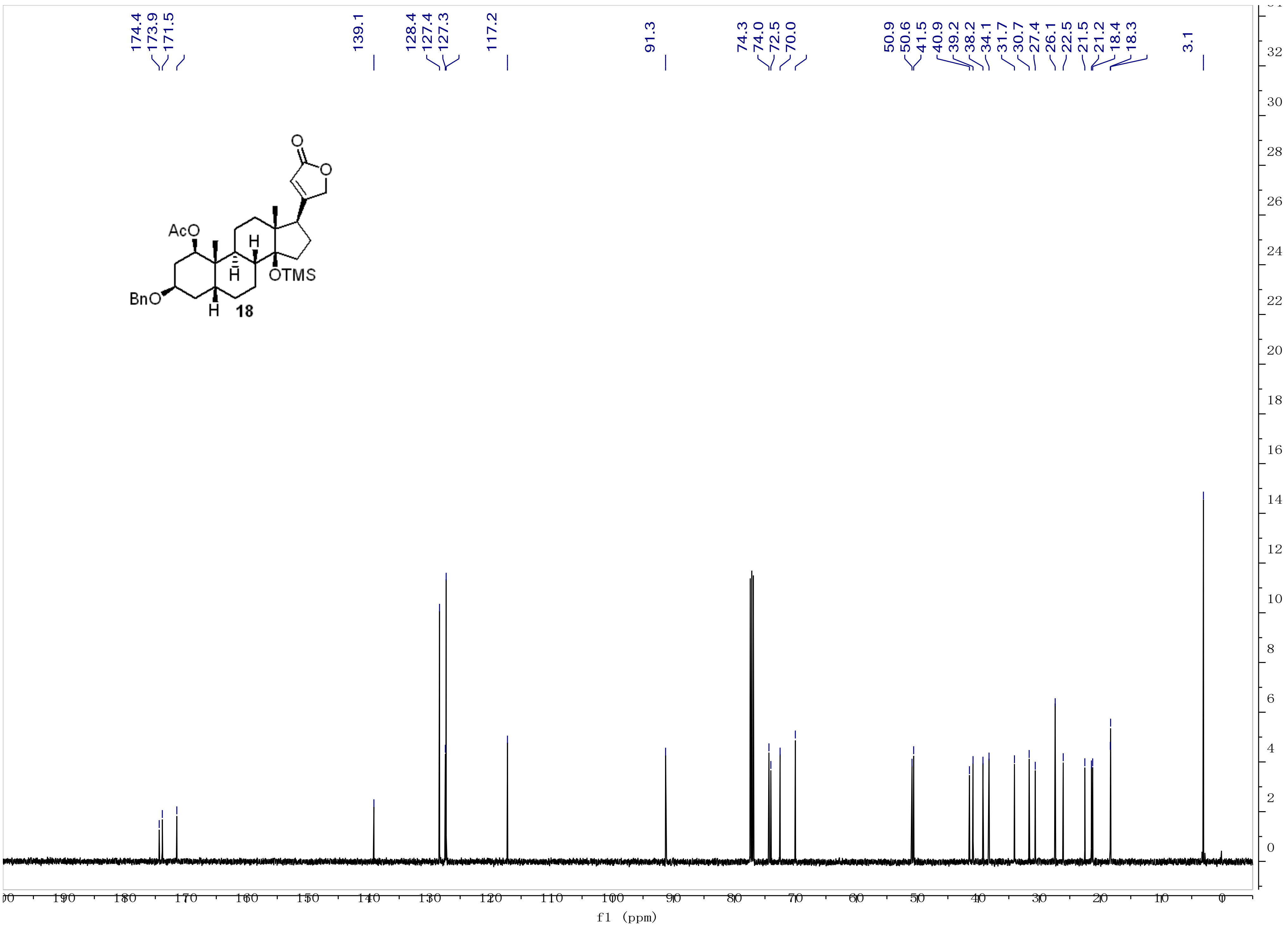

Figure S28 ^{13}C NMR spectrum of compound **18** ($CDCl_3$, 125 MHz)

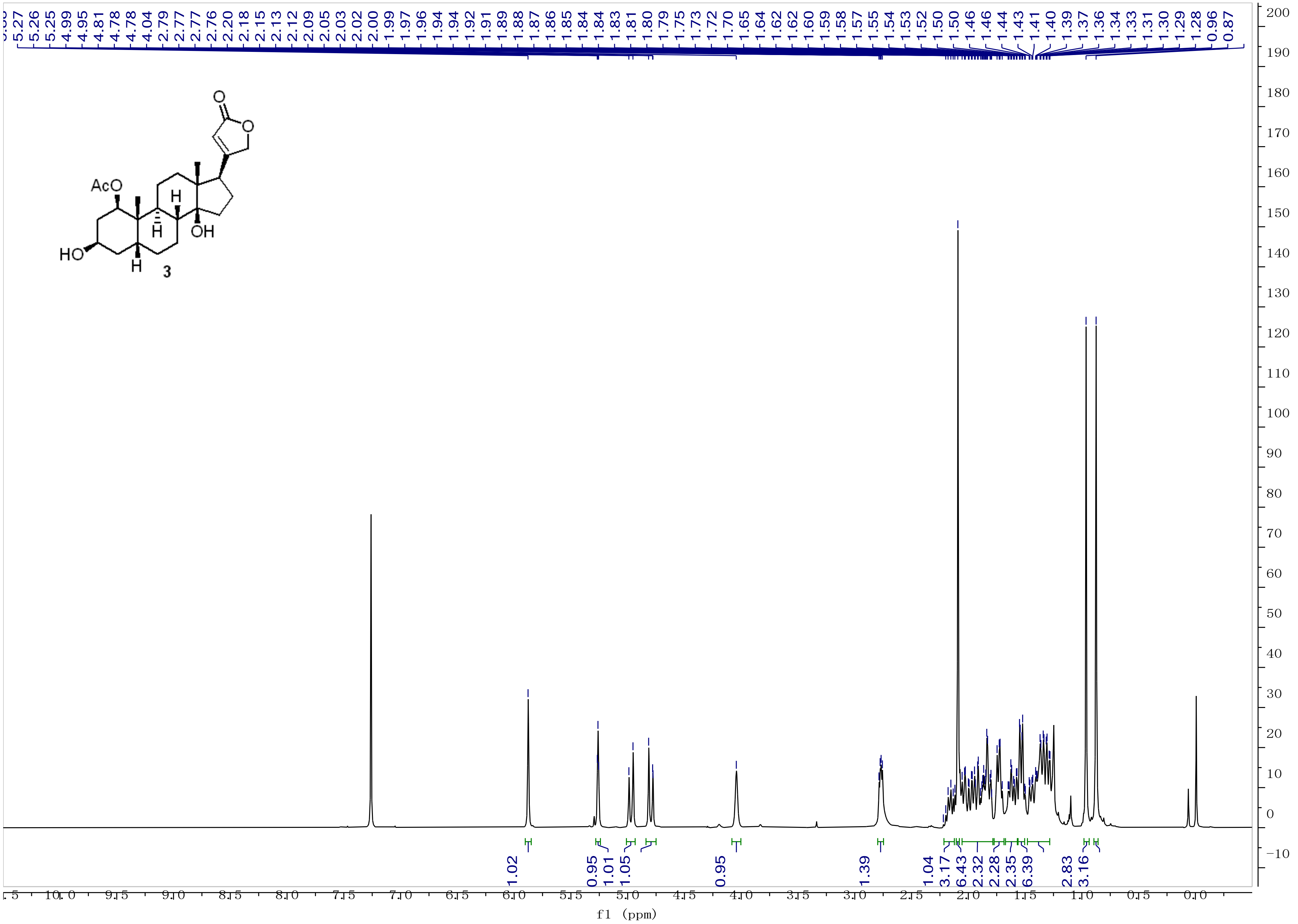

Figure S29 ^{1}H NMR spectrum of compound **3** ($CDCl_3$, 500 MHz)

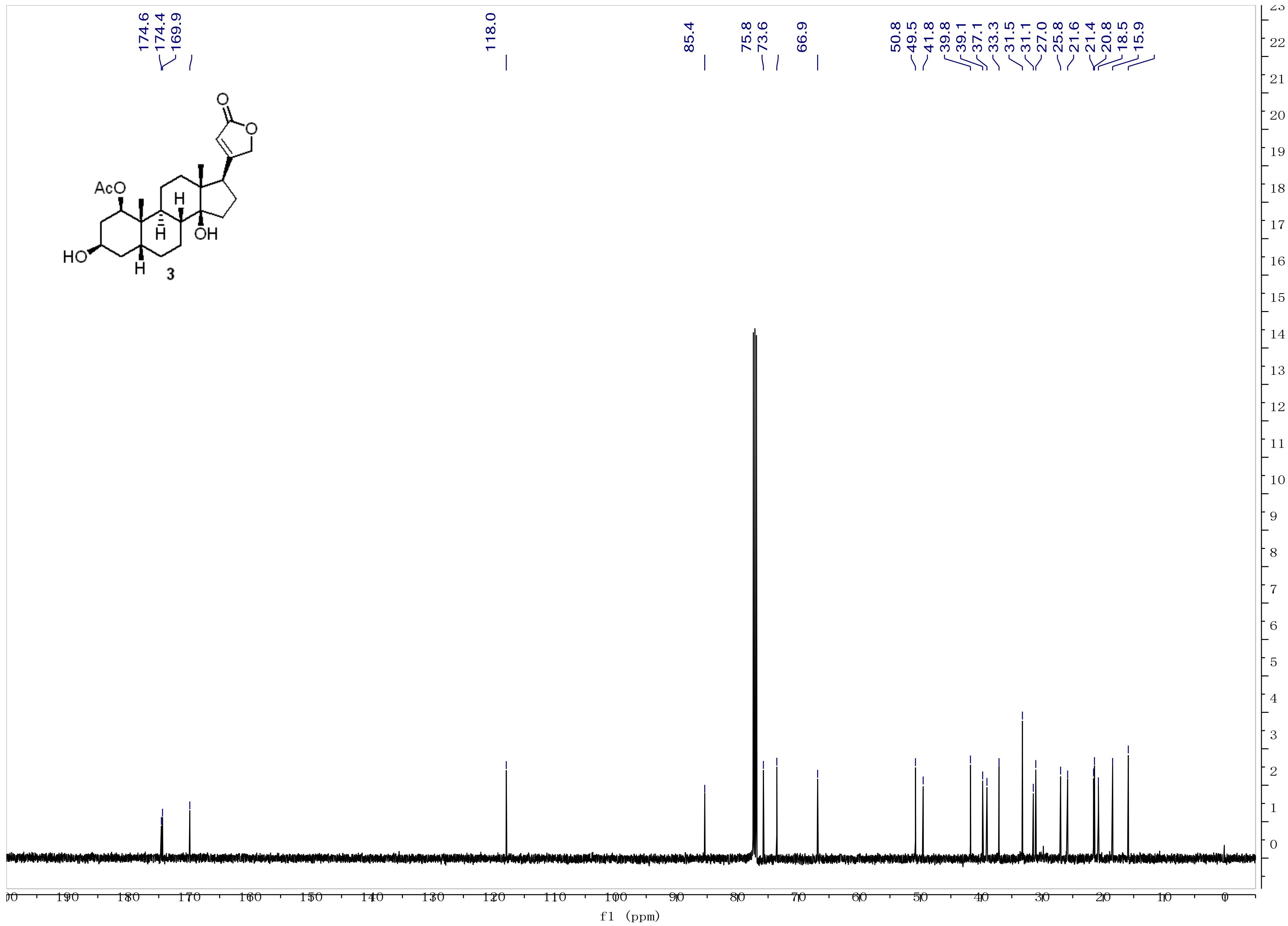

Figure S30 ^{13}C NMR spectrum of compound **3** ($CDCl_3$, 125 MHz)

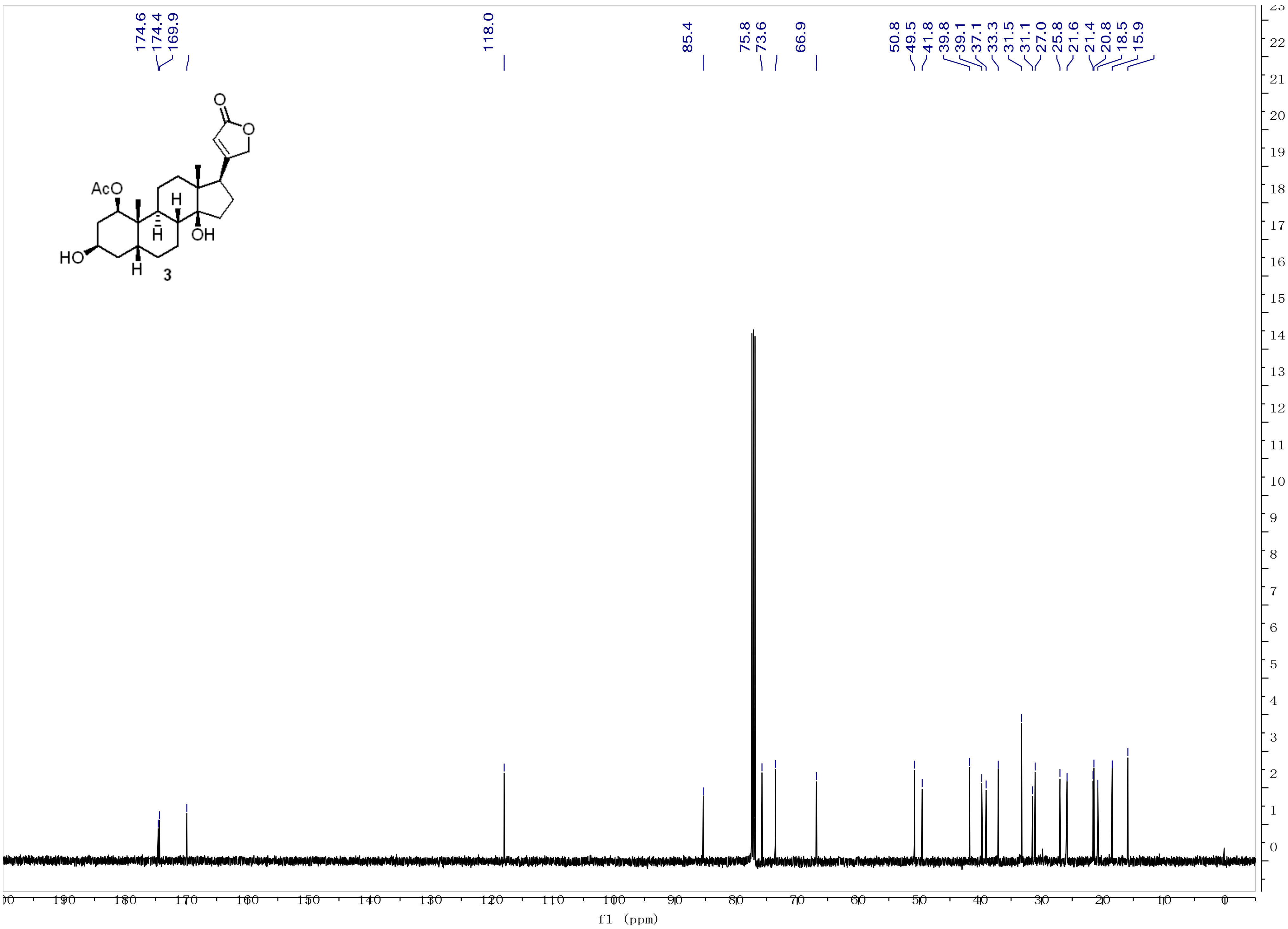

Figure S31 COSY spectrum of compound **3** ($CDCl_3$, 500 MHz)

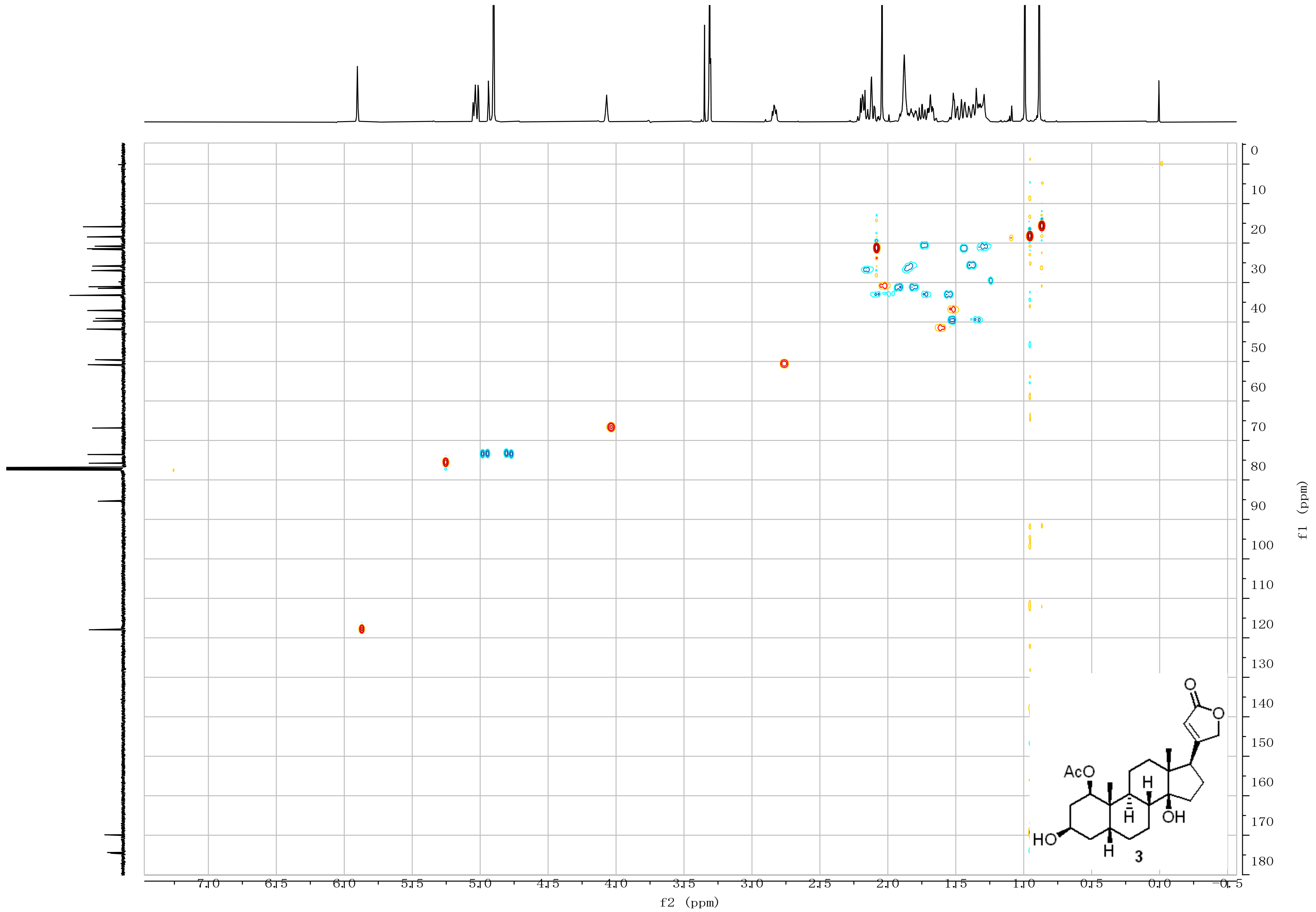

Figure S32 HSQC spectrum of compound **3** ($CDCl_3$, 500 MHz)

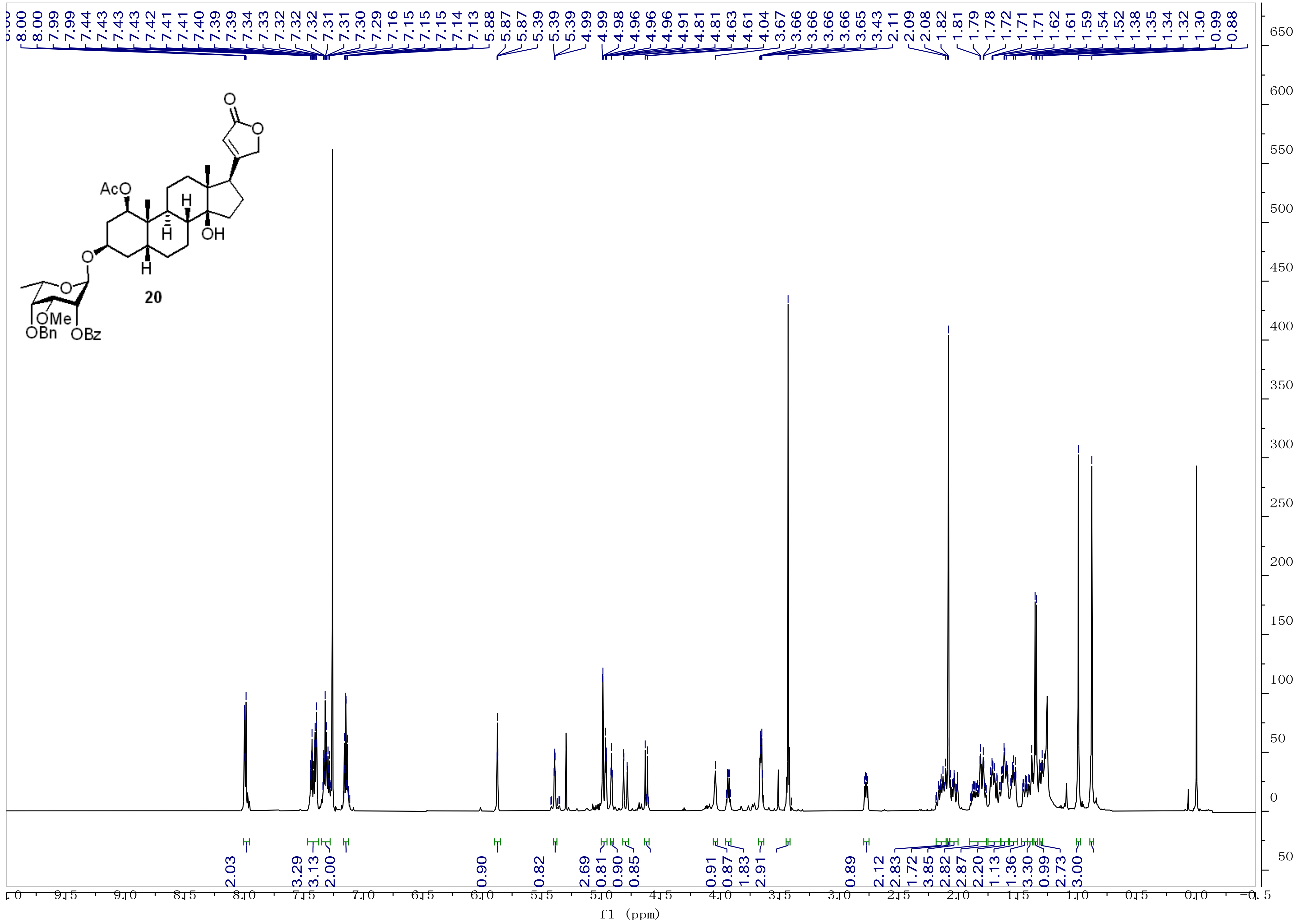

Figure S33 ^{1}H NMR spectrum of compound **20** ($CDCl_3$, 600 MHz)

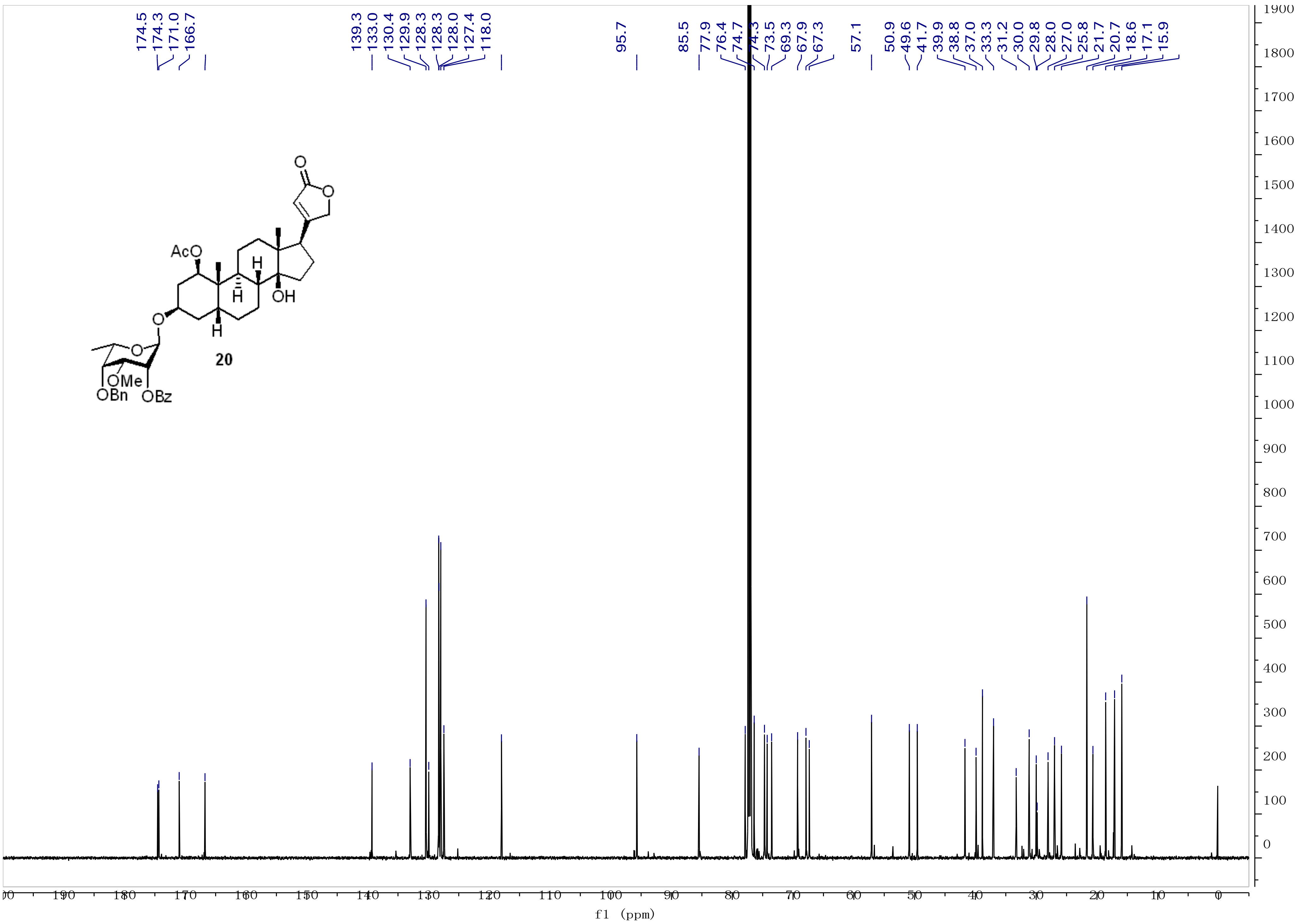

Figure S34 ^{13}C NMR spectrum of compound **20** ($CDCl_3$, 150 MHz)

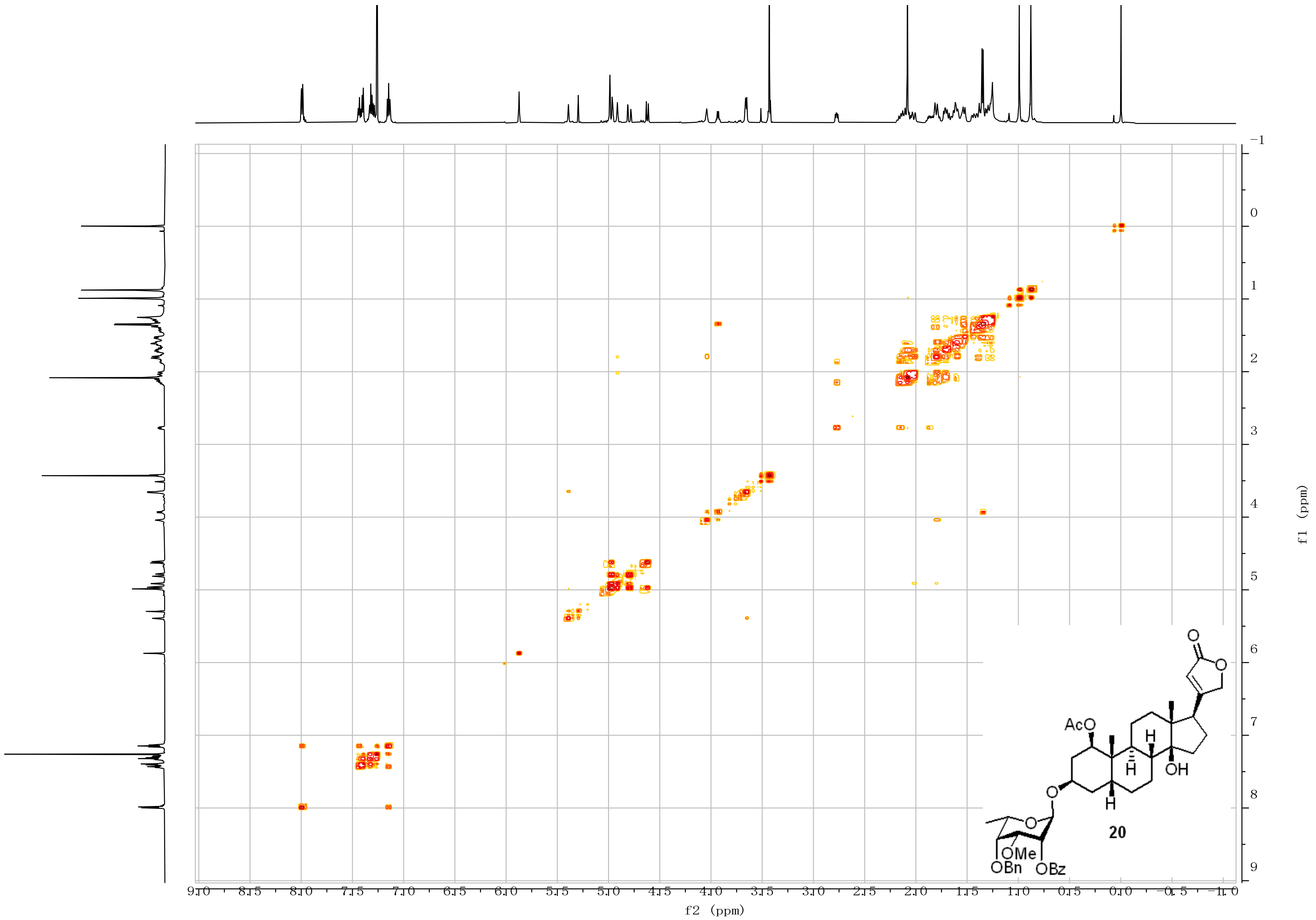

Figure S35 COSY spectrum of compound **20** ($CDCl_3$, 600 MHz)

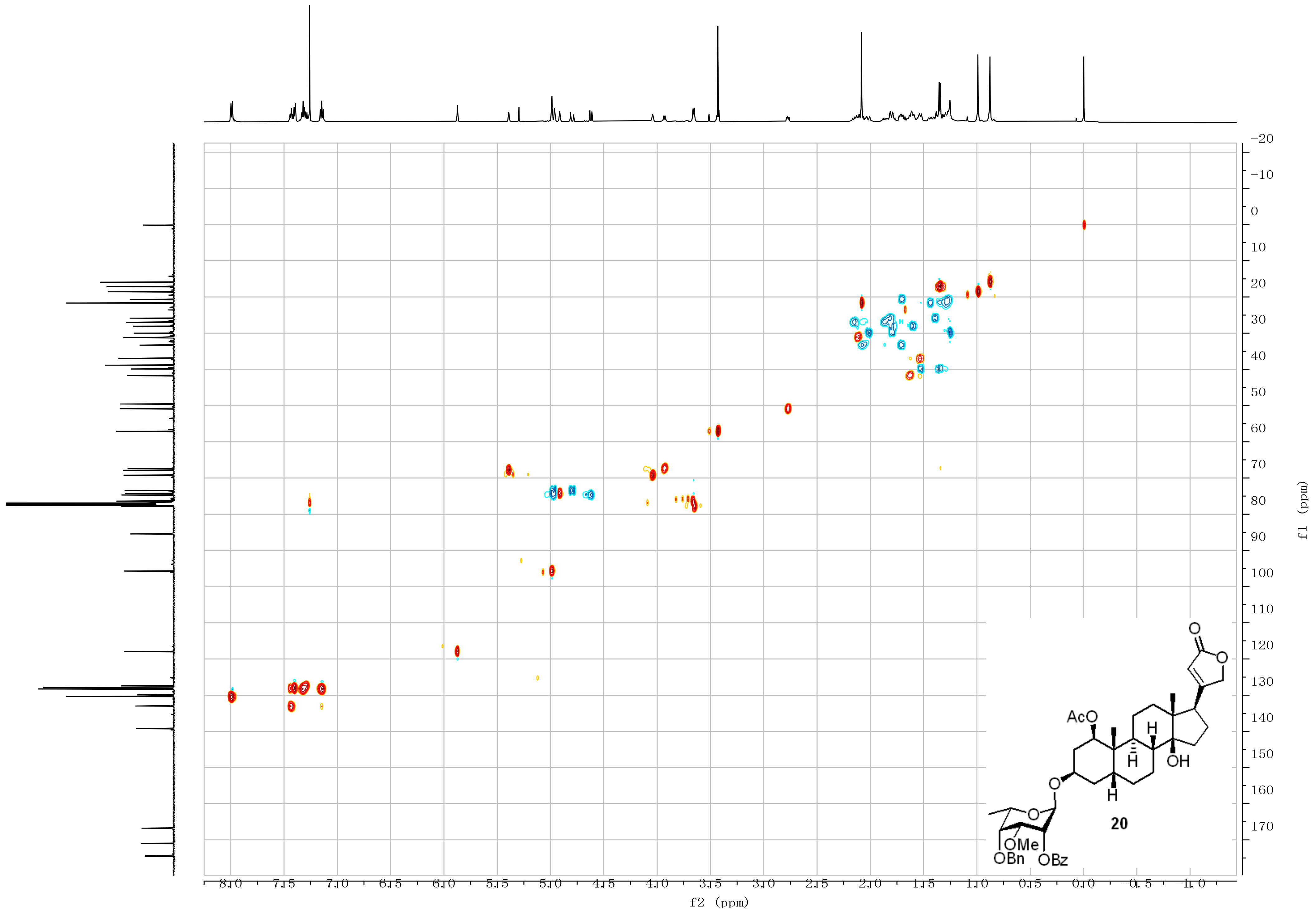

Figure S36 HSQC spectrum of compound **20** ($CDCl_3$, 600 MHz)

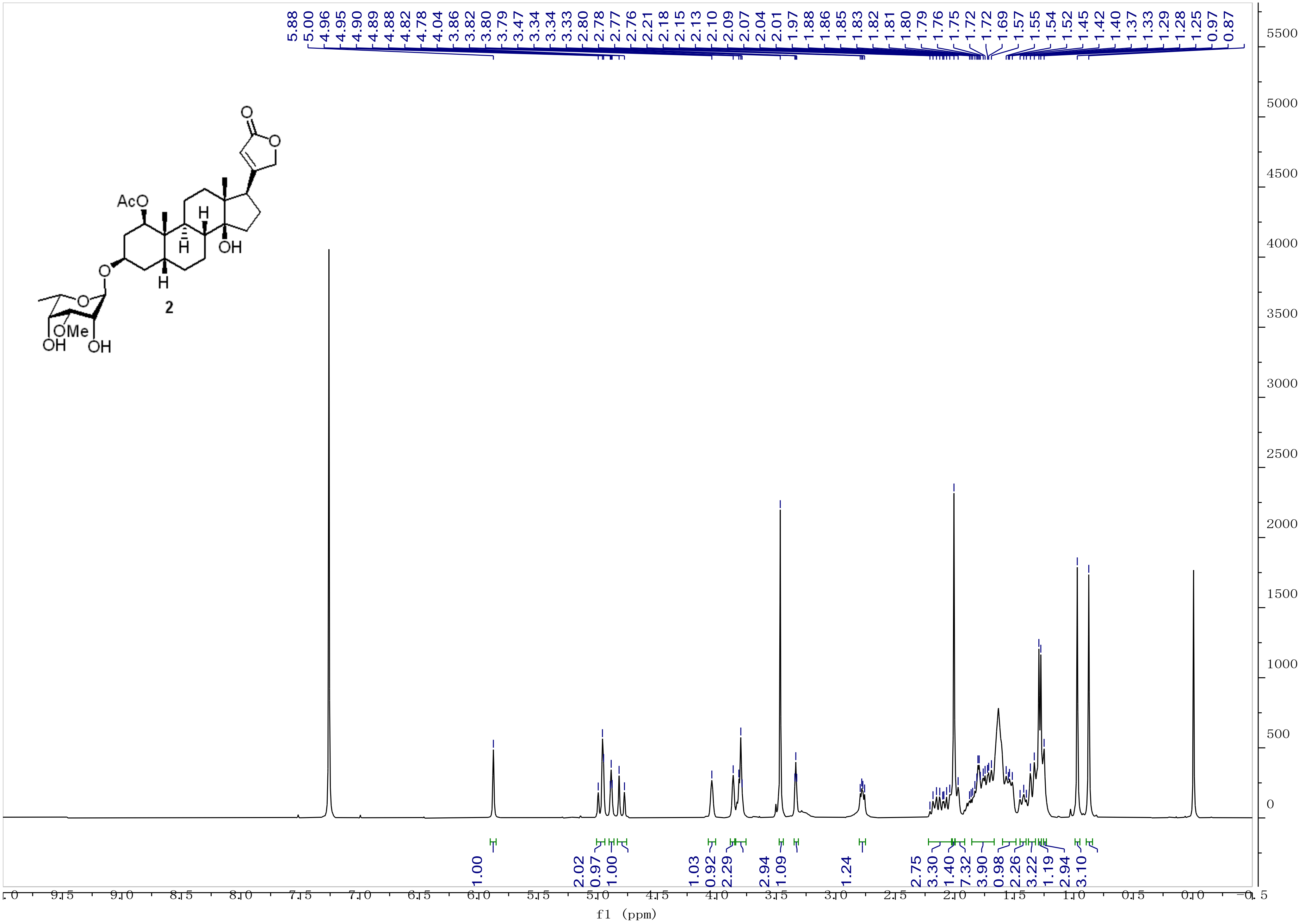

Figure S37 ^{1}H NMR spectrum of compound **2** ($CDCl_3$, 400 MHz)

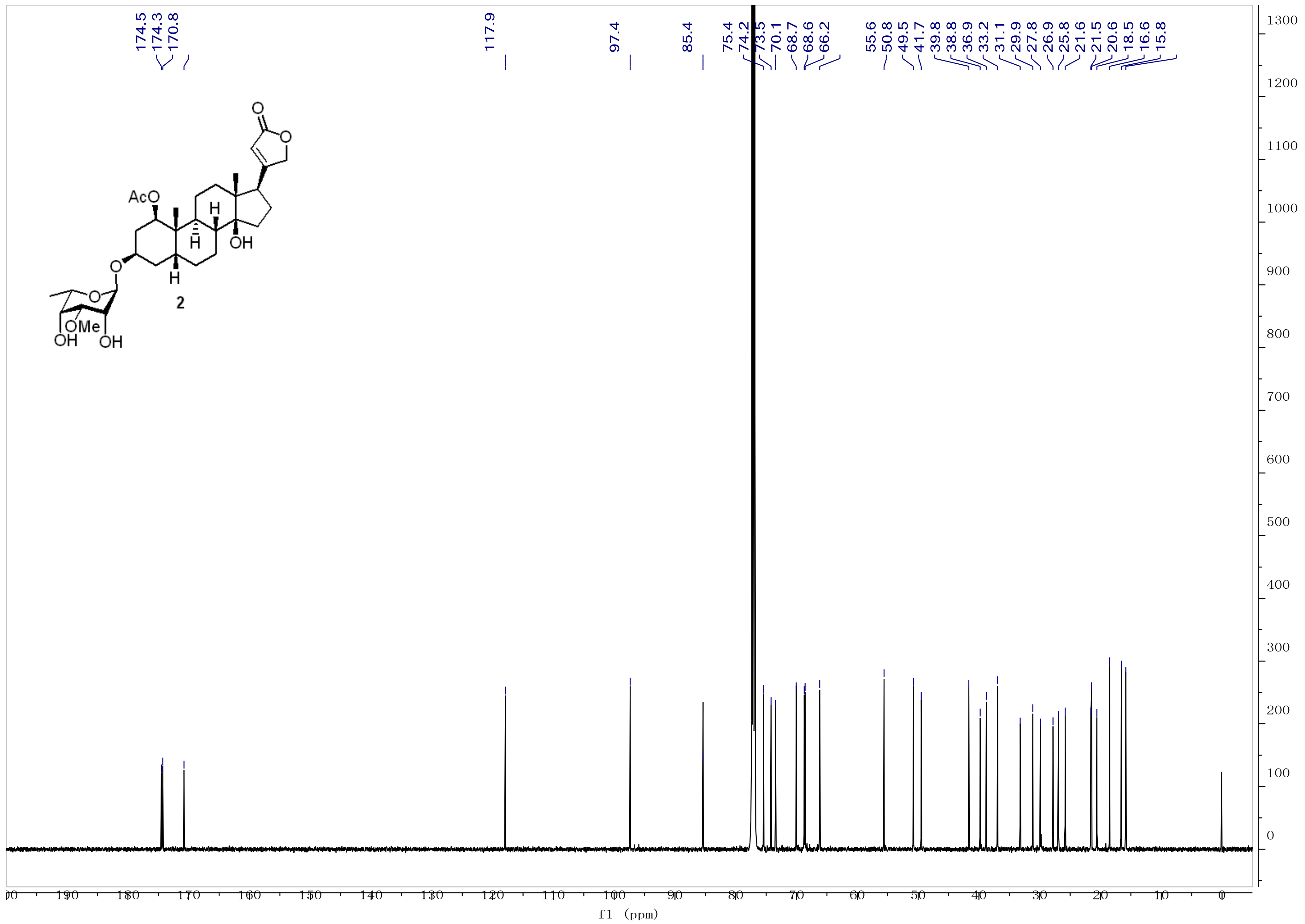

Figure S38 ^{13}C NMR spectrum of compound **2** ($CDCl_3$, 150 MHz)

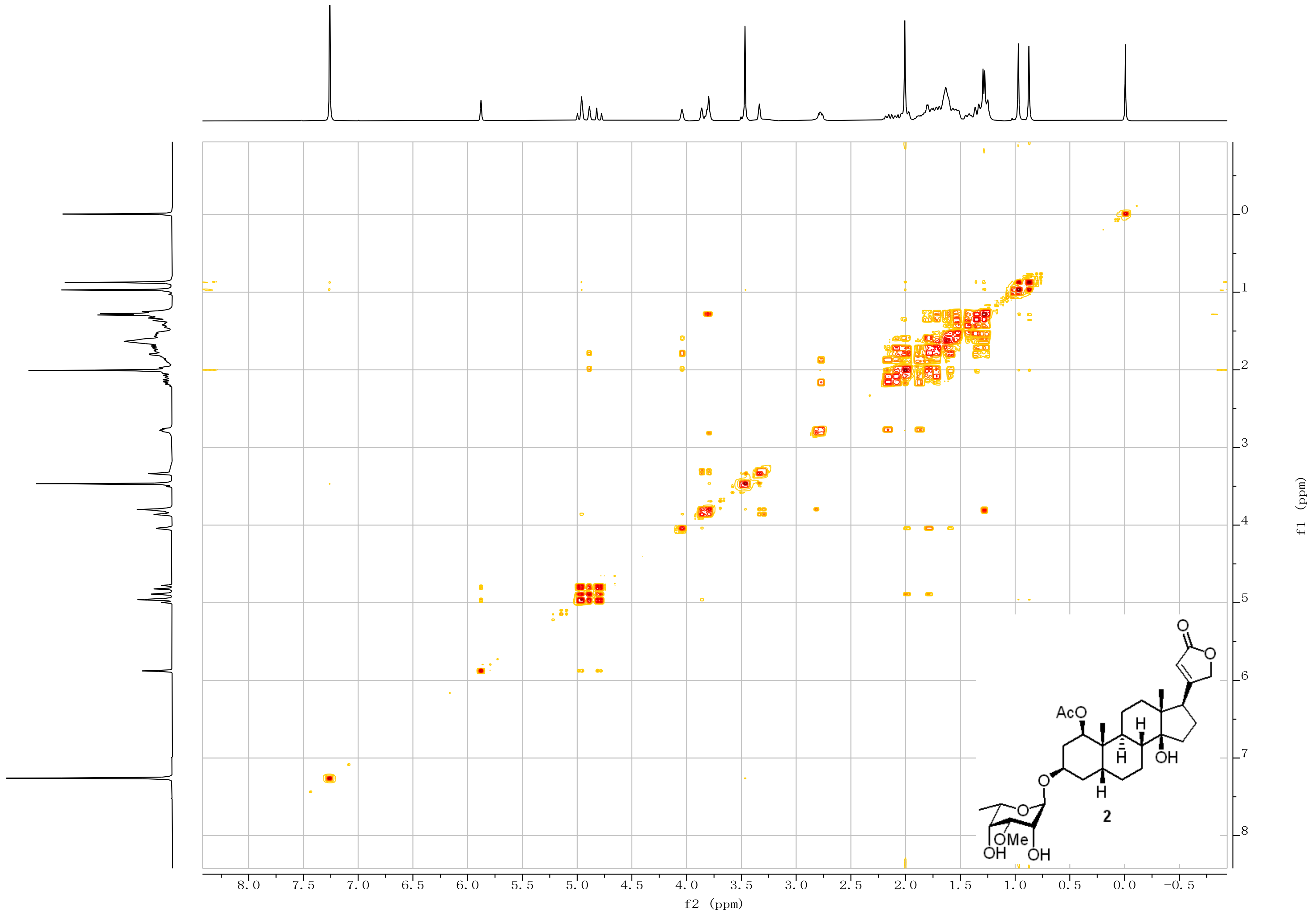

Figure S39 COSY spectrum of compound **2** ($CDCl_3$, 600 MHz)

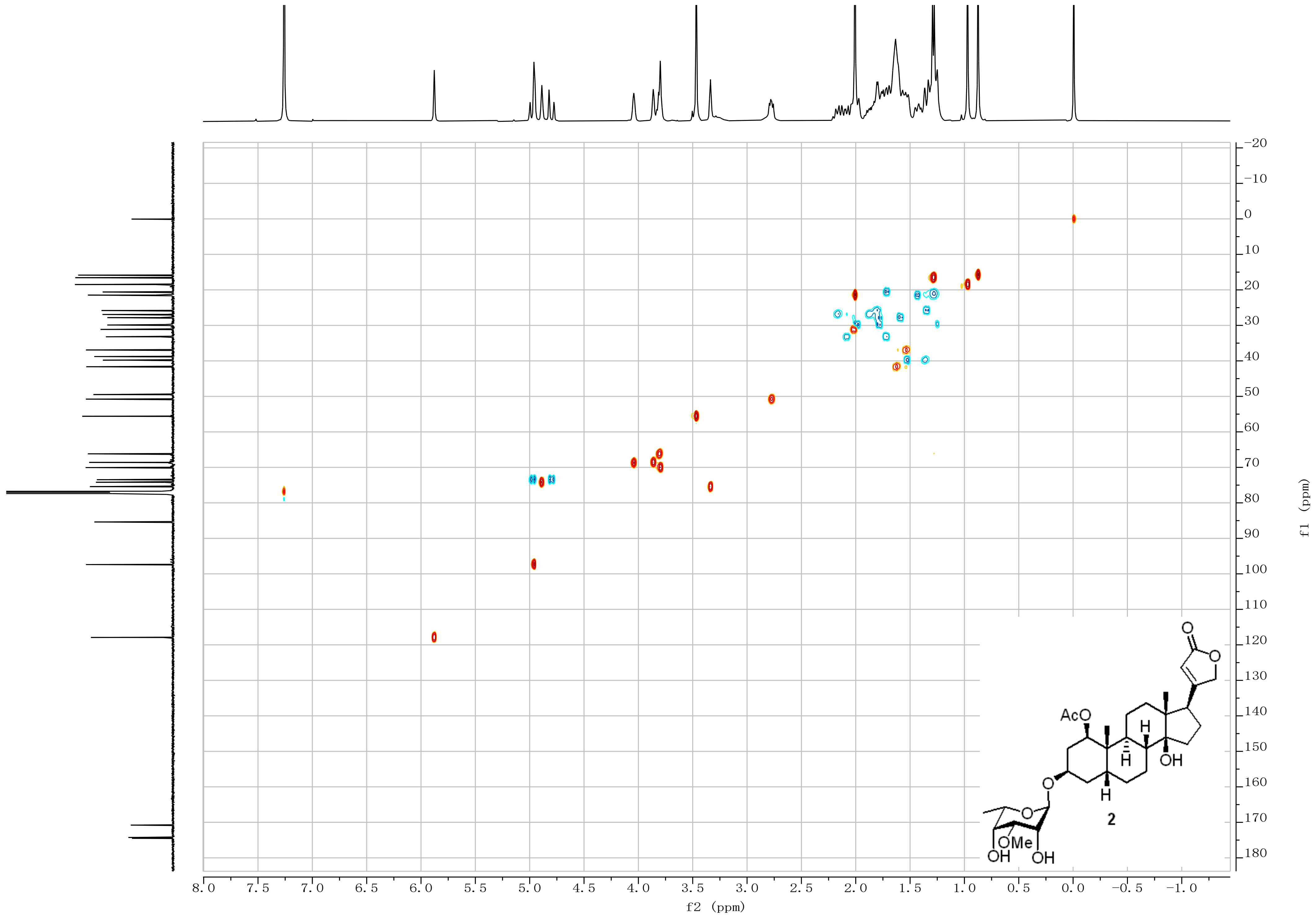

Figure S40 HSQC spectrum of compound **2** ($CDCl_3$, 600 MHz)

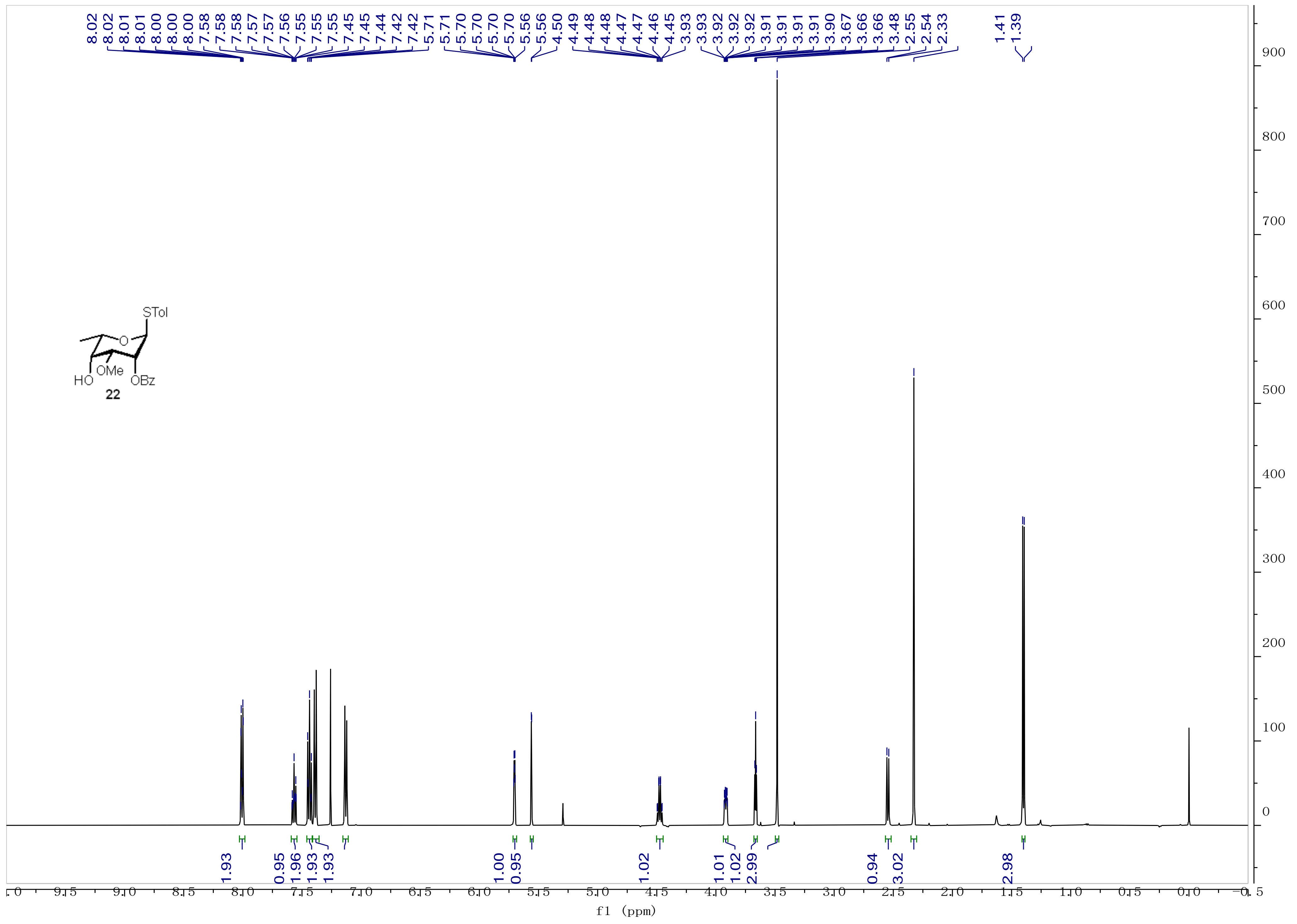

Figure S41 ^{1}H NMR spectrum of compound **22** ($CDCl_3$, 500 MHz)

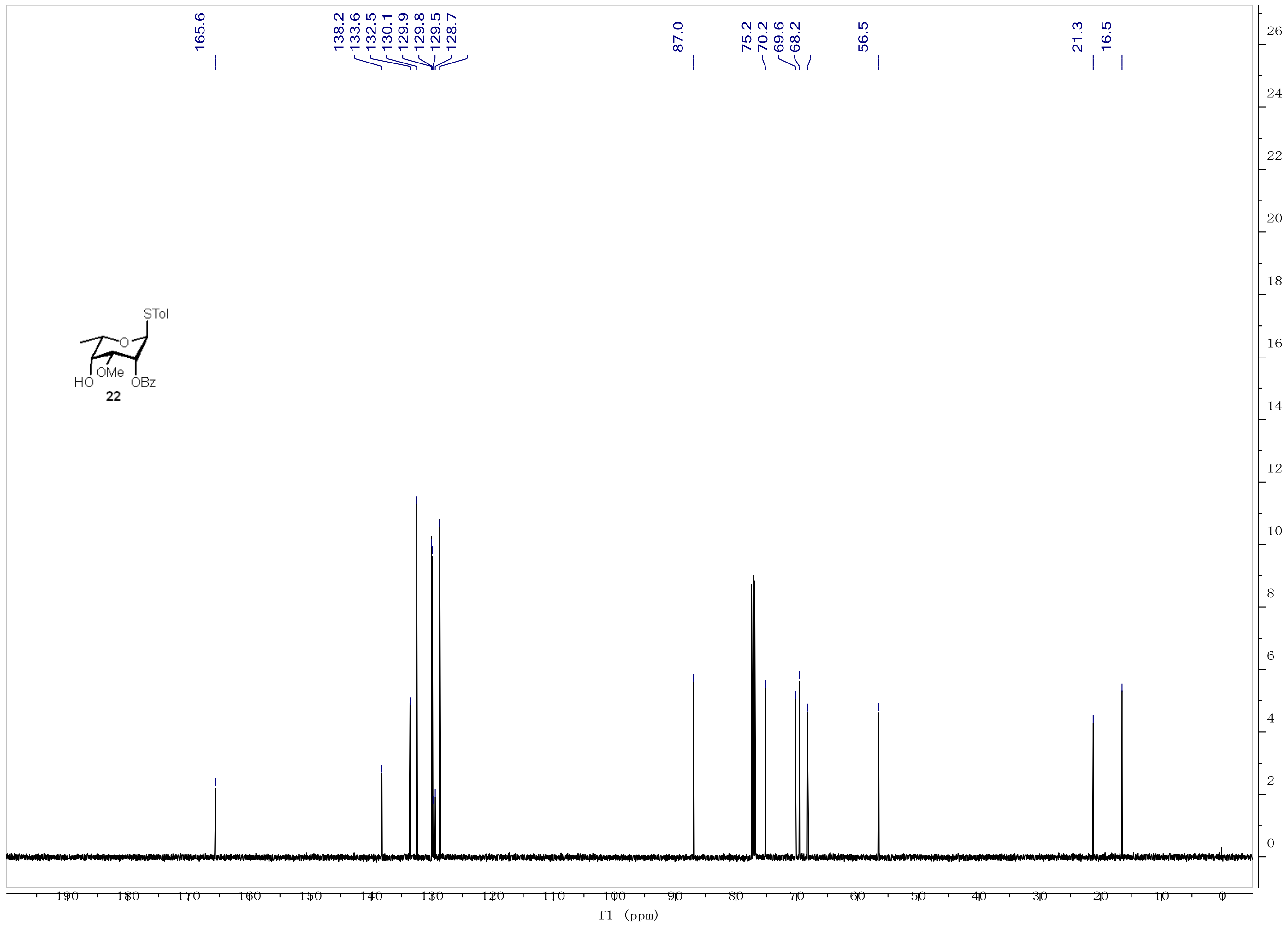

Figure S42 ^{13}C NMR spectrum of compound **22** ($CDCl_3$, 125 MHz)

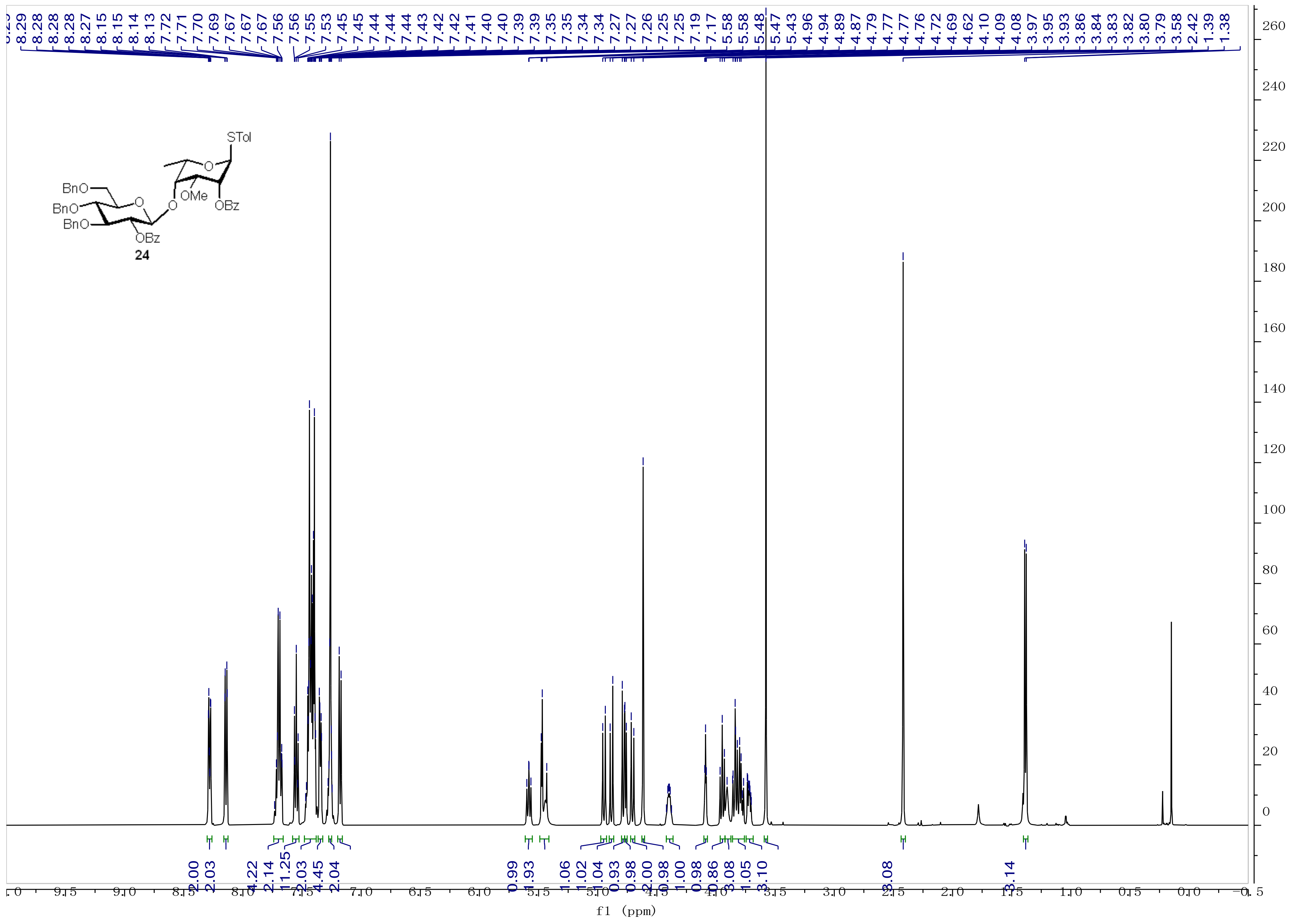

Figure S43 ^{1}H NMR spectrum of compound **24** ($CDCl_3$, 500 MHz)

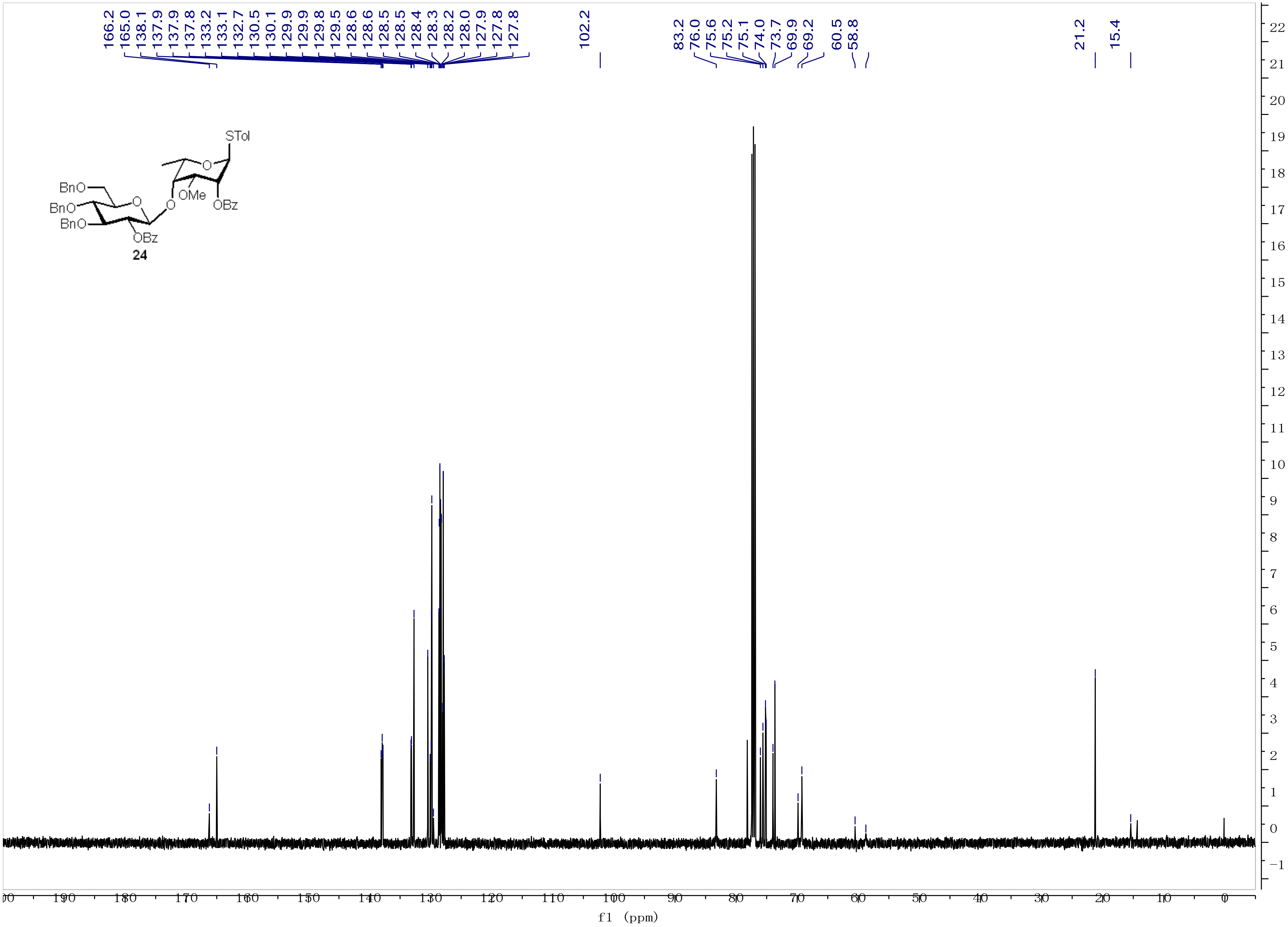

Figure S44 ^{13}C NMR spectrum of compound **24** ($CDCl_3$, 125 MHz)

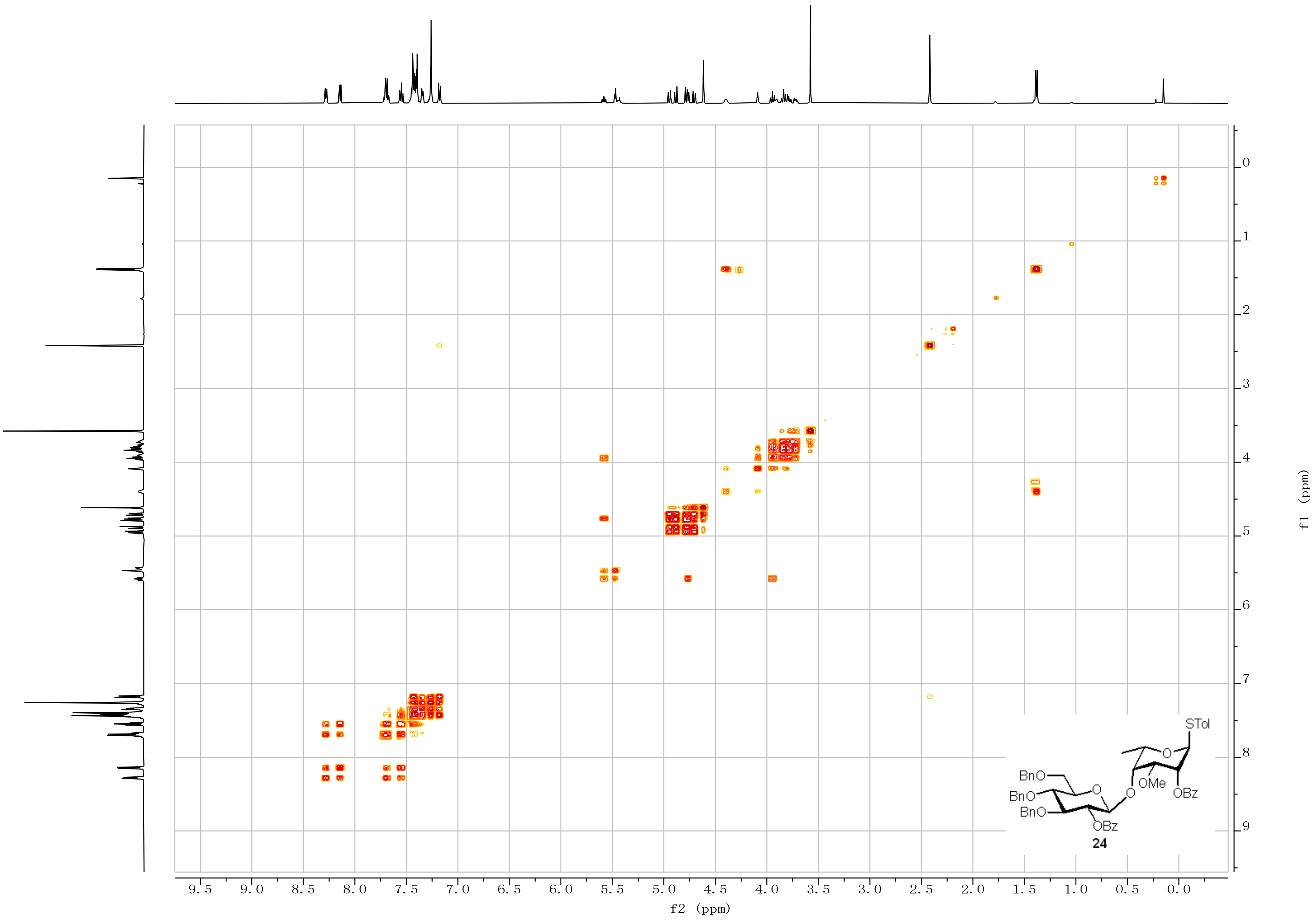

Figure S45 COSY spectrum of compound **24** ($CDCl_3$, 500 MHz)

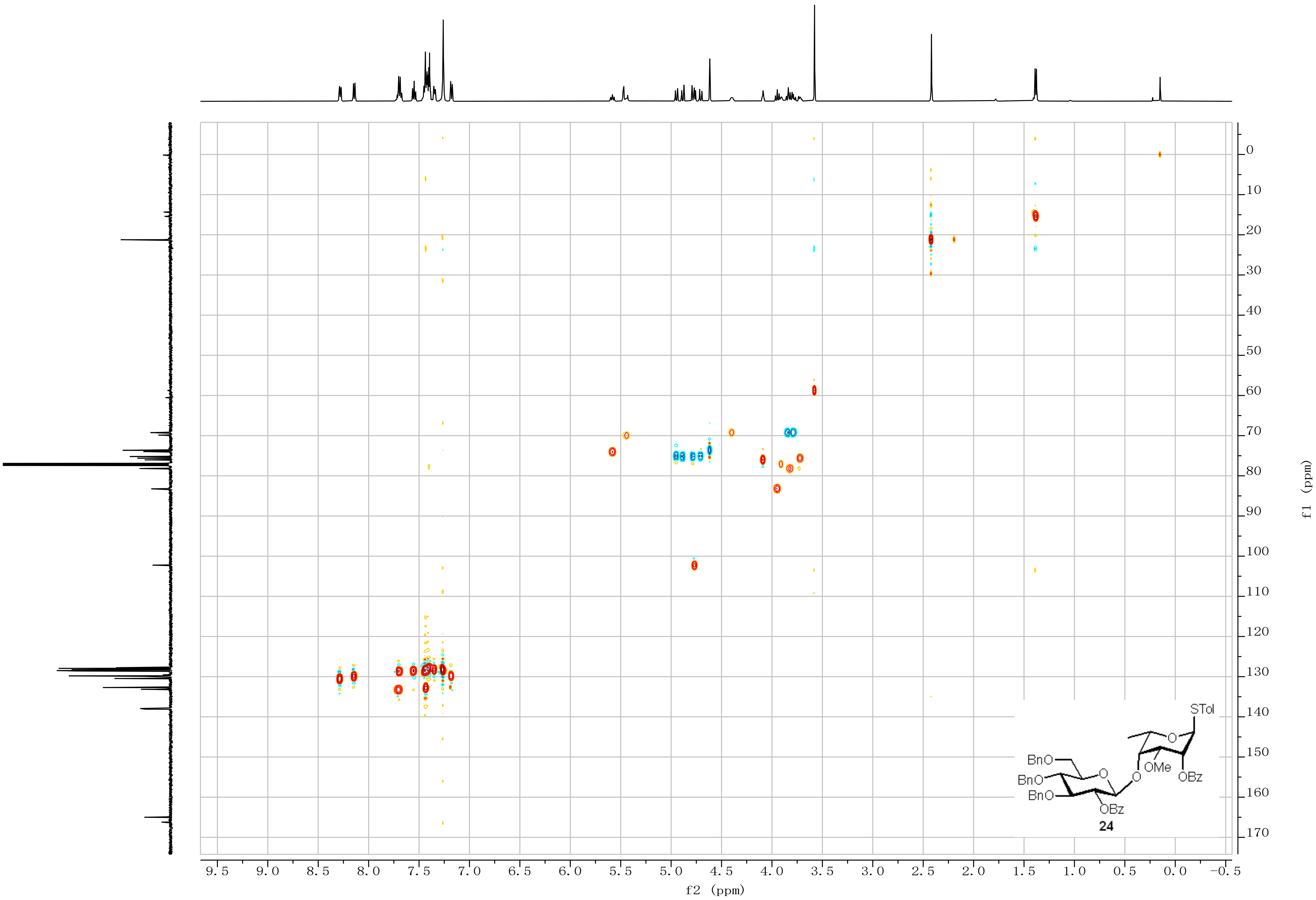

Figure S46 HSQC spectrum of compound **24** ($CDCl_3$, 500 MHz)

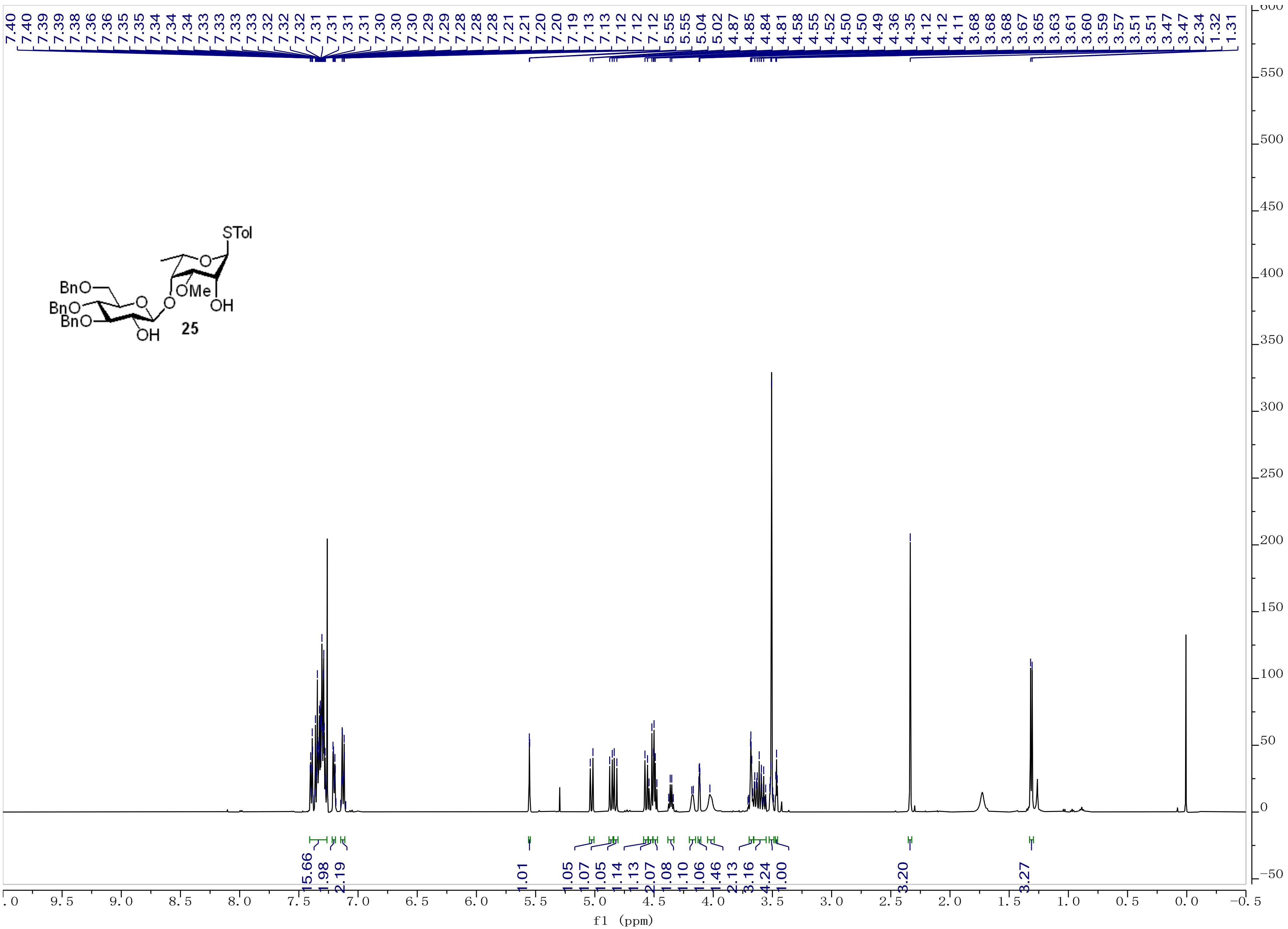

Figure S47 ^{1}H NMR spectrum of compound **25** ($CDCl_3$, 500 MHz)

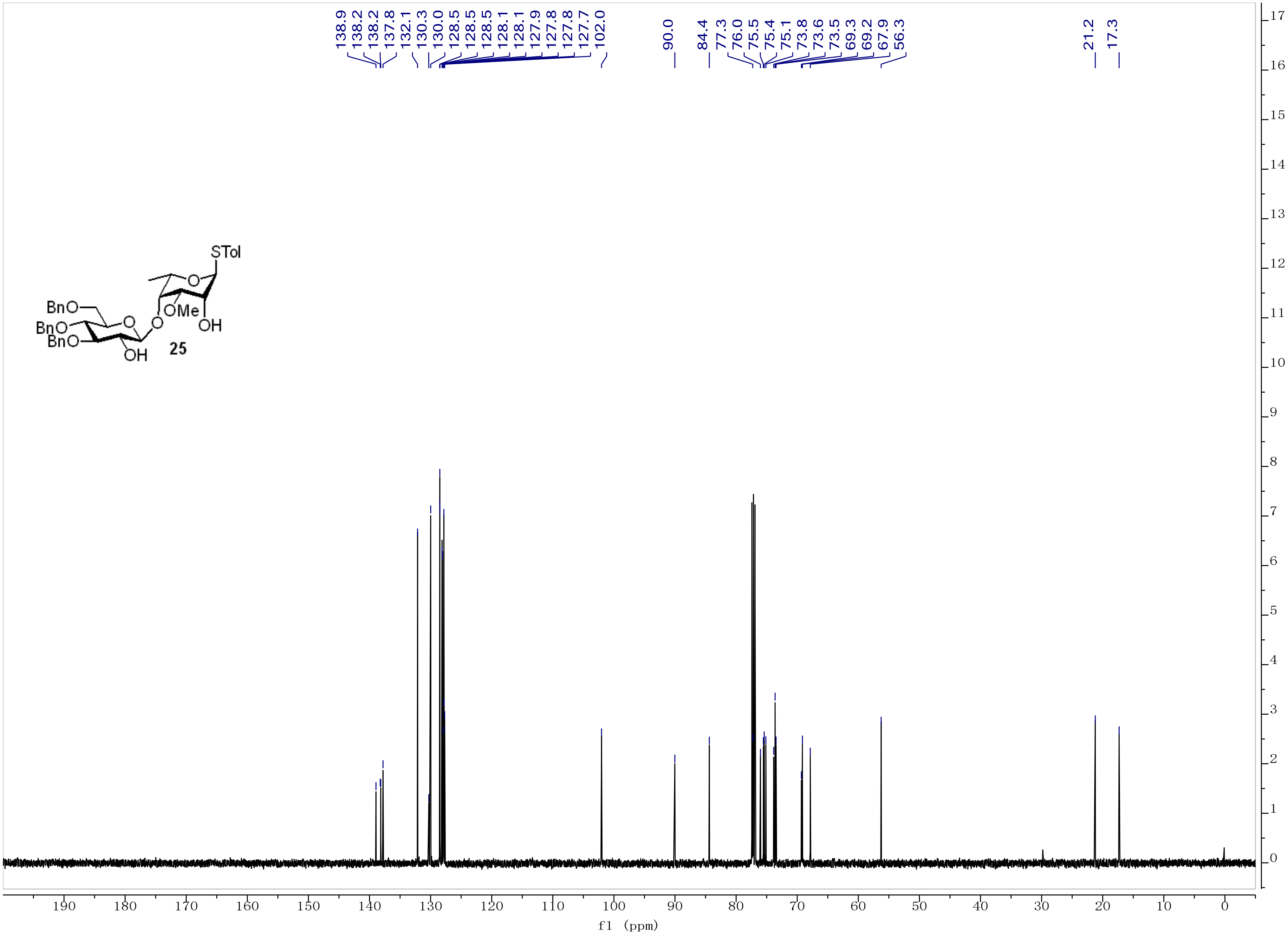

Figure S48 ^{13}C NMR spectrum of compound **25** ($CDCl_3$, 125 MHz)

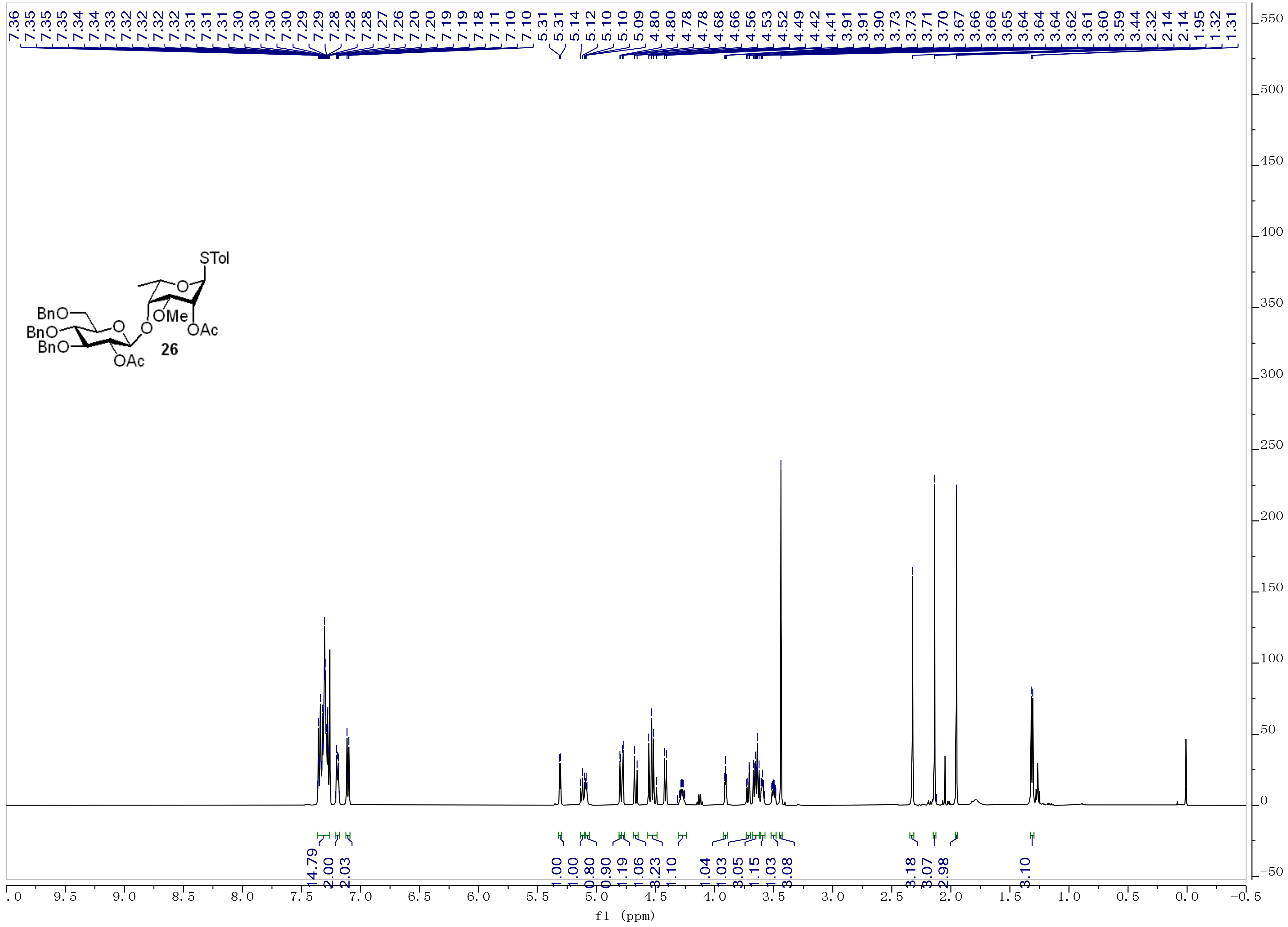

Figure S49 ^{1}H NMR spectrum of compound **26** ($CDCl_3$, 500 MHz)

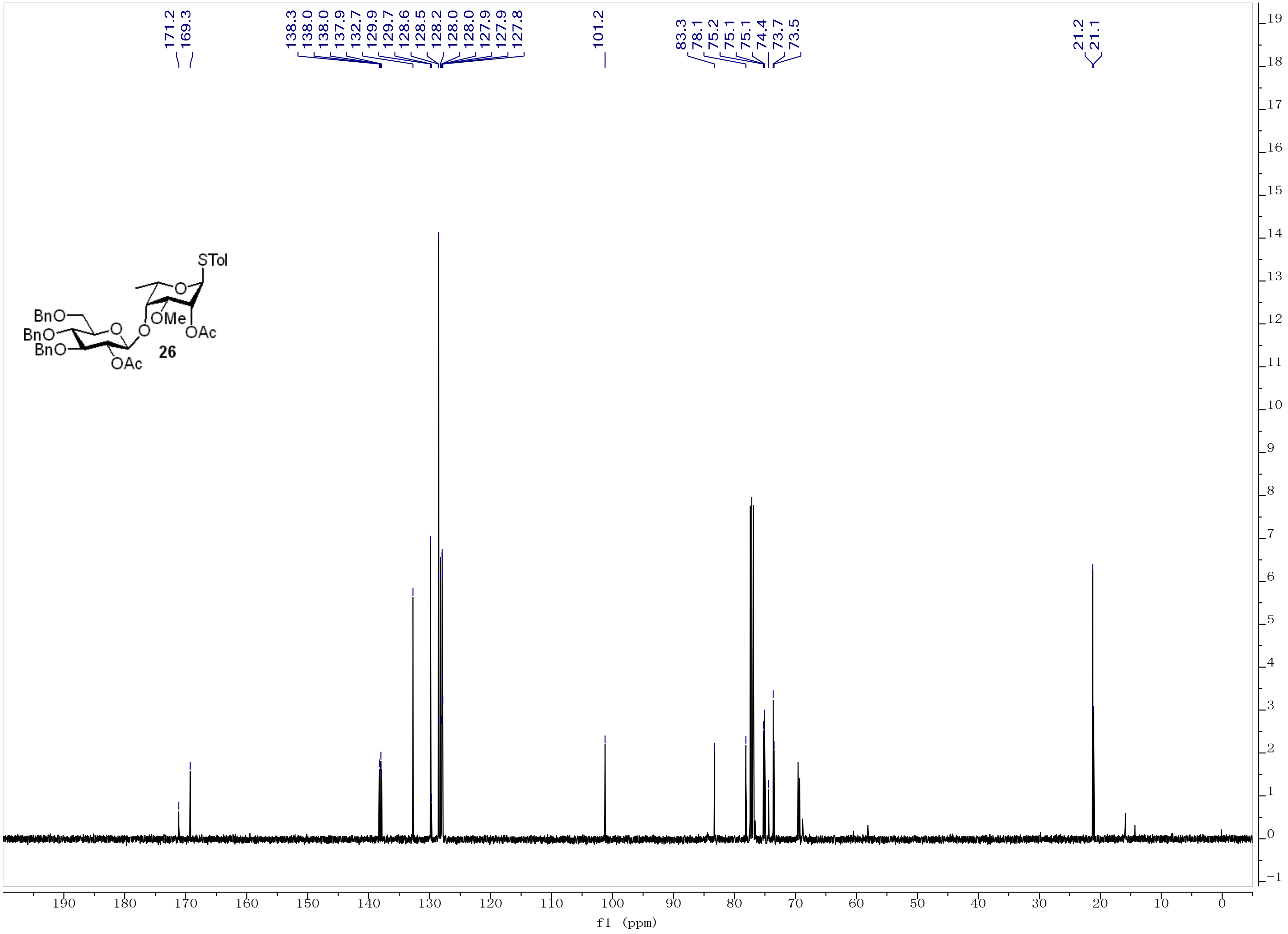

Figure S50 ^{13}C NMR spectrum of compound **26** ($CDCl_3$, 125 MHz)

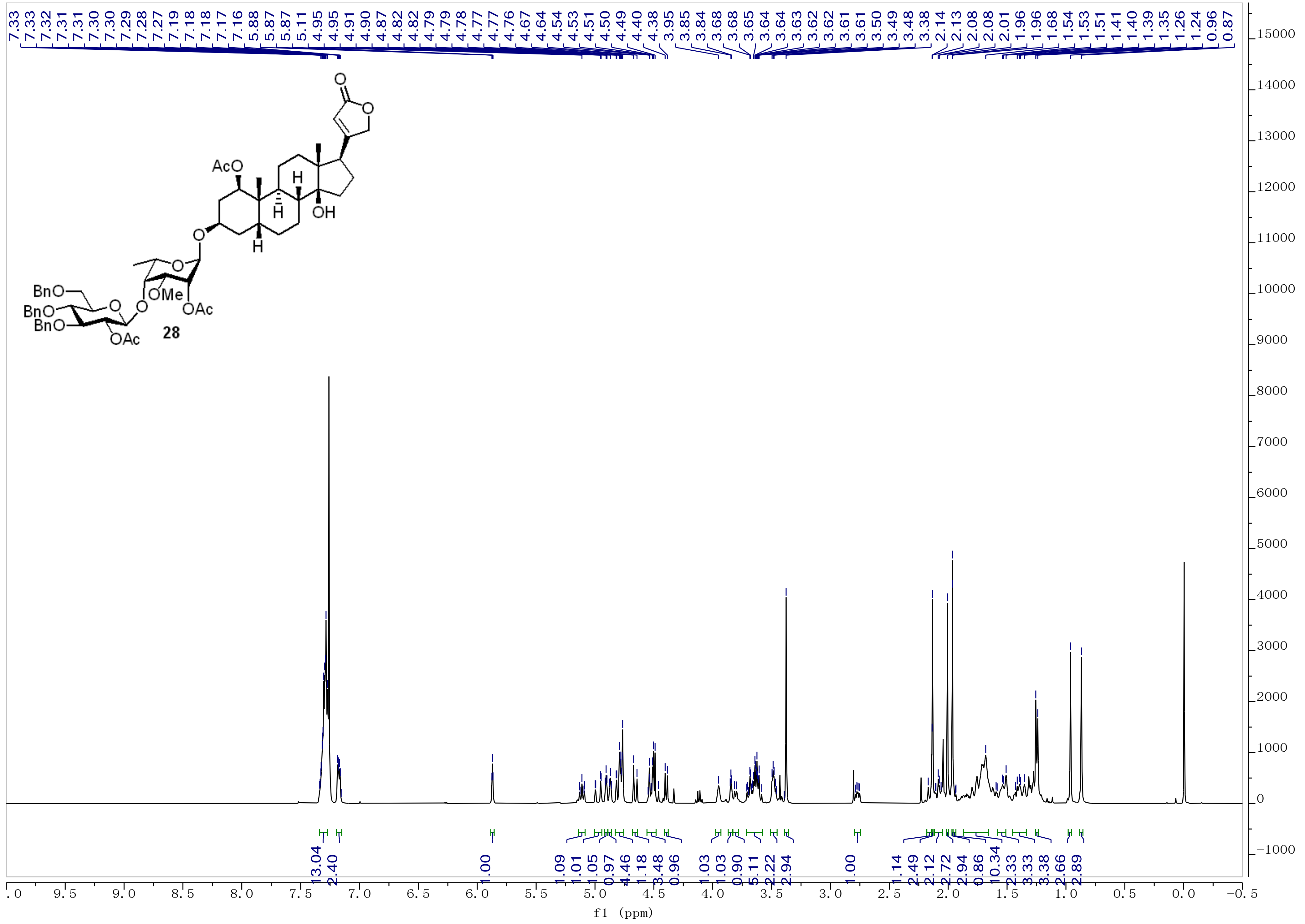

Figure S51 ^{1}H NMR spectrum of compound **28** ($CDCl_3$, 400 MHz)

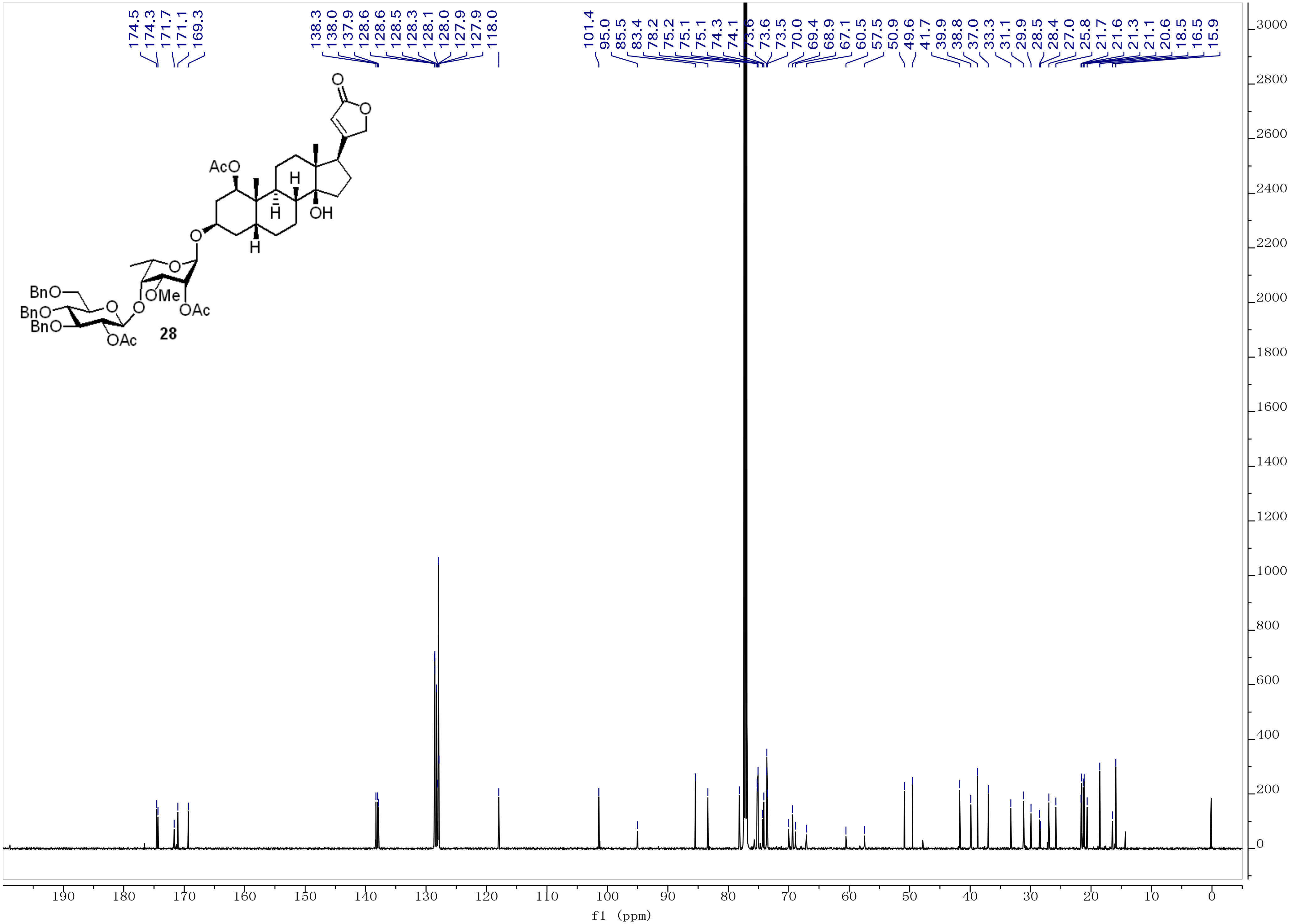

Figure S52 ^{13}C NMR spectrum of compound **28** ($CDCl_3$, 150 MHz)

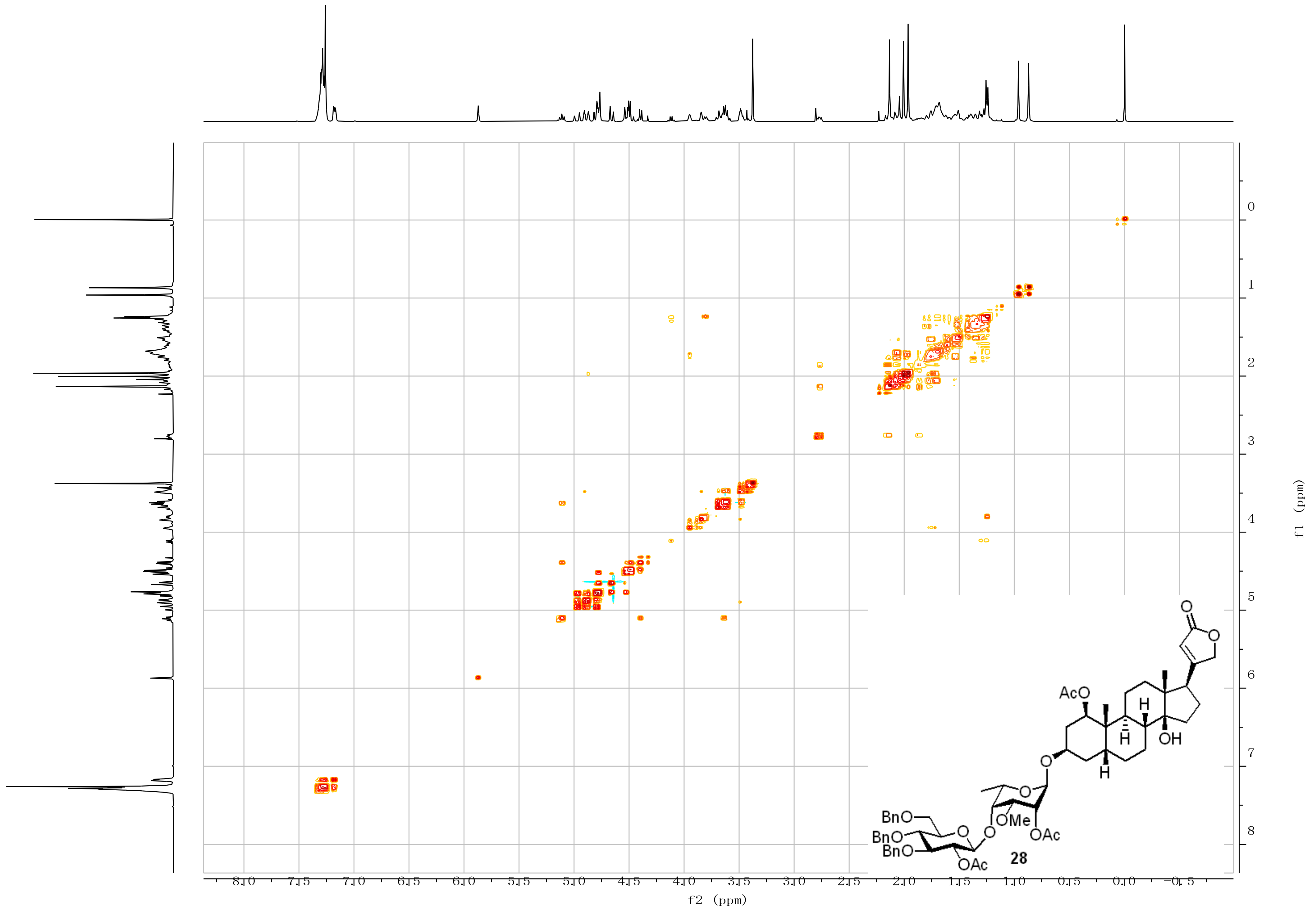

Figure S53 COSY spectrum of compound **28** ($CDCl_3$, 600 MHz)

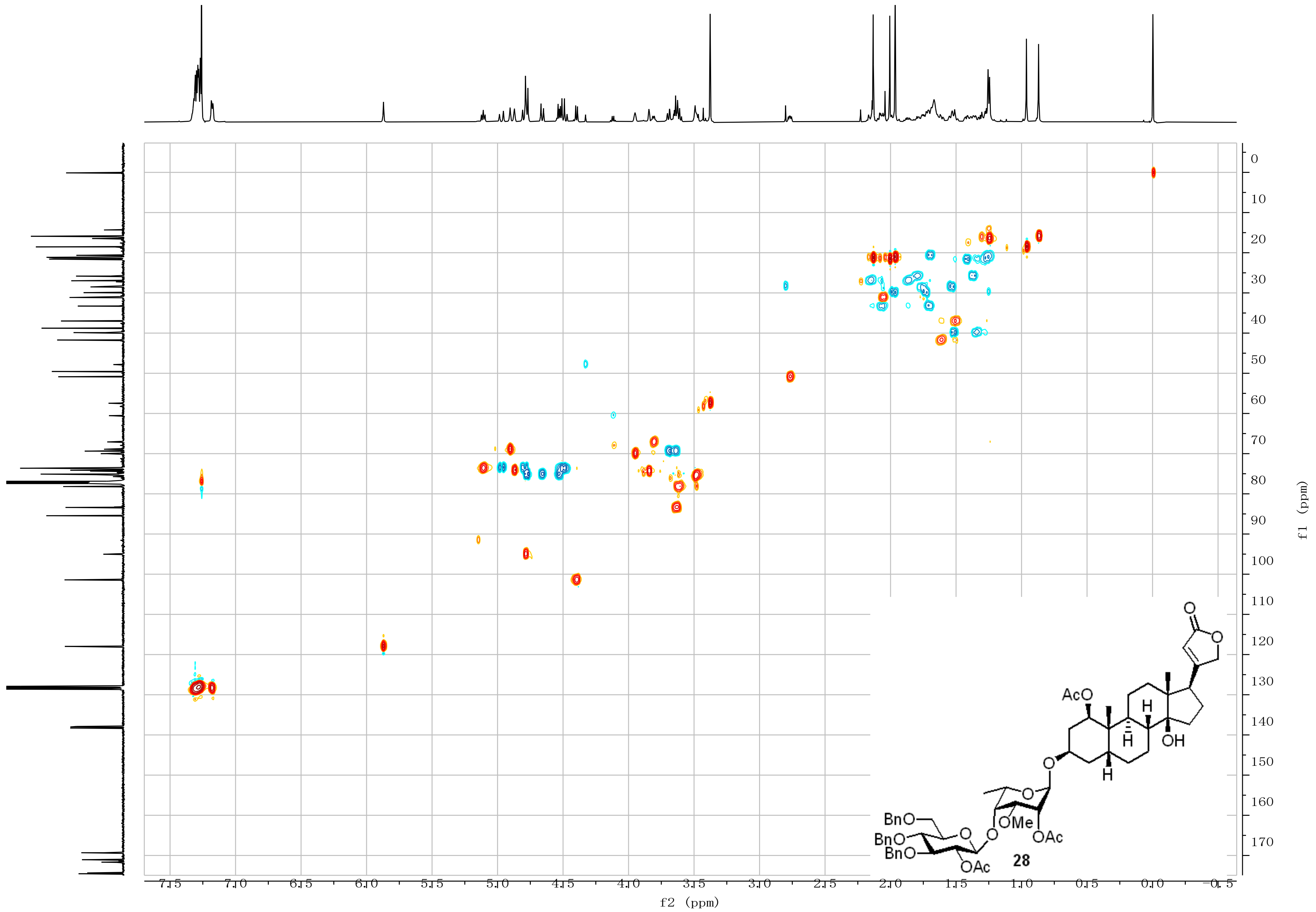

Figure S54 HSQC spectrum of compound **28** ($CDCl_3$, 600 MHz)

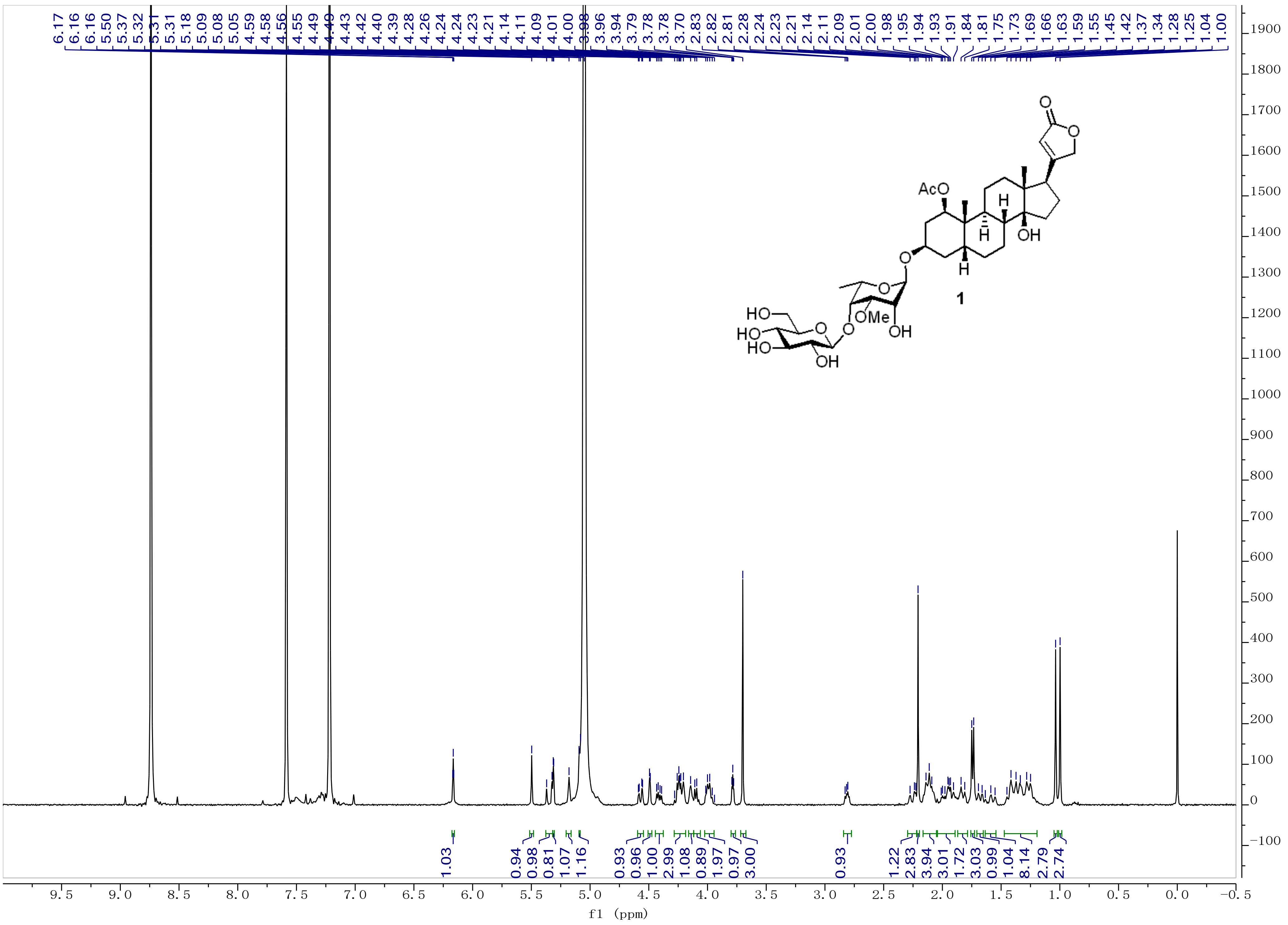

Figure S55 ^{1}H NMR spectrum of compound **1** (C_5D_5N, 400 MHz)

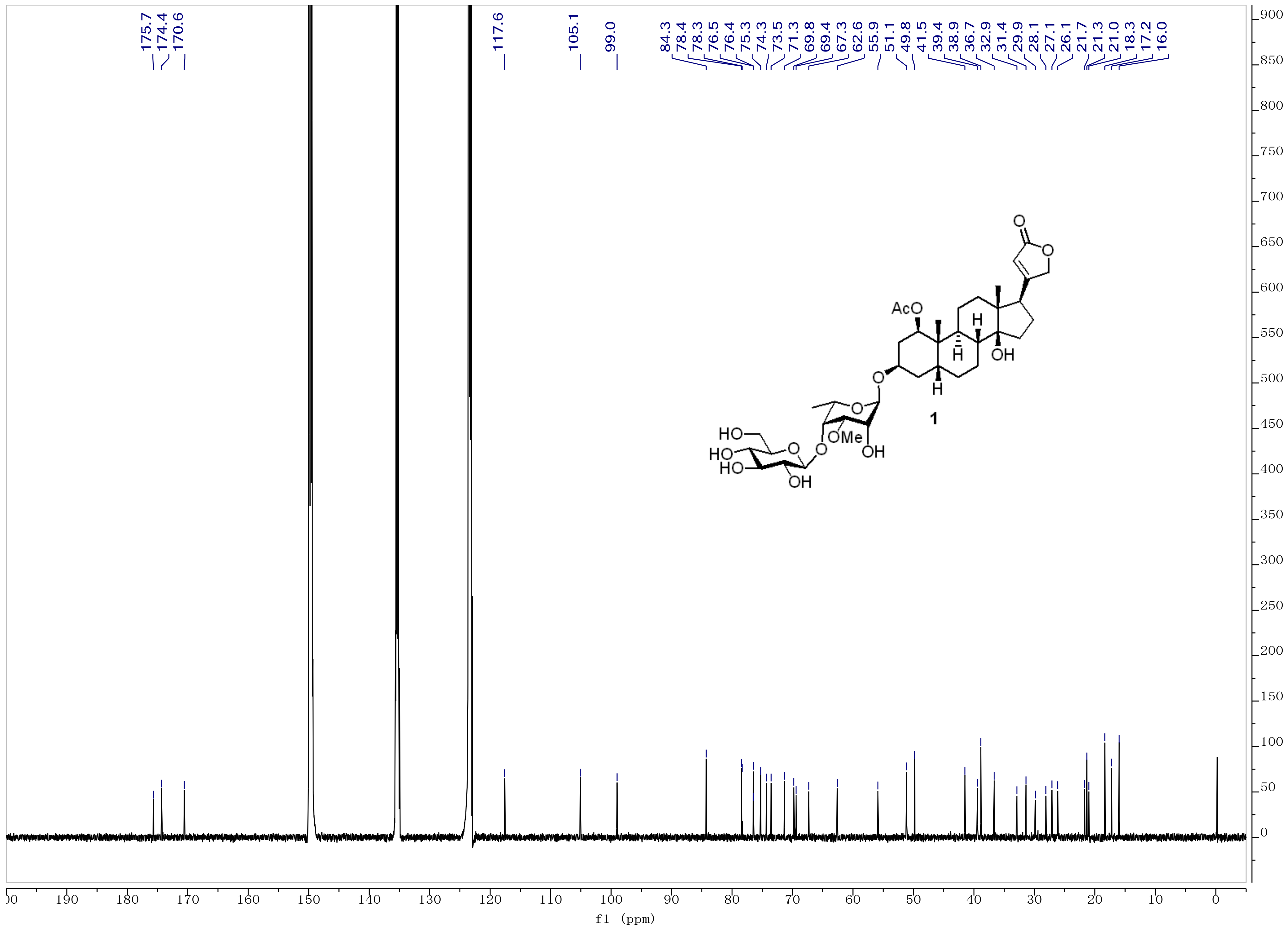

Figure S56 ^{13}C NMR spectrum of compound **1** (C_5D_5N, 150 MHz)

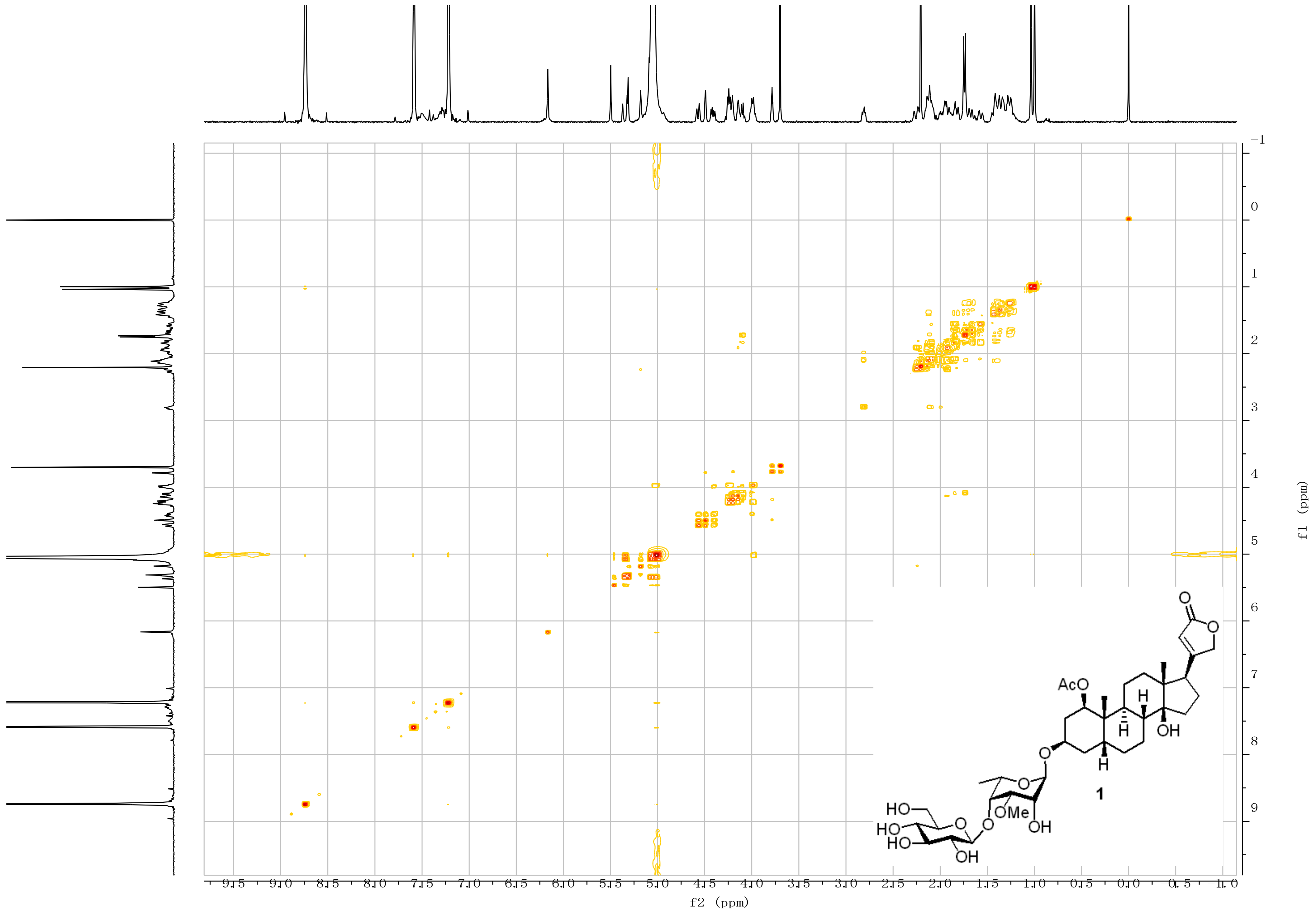

Figure S57 COSY spectrum of compound **1** (C_5D_5N, 600 MHz)

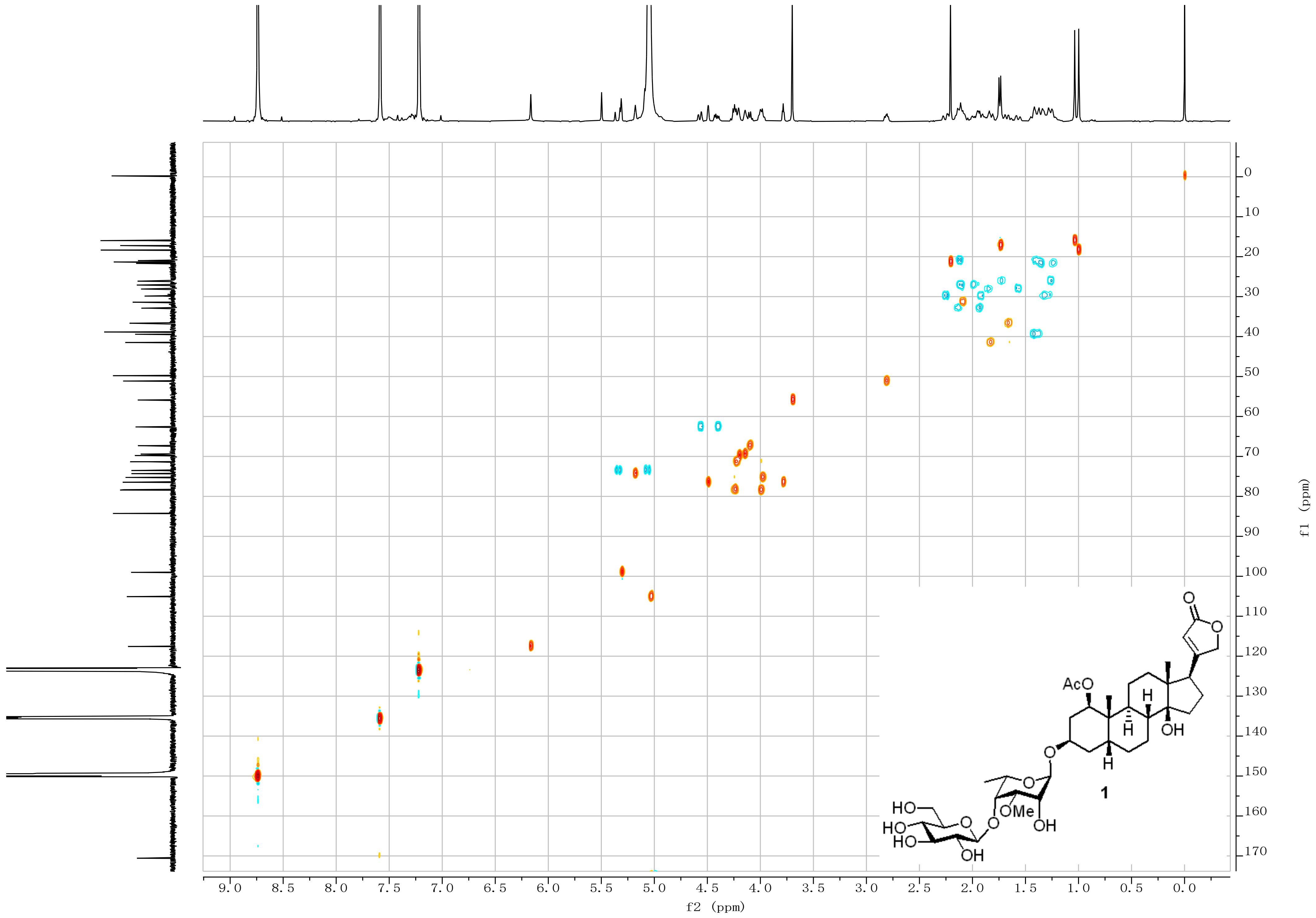

Figure S58 HSQC spectrum of compound **1** (C_5D_5N, 600 MHz)

Comparison of ^{13}C NMR data of the synthetic 1 and 2 with those reported for the natural products

Table S1 Comparison of the Spectroscopic Data of Natural and Synthetic Acovenoside B (2)

^{13}C position	Natural (125 MHz, $CDCl_3$) [1]	Synthetic (150 MHz, $CDCl_3$)	Δδ (ppm)
1	74.2	74.2	0.0
2	29.9	29.9	0.0
3	68.7	68.7	0.0
4	27.8	27.8	0.0
5	31.1	31.1	0.0
6	25.8	25.8	0.0
7	20.6	20.6	0.0
8	41.6	41.7	-0.1
9	35.9	36.9	-1.0
10	38.7	38.8	-0.1
11	21.6	21.6	0.0
12	39.7	39.8	-0.1
13	49.5	49.5	0.0
14	85.3	85.4	-0.1
15	33.1	31.1	0.0
16	26.9	26.9	0.0
17	50.8	50.8	0.0
18	15.8	15.8	0.0
19	18.4	18.5	-0.1
20	174.5	174.5	0.0
21	73.5	73.5	0.0

22	117.8	117.9	-0.1
23	174.4	174.3	0.1
1-OAc	170.7	170.8	-0.1
1-OAc	21.4	21.5	-0.1
1'	97.4	97.4	0.0
2'	68.6	68.6	0.0
3'	75.4	75.4	0.0
4'	70.0	70.1	-0.1
5'	66.2	66.2	0.0
6'	16.5	16.6	-0.1
3'-OMe	55.5	55.6	-0.1

Table S2 Comparison of the Spectroscopic Data of Natural and Synthetic Acospectoside A (1)

^{13}C position	Natural (125MHz, C_5D_5N) [1]	Synthetic (150 MHz, C_5D_5N)	Δδ (ppm)
1	74.5	74.3	-0.2
2	30.0	29.9	-0.1
3	69.6	69.4	-0.2
4	28.2	28.1	-0.1
5	31.5	31.4	-0.1
6	26.2	26.1	-0.1
7	21.0	21.0	0
8	41.5	41.5	0
9	36.7	36.7	0
10	38.9	38.9	0
11	21.8	21.7	-0.1
12	39.5	39.4	-0.1
13	49.9	49.8	-0.1
14	84.4	84.3	-0.1
15	32.9	32.9	0
16	27.2	27.1	-0.1
17	51.2	51.1	-0.1
18	16.1	16.0	-0.1
19	18.4	18.3	-0.1
20	176.0	175.7	-0.3
21	73.7	73.5	-0.2

22	117.6	117.6	0
23	174.7	174.4	-0.3
1-OAc	170.9	170.6	-0.3
1-OAc	21.4	21.3	-0.1
1'	99.1	99.0	-0.1
2'	69.9	69.8	-0.1
3'	76.4	76.4	0
4'	76.4	76.5	0.1
5'	67.4	67.3	-0.1
6'	17.2	17.2	0
3'-OMe	55.9	55.9	0
1"	105.1	105.1	0
2"	75.3	75.3	0
3"	78.2	78.3	0.1
4"	71.4	71.3	-0.1
5"	78.5	78.4	-0.1
6"	62.7	62.6	-0.1

Reference:

1. Hanna, A. G.; Elgamal, M. H. A.; Hassan, A. Z.; Duddeck, H.; Simon, A.; Kovács, J.; Tóth, G. Complete ^{1}H and ^{13}C signal assignments of 5β-cardenolides isolated from *Acokanthera spectabilis* Hook F. *Magn. Reson. Chem.* **1998**, *36*, 936–942.
